# Molecular Characterization of miRNAs in *Myzus persicae* Carrying Brassica Yellows Virus

**DOI:** 10.3390/biology13110941

**Published:** 2024-11-18

**Authors:** Meng-Jun He, Yun Wang, Mei Zhao, Deng-Pan Zuo, You Wang, Zong-Ying Zhang, Ying Wang, Cheng-Gui Han

**Affiliations:** Ministry of Agriculture and Rural Affairs Key Laboratory of Pest Monitoring and Green Management, College of Plant Protection, China Agricultural University, Beijing 100193, China; s20193192583@cau.edu.cn (M.-J.H.); wangyun_0901@163.com (Y.W.); zhaomei@cau.edu.cn (M.Z.); b20173190806@cau.edu.cn (D.-P.Z.); wy1520062280@163.com (Y.W.); zhangzongying@cau.edu.cn (Z.-Y.Z.); yingwang@cau.edu.cn (Y.W.)

**Keywords:** *polerovirus*, brassica yellows virus, green peach aphid, microRNAs, sRNA sequencing, bioinformatics

## Abstract

microRNAs (miRNAs) influence many biological processes at the post-transcriptional level. However, the molecular characterization of miRNAs in the *Myzus persicae* response to Brassica yellows virus (BrYV) stress remains unclear. In this study, we present the results of miRNA profiling in *Myzus persicae* under two different treatments: treatment one (raised on turnip plants), and treatment two (raised on *Arabidopsis thaliana*). A total of 72 known and 113 novel mature miRNAs were identified in both non-viruliferous and viruliferous aphids, under treatment one. In treatment two, 72 known and 112 novel mature miRNAs were identified in BrYV-free aphids; meanwhile, 71 known and 115 novel miRNAs were identified in BrYV-carrying aphids. Moreover, eight upregulated and four downregulated miRNAs were identified in viruliferous aphids under treatment two, whereas only two miRNAs were differentially expressed under treatment one. These results indicated the relative BrYV level could influence miRNA expression in aphids. KEGG enrichment analysis showed the predicted genes targeted by differentially expressed miRNAs were primarily involved in Peroxisome, neuroactive ligand–receptor interaction, and metabolism of xenobiotics by cytochrome P450 pathways. Taken together, these findings reveal the effect of BrYV on miRNAs in *Myzus persicae* and provide key clues for further studies on the molecular mechanisms of BrYV transmission via aphids.

## Simple Summary

Brassica yellows virus, transmitted by the green peach aphid in a persistent circulative manner, is a newly identified polerovirus that is widespread in China. To investigate the molecular characterization of microRNAs (miRNAs) in aphids responding to BrYV stress, non-viruliferous and viruliferous aphids were inoculated to turnip plants (treatment one) or *Arabidopsis thaliana* (treatment two) for 72 h, prior to small RNA sequencing. Numerous known and novel miRNAs in aphids were identified from both treatments. Notably, more differentially expressed miRNAs were identified in viruliferous aphids in the comparisons of treatment two vs. treatment one, with the target genes primarily involved in Peroxisome, neuroactive ligand–receptor interaction, and metabolism of xenobiotics by cytochrome P450 pathways. These findings enhance our understanding of the regulatory role of BrYV on miRNAs in *Myzus persicae* and provide crucial clues for screening key miRNAs involved in virus circulation in aphids.

## 1. Introduction

The diseases caused by numerous plant viruses seriously restrict crop yield and quality, resulting in huge economic losses. More than 80% of plant viruses depend on specific insect vectors for transmission, with aphids being by far the most common and effective, responsible for transmitting nearly 50% of all plant viruses [1,2,3]. Aphid–virus interactions are categorized into four transmission modes based on the time required for virus acquisition, circulation, and inoculation: non-persistent mode [4], semi-persistent mode [5], persistent circulative mode (involving viruses that move from the gut into the hemolymph and other tissues of their vectors), and persistent propagative mode (involving viruses that invade and replicate in various tissues of their vectors) [4,6,7].

Poleroviruses possess a simple positive single-stranded RNA genome and are distributed worldwide, causing huge damage to cereal, vegetable, and fruit crops. In general, virions are restricted to the phloem cells of infected hosts and are mostly transmitted by various aphids in a persistent circulative manner [8,9]. Previous studies have shown the ingestion of virus particles occurs when aphids begin to feed on infected plants, then the virions move upward along the aphid’s alimentary canal. Afterward, ingested virions are able to cross barriers in the gut and accessory salivary gland through the clathrin-mediated endocytosis/exocytosis mechanism, allowing for efficient virus transmission. During the whole process, virus particles are internalized in various vesicular structures; therefore, the actual virus–receptor interactions may occur on the cell membranes of the gut and accessory salivary gland [10,11].

Brassica yellows virus (BrYV), a newly identified polerovirus, is widely distributed in Asia. This virus usually infects cruciferous crops that are also susceptible to the turnip mosaic virus (TuMV) and cucumber mosaic virus (CMV), causing yellowing or leafroll symptoms [12,13,14]. At least three distinct genotypes of BrYV (A, B, and C) have been identified in China, consisting of ORF0, ORF1, ORF2, ORF3a, ORF3, ORF4, and ORF5 [12,15,16]. Previous studies have described the pathway by which poleroviruses circulate in aphids. The major coat protein (CP) and a minor readthrough protein (RTP, a fusion protein with the CP at its N-terminus and the readthrough domain at its C-terminus) have the ability to interact with host receptors on cell membranes. This interaction helps the virions to overcome barriers in the gut and accessory salivary gland. In other words, the structural proteins of poleroviruses are the viral determinants for regulating the specific interaction between BrYV and green peach aphids (*Myzus persicae*) [11,17,18].

As a commonly used model plant, *Arabidopsis thaliana* is a great tool for BrYV research in laboratories. Traditionally, BrYV cannot be transmitted mechanically, and the infection rate of systemic leaves using the *Agrobacterium tumefaciens*-mediated inoculation method is usually below 50%. Therefore, a novel method was established to study the acquisition and transmission of BrYV [19,20], in which BrYV-free aphids acquired BrYV from transgenic line 412 *A. thaliana* plants and from frozen leaves. In addition, when six viruliferous aphids were inoculated on the above plants for 2 days, a 100% BrYV infection rate was observed in the *A. thaliana* plants. This method has been beneficial in preserving viral inocula and enhancing our understanding of the interactions among BrYV, aphids, and hosts. In our previous studies, 1266 differentially expressed genes (DEGs) and 18 differentially expressed proteins (DEPs) were identified in aphids carrying BrYV. An analysis indicated that these DEGs and DEPs were mainly enriched in epidermal protein synthesis, phosphorylation, and various metabolic processes. Interestingly, a number of cuticle proteins and tubulins were significantly upregulated in viruliferous aphids, suggesting a potentially positive effect on the acquisition or transmission of BrYV by aphids [21].

As a conserved class of small non-coding RNAs, miRNAs were first observed in roundworms (*Caenorhabditis elegans*) and typically ranged from 19 to 25 nucleotides (nt) in length [22,23]. In animal cells, miRNAs firstly identify target genes primarily through “seed sequences”, then Argonaute (AGO) proteins are recruited to form RNA-induced silencing complexes (RISCs), playing a key regulatory role in gene expression at the post-transcriptional level [24]. Previous studies have shown that miRNAs are key regulators of many biological processes in insects, such as development, reproduction, metamorphosis, immunity, and insecticide resistance [25]. Owing to the important function of miRNAs in plant responses to biotic and abiotic stresses, miRNA-based genetic modification technology has become an effective tool for improving crops and developing novel crop cultivars [26]. For example, abnormal insect molting and larvae death occurred when cotton bollworm (*Helicoverpa armigera*) fed on transgenic tobacco plants with high levels of amiR-24 targeting the chitinase gene [27]. The overexpression of the miR166b targeting ATP synthase gene of whiteflies (*Bemisia tabaci*) in upland cotton (*Gossypium hirsutum*) has been shown to effectively reduce the population of *B. tabaci* and limit the spread of plant viruses by vector control [28]. Additionally, transgenic microRNA-14 rice, designed to target Spook (Spo) and the Ecdysone receptor (EcR) in the ecdysone signaling network, showed high resistance to *Chilo suppressalis*, demonstrating the potential of utilizing miRNAs as targets for pest control [29].

Aphid miRNAs have been identified in different species. The first miRNAs from a hemipteran insect were identified through the genomic analysis of the genome of the pea aphid (*Acyrthosiphon pisum*). A total of 149 miRNAs including 55 conserved and 94 new miRNAs were identified using solexa sequencing and bioinformatic analyses [30]. In addition, 45 miRNAs were identified from the English grain aphid (*Sitobion avenae*). More interestingly, 16 miRNAs were significantly upregulated and 12 miRNAs were downregulated in winged aphids compared to wingless aphids, suggesting their involvement in the metabolism, development, and wing polyphenism in *S. avenae* [31]. Furthermore, a total of 66 known miRNAs were identified in the tobacco aphid (*Myzus persicae nicotianae*), with *let-7* and *miR-100* miRNAs predicted to target the CYP6CY3 gene in aphids. The tolerance of aphids to nicotine was altered when they ingested the inhibitors of let-7 and miR-100 miRNAs, which confirmed the regulatory roles of these two miRNAs [32].

At present, the molecular characterization of miRNAs and the effect of BrYV on miRNAs in *M. persicae* remains unclear. Here, we analyzed the expression profiles of miRNAs in BrYV-free and BrYV-carrying aphids under two treatments. Our study clarifies the molecular characteristics of miRNAs induced by BrYV and enhances our understanding of how *M. persicae* responds to BrYV stress at the post-transcriptional level. This discovery provides key clues for further studies on the molecular mechanisms of virus transmission.

## 2. Materials and Methods

### 2.1. Aphid Culture and Plant Materials

Apterous aphids used in this study were collected from the west campus of China Agricultural University and maintained on the turnip variety ‘Yamei No. 1’. To ensure sufficient nutrition for aphids, we replaced the seedlings every two weeks. The aphids were reared in incubators maintained at 22 ± 2 °C, 60% relative humidity, and 14 h of light per day.

The transgenic *A. thaliana* line 412 was obtained by transforming a 35S promoter-driven expression cassette containing the full-length cDNA of BrYV [20]. Notably, the transgenic line 412 exhibited abnormal phenotypes compared with the Col-0 ecotype, including late flowering, loss of apical dominance, purple coloration of lower leaves, and a notable increase in the number of rosette leaves. These transgenic plants were used as viral inoculum for aphids. All plants were grown in a greenhouse at 24 °C, with a photoperiod cycle of 10 h light/14 h darkness and 60% relative humidity.

### 2.2. Sample Preparation

The original aphids were collected in October 2017 and maintained on healthy turnip plants. In addition, partial samples were periodically tested to ensure the reared aphids were BrYV-free. To obtain non-viruliferous and viruliferous aphids, abundant second instar apterous aphids were inoculated to Col-0 and transgenic *A. thaliana* line 412 plants. After 72 h of feeding, a portion of aphids were transferred to turnip plants (treatment 1) or Col-0 (treatment 2) for 72 h. Then, samples were collected for RNA extraction, followed by reverse transcription-polymerase chain reaction (RT-PCR) verification using specific primers designed for BrYV detection (Figure S1). Four (treatment 1) and three (treatment 2) biological replicates for each group were prepared for sRNA sequencing analysis, and each replicate contained a mix of 20 aphids.

### 2.3. Analysis of sRNA Sequencing Data

Total RNA was extracted using a TRIzol reagent (Invitrogen, San Diego, CA, USA). After assessing purity and integrity, sRNA libraries for non-viruliferous and viruliferous were constructed using a small RNA sample pre-kit (Vazyme, Nanjing, China). Next, after purification, libraries with insertions between 18 bp and 40 bp were selected for sequencing on the Illumina sequencing platform with SE50.

Raw data files from sRNA sequencing were initially subjected to base calling analysis to generate raw reads. After the removal of adapter sequences and low-quality reads, clean reads were obtained. Subsequently, sRNAs ranging in size from 18 nt to 35 nt were selected for further analysis. The small RNA tags were then mapped to the reference sequence of *M. persicae* using Bowtie (version 1.0.1). Bowtie was used to preliminarily screen reads, and then the mapped small RNA tags were used to search for known miRNAs using miRbase 22.0 as a reference. After removing tags originating from protein-coding genes, repeat sequences, rRNA, tRNA, snRNA, and snoRNA, and novel miRNA prediction, the quantitative analysis was performed using mirdeep2.0.0.5.

### 2.4. Molecular Characterization of miRNAs

To identify known miRNAs, sRNAs mapped to the green peach aphid genome were blasted against the data of *A. pisum* in the miRbase website “https://www.mirbase.org/” (accessed on 3 May 2022) using modified software mirdeep2 (mirdeep2.0.0.5) [33] and srna-tools-cli [34]. In addition, the novel miRNAs were predicted by analyzing the secondary structure, the Dicer cleavage site, and the minimum free energy of unannotated small RNA tags, using the available software miREvo (version 1.1) [35] and mirdeep2. Specifically, miRNA quantitative analysis was performed using mirdeep2.0.0.5 software, and expression levels were estimated by TPM (transcript per million). Two libraries were created for viruliferous and non-viruliferous aphids in our study, and then these data were combined for miRNA annotation and used for quantification analysis, respectively. Differential expression levels between BrYV-free and BrYV-carrying aphids were analyzed using Deseq2 (version 1.24.0) [36], with a significance threshold set at a p value of 0.05 for differentially expressed mature miRNAs. The target genes of known and novel miRNAs were predicted using miRanda (v3.3a) and RNAhybrid (v2.0) software. Commonly predicted genes from two software were selected as the targeted results for further analysis.

### 2.5. miRNA Expression Verification by qPCR

Quantitative PCR (qPCR) was used to verify miRNA expression levels. Given the short length of mature miRNAs, traditional PCR methods are not suitable for direct detection. Therefore, the miRNA 1st strand cDNA synthesis kit (by tailing A) (Vazyme, Nanjing, China) was employed to synthesize the cDNA. Briefly, total RNA containing miRNAs was extracted using the TRIzol reagent as described previously by Zuo et al. [19], and then the cDNA was synthesized using 1.5 µg of DNase-treated RNA. Specifically, Poly(A) was first added to the 3’ end of miRNAs, and reverse transcription was performed using universal primers included in the kit. Each reaction system (20 µL) comprised 10 µL 2 × miRNA RT mix, 2 µL Hiscript miRNA Enzyme Mix, and RNase-free H_2_O. The reaction program consisted of incubation at 37 °C for 60 min, followed by 85 °C for 5 min.

Subsequently, qPCR analysis with at least two biological replicates (each biological replicate contained three technical replicates) was performed to validate the relative expression of miRNAs. Forward primers were designed based on the sequencing of miRNAs Table S1), with the universal primer in the kit serving as the reverse primer for qPCR detection using GoTaq^®^ qPCR Master Mix (Promega, Madison, WI, USA). Each reaction system contained 10 µL 2 × SYBR Green real-time PCR Master Mix, 0.5 µL forward primer (10 µM), 0.5 µL reverse primer (10 µM), 1 µL cDNA, and 8 µL RNase-free ddH_2_O. The reaction program consisted of an initial denaturation at 95 °C for 2 min, followed by 40 cycles of 95 °C for 15 s, 60 °C for 1 min, and 72 °C for 30 s. The actin gene was used as an internal reference [37], and the relative gene expression levels were calculated using the 2^−∆∆CT^ method [38,39].

### 2.6. Analysis of Go and KEGG Enrichment

Gene Ontology (GO) term enrichment analysis was performed using the software clusterProfiler (v3.8.1), focusing on three main categories: the biological process, cellular component, and molecular function. In addition, *p* value < 0.05 was used as the criterion. Subsequently, the target genes studied were blasted against the online Kyoto Encyclopedia of Genes and Genomes (KEGG) database “http://geneontology.org/ (accessed on 23 January 2022)”.

## 3. Results

### 3.1. BrYV Had No Significant Effect on sRNA Distribution in M. persicae

**Figure 1:**
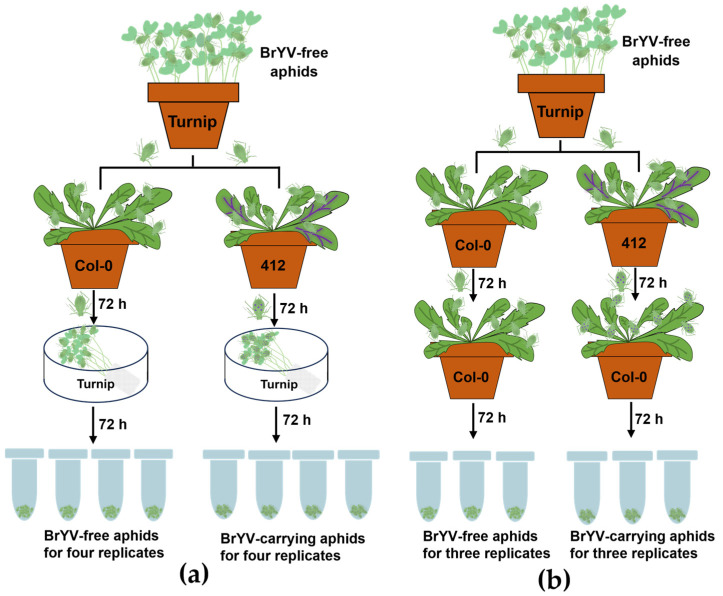
Schematic diagram of sRNA-seq design for treatment one (**a**) and treatment two (**b**). Aphids were given a 72 h acquisition access period (AAP) on Col-0 or BrYV transgenic A. thaliana line 412 plants, followed by a 72 h feeding period on turnip plants for four replicates (**a**) or Col-0 for three replicates (**b**). Purple icosahedrons were used to represent viruliferous aphids. Each replicate included 20 individuals.

Previous studies have shown that the potato leafroll virus (PLRV, a representative virus of the genus *polerovirus*) replication does not occur in turnip plants, making it an effective tool for gut clearing in aphids carrying PLRV within just 3 days [40]. Building on these findings, turnip is verified as a nonhost of BrYV-C as well (Figure S2). Thus, we transferred BrYV-free and BrYV-carrying samples to turnip plants for a 72 h gut clearing (treatment one, Figure 1a). Meanwhile, non-viruliferous and viruliferous aphids were also transferred to Col-0 for 72 h (treatment two, Figure 1b).

For treatment one, on average, 8,524,026 and 8,555,278 clean reads were obtained from non-viruliferous and viruliferous aphids, respectively. Of these, 4,872,049 and 4,754,803 clean reads were successfully mapped to the genome of *M. persicae*, accounting for 57.16% and 55.58% of the total reads, respectively (Table 1). For treatment two, we obtained, on average, 7,688,479 and 7,135,038 clean reads from non-viruliferous and viruliferous samples, respectively. Of these, 4,456,846 and 4,109,897 clean reads were mapped to the genome of *M. persicae*, accounting for 57.97% and 57.60% of the total reads (Table S2).

**Figure 2:**
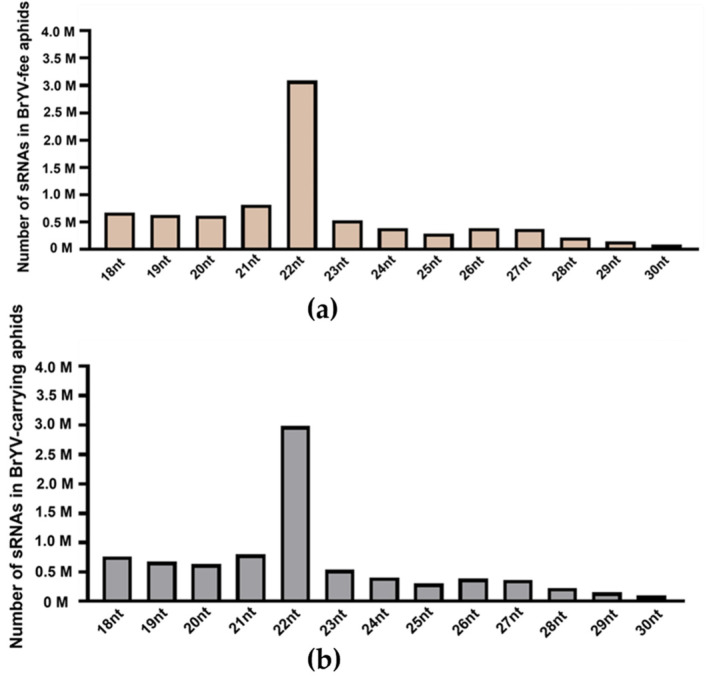
Size distribution of sRNAs in BrYV-free (**a**) and BrYV-carrying aphids (**b**) under treatment one. The X-axis represents the length of sRNAs and “M” denotes million reads.

As shown in Figure 2a,b, the size of sRNAs ranged predominantly from 18 nt to 22 nt, with a peak at 22 nt under treatment one. Furthermore, these results were consistent with the results from treatment two (Figure S3a,b). Thus, the distribution of host sRNAs did not show significant changes in aphids carrying BrYV.

### 3.2. Molecular Characterization of miRNAs of M. persicae

**Figure 3:**
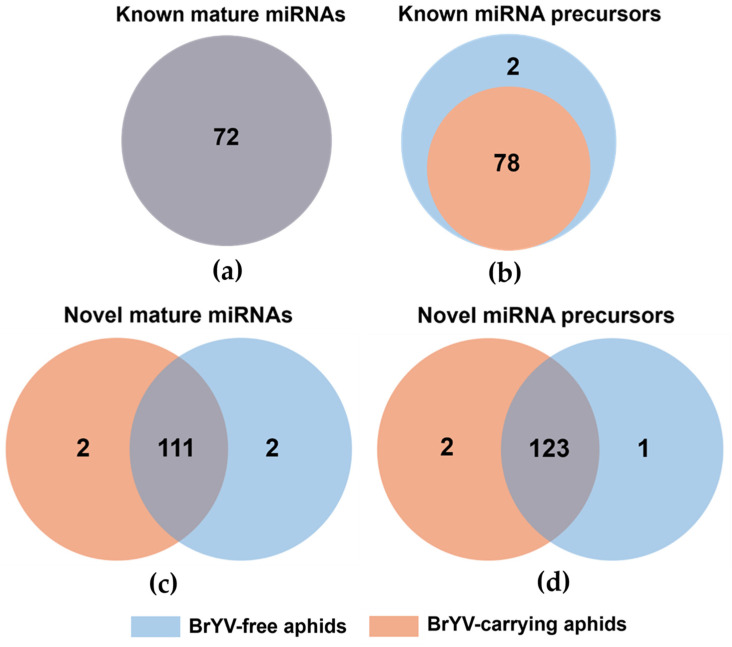
Identification of known and novel miRNAs in BrYV-free and BrYV-carrying aphids under treatment one. The Venn diagram illustrates the number of known (**a**,**b**) and novel (**c**,**d**) mature miRNAs (**a**,**c**) or miRNA precursors (**b**,**d**) that were unique to either non-viruliferous or viruliferous aphids, as well as those shared between the two groups.

A total of 72 known mature miRNAs (with a dominant nucleotide of U at the 5′ end) and 80 known precursors were identified in BrYV-free aphids that had fed on turnip plants (treatment one). On the other hand, 72 mature miRNAs and 78 known precursors were identified in viruliferous aphids (Figure 3a,b; Data S1). At the same time, 113 novel mature miRNAs and 124 miRNA precursors were predicted in non-viruliferous aphids. Moreover, 113 novel mature miRNAs and 125 miRNA precursors were predicted in viruliferous aphids. Among these, 111 novel miRNAs and 123 precursors were found in both groups (Figure 3c,d; Data S1 and S2). For aphids fed on Col-0 (treatment two), a total of 72 mature miRNAs, 80 precursors, 112 novel miRNAs (mature miRNAs), and 132 precursors were identified in non-viruliferous aphids (Data S1 and S3). Meanwhile, the corresponding numbers identified in viruliferous aphids were 71, 74, 115, and 131, respectively (Figure S3c–f; Data S1 and S3).

Additionally, a total of 117 conserved miRNA sequences were identified in other species in the miRBase database under treatment one. The mir-92_2, mir-2, and mir-9 were predicted to have the highest number of family members (n = 4), followed by mir-10, mir-87, mir-279, mir-3688, mir-216, and mir-3017 (n = 3). For treatment two, a total of 65 conserved miRNAs were identified, mainly from mir-2 and mir-92_2 families (n = 4), followed by mir-10, mir-184, mir-279, and mir-3017 (n = 3).

### 3.3. Differential miRNA Expression in Viruliferous and Non-Viruliferous Aphids

**Figure 4:**
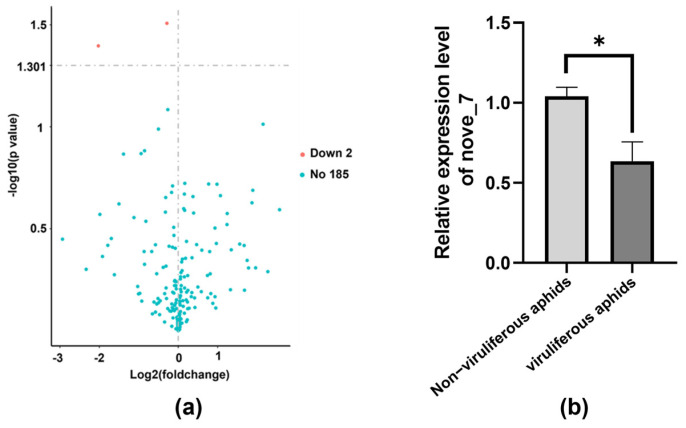
Characterization of differentially expressed miRNAs in *M. persicae* under treatment one. (**a**) Analysis of the miRNAs that showed differential expression between viruliferous and non−viruliferous aphids, with a threshold set at |log2 (fold change, FC)| > 0 and *p* value < 0.05. (**b**) The relative expression level of novel_7 was verified using qPCR (* indicates *p* value < 0.05). Actin was used as an internal reference.

Compared to BrYV-free samples, two downregulated miRNAs were identified in viruliferous aphids under treatment one, namely novel_213 and novel_7, based on the criteria of |log2 (Fold Change, FC)| > 0 and *p* value < 0.05 (Figure 4a). Due to the low read counts of novel_213 in aphids, we focused on verifying the relative expression level of novel_7 by qPCR, which showed consistency with the trends observed in sRNA sequencing (Figure 4b). Unfortunately, only ”*Myzus persicae* uncharacterized LOC111041711” (NCBI accession number: XR_002605355.1) was predicted to be the target of novel_7; thus, further enrichment analysis could not be conducted.

**Figure 5:**
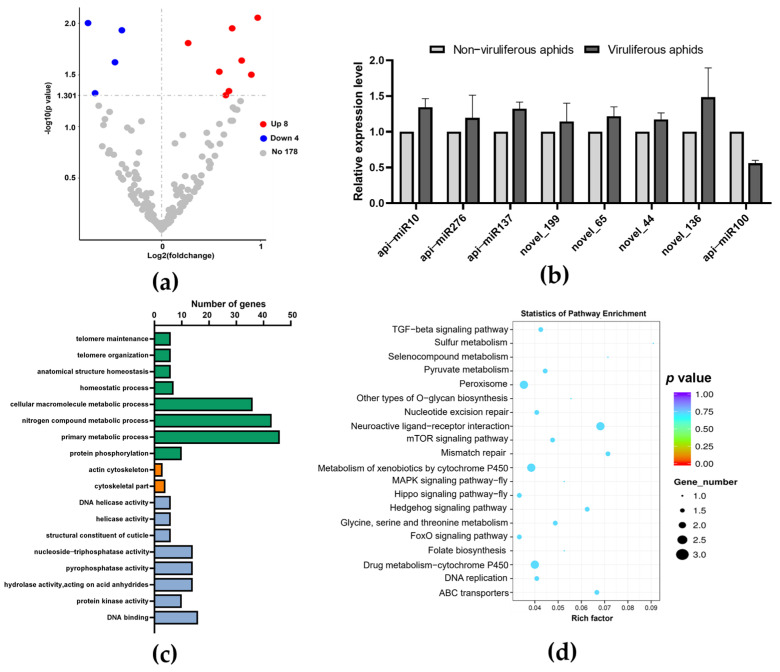
Characterization of differentially expressed miRNAs in aphids under treatment two. (**a**) The differentially expressed miRNAs between viruliferous vs. non−viruliferous aphids using a threshold |log2 (fold change, FC)| > 0 and *p* value < 0.05. (**b**) Eight differentially expressed miRNAs were selected randomly to verify the reliability of sRNA−seq results using qPCR with three biological replicates, and each biological replicate contained three technical replicates. Actin was used as an internal reference. (**c**) GO enrichment analysis. The X−axis represents the enriched GO terms (*p* value < 0.05) including biological process (BP), cellular component (CC), and molecular function (MF). (**d**) KEGG enrichment analysis of aphid genes potentially targeted by differentially expressed miRNAs.

For aphids fed on Col-0, 12 differentially expressed miRNAs (eight upregulated and four downregulated) were identified in aphids carrying BrYV, using a cutoff of |log2 (fold change, FC)| > 0 and *p* value < 0.05 (Figure 5a). In addition, we selected four known and four novel miRNAs for qPCR verification. Overall, the qPCR results were consistent with sRNA sequencing analyses (Figure 5b).

Subsequently, functional annotation of putative genes targeted by differentially expressed miRNAs indicated that “telomere maintenance”, “anatomical structure homeostasis”, “cellular macromolecule metabolic process”, “nitrogen compound metabolic process” and “protein phosphorylation” were the major enriched biological process (BP) terms. In addition, the terms “DNA helicase activity”, “structural constituent of cuticle”, “pyrophosphatase activity”, and “DNA binding” were highly enriched in aspect molecular function (MF) (Figure 5c). KEGG enrichment analysis showed these target genes were mainly involved in “Peroxisome”, “Neuroactive ligand–receptor interaction”, “Metabolism of xenobiotics by cytochrome P450” and “Drug metabolism-cytochrome P450” pathways (Figure 5d).

## 4. Discussion

In the present study, we examined the expression profiles of miRNAs in both BrYV-free and BrYV-carrying *M. persicae*. Our findings showed a total of 72 known and 113 novel mature miRNAs were identified both in non-viruliferous and viruliferous aphids, under treatment one. For treatment two, 72 known and 112 novel mature miRNAs were identified in BrYV-free aphids; meanwhile, 71 known and 115 novel miRNAs were identified in aphids carrying BrYV. In addition, these mature miRNAs exhibited a strong bias for the nucleotide U at the 5′ end, consistent with previous findings on *A. pisum* and English grain aphid *Sitobion avenae* [30,31]. A previous study has shown 66 known miRNAs were identified in *M. persicae nicotianae*, reared on tobacco (*Nicotiana tabacum* L.) [32]. In our results, 72 known miRNAs were identified in non-viruliferous aphids, raised on turnip plants (Yamei No. 1), among which 64 known mature miRNAs were common to both treatments. Eight miRNAs were unique to our data while two were unique to *M. persicae nicotianae* (Table S3).

In our previous studies, 1266 DEGs (980 upregulated and 286 downregulated DEGs) were identified in BrYV-carrying aphids compared to BrYV-free samples [21], whereas the number of differentially expressed miRNAs identified in this study was much lower than that of DEGs. miRNAs are short RNA molecules, 19 to 25 nucleotides in length. Due to the complex biogenesis of miRNAs, these identified DEGs might not form double-stranded RNAs that induce silencing within viruliferous aphids. In addition, the relative BrYV levels were reduced in viruliferous aphids due to the absence of new virus sources on turnip plants under treatment one. For aphids fed on Col-0, the viruliferous aphids could acquire new virions by piercing the BrYV-infected *A. thaliana* continuously. It is likely that the increase in BrYV levels was favorable for viral particles to be actively transported through the gut epithelial cells and released into the hemocoel, which might cause the differences in the expression profiles of miRNAs between the two treatments.

The family analysis of known and novel miRNAs was performed with other species in the miRBase database. The result showed mir-2 had the highest number of family members in both treatments. Similarly, the miR-2 family was predicted to have the most family members (n = 8) in *S. avenae* [31]. In the pea aphid, *A. pisum*, Ap-mir2a-1 was significantly upregulated in parthenogenetic morphs compared to oviparae [30]. In *Drosophila*, the miR-2/13 may play a role in regulating apoptotic cells in embryos [41]. In the silkworm, *Bombyx mori*, two genes, the *abnormal wing disc* (awd) and *fringe* (fng), were predicated as target genes of miR-2 and were involved in Notch signaling. Transgenic studies indicated the essential functions of miR-2 in regulating wing morphogenesis [42].

Enrichment analysis indicated the genes targeted by differentially expressed miRNAs were primarily involved in “Metabolism of xenobiotics by cytochrome P450”and “Drug metabolism–cytochrome P450”pathways. As shown in the previous study, the cytochrome P450 gene was associated with resistance to neonicotinoid insecticides in *M. persicae*. Compared to the susceptible clone, the EST sequences, encoding cytochrome P450s, which were similar to a single cytochrome P450 gene of *A. pisum* CYP6CY3, were elevated in the insecticide-resistant clone of *M. persicae* [43]. Interestingly, compared to non-viruliferous aphids, the expression of cytochrome P450-like protein was lower for viruliferous aphids fed on turnip yellows virus (TuYV)-infected plants or on an artificial medium containing purified virus particles. The reason for this difference and whether cytochrome P450-like protein was associated with the transmission of TuYV via aphids still required further analysis [44]. In RNA-seq data, three P450 genes were significantly upregulated, namely cytochrome P450 6k1-like (NW_019101461.1), probable cytochrome P450 6a13 (NW_019103749.1), and probable cytochrome P450 6a14 (NW_019103956.1), and one P450 gene was significantly downregulated, namely cytochrome P450 4C1-like (NW_019104013.1), in the comparisons between BrYV-carrying and BrYV-free aphids. Further, their function in the transmission of BrYV could be explored by RNA interference [21].

As one of the critical factors, wing formation in aphids was supposed to be responsible for the transmission of plant viruses and the spread of viral diseases. Interestingly, numerous studies have shown the regulatory role of miRNAs in wing development in aphids. For example, the expression level of aci-miR-9b was lower in the winged brown citrus aphid (*Aphis citricidus*). In addition, aci-miR-9b could induce a high proportion of winged aphids by targeting the ABC transporter (ABCG4), which activates the insulin and insulin-like signaling pathway [45]. Furthermore, piRNAs derived from Y-satellite RNA (Y-sat) of the cucumber mosaic virus promoted the presence of alates by interfering with the binding of miR9b to ABCG4 mRNA in aphids, facilitating the spread and transmission of CMV in the field [46]. miR-92a-1-p5 in *A. pisum* regulated flight muscle formation and wing extension by manipulating the expression of the flightin gene [47]. Insect vectors often have greater fitness on virus-infected plants. For example, PLRV has been shown to increase vector attraction and change the probing behavior of aphids [48,49,50]. Moreover, PLRV increased vector fecundity and attenuated the induction of aphid-induced jasmonic acid and ethylene in *Nicotiana benthamiana* and potato (*Solanum tuberosum*). Additionally, three PLRV proteins (P0, P1, and P7) were considered to mediate these changes in plant–aphid interactions [51]. Interestingly, it would be meaningful to investigate whether BrYV has the ability to promote vector performance by regulating the expression of miRNAs based on our sequencing data.

## 5. Conclusions

In the present study, we described the molecular characteristics of miRNAs in the comparisons of BrYV-carrying vs. BrYV-free aphids. Compared to treatment one, more differentially expressed miRNAs were identified under treatment two, which means the relative BrYV level could influence the expression profiles of miRNAs in aphids carrying BrYV. Notably, many predicted target genes were enriched in functions related to Peroxisome, neuroactive ligand–receptor interaction, and metabolism of xenobiotics by cytochrome P450 pathways. Therefore, RNA interference [52,53] could be used to test the roles of these genes in the acquisition and transmission of BrYV. Taken together, our findings enrich our understanding of *M. persicae* in response to BrYV stress at the post-transcriptional level and provide valuable insights for future research into the molecular mechanisms underlying BrYV transmission.

## Figures and Tables

**Table 1:** Summary of small RNA sequencing results under treatment one.

Items	Non-Viruliferous Aphids	Viruliferous Aphids
Raw reads number	10,550,213	11,339,317
Q30 of raw reads (%)	97.69	97.83
GC content (%)	48.49	48.73
Clean reads number	10,360,386	11,145,842
Total reads ^a^	8,524,026	8,555,278

^a^ The number of clean reads in length from 18 nt to 25 nt.

## Data Availability

The data presented in this study are available within the article.

## Supplementary Materials

The following supporting information can be downloaded at https://www.mdpi.com/article/10.3390/biology13110941/s1. Figure S1. Confirmation of the presence of BrYV in viruliferous and non-viruliferous aphids using RT-PCR. Figure S2. Turnip is verified as a nonhost of BrYV-C. Figure S3. Host-derived sRNAs in BrYV-free and BrYV-carrying aphids under treatment two. Table S1. Primers used for BrYV detection and RT-qPCR validation. Table S2. Summary of small RNA sequencing results under treatment two. Table S3. The known mature miRNAs identified in our data and *M. persicae nicotianae*. Data S1. The known and novel miRNAs under two treatments. Data S2. Structures of novel miRNAs under treatment one. Data S3. Structures of novel miRNAs under treatment two.
