# Supplementary figures and images for: Molecular Characterization of miRNAs in Myzus persicae Carrying Brassica Yellows Virus (part 2 of 2)
Source: Biology (Basel). 2024 Nov 18;13(11):941. doi: 10.3390/biology13110941 (PMC11591976; doi:10.3390/biology13110941)

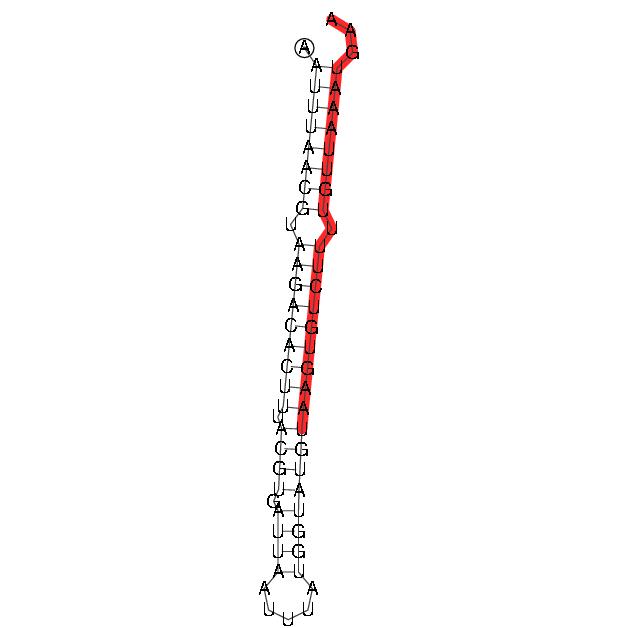

Supplement: Supplementary file 1 [file biology-13-00941-s001.zip › Data S2. Structures of novel miRNAs under treatment 1/novel_187_novel_187.jpg]

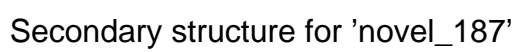

Supplement: Supplementary file 1 [file biology-13-00941-s001.zip › Data S2. Structures of novel miRNAs under treatment 1/novel_187_novel_187.pdf]

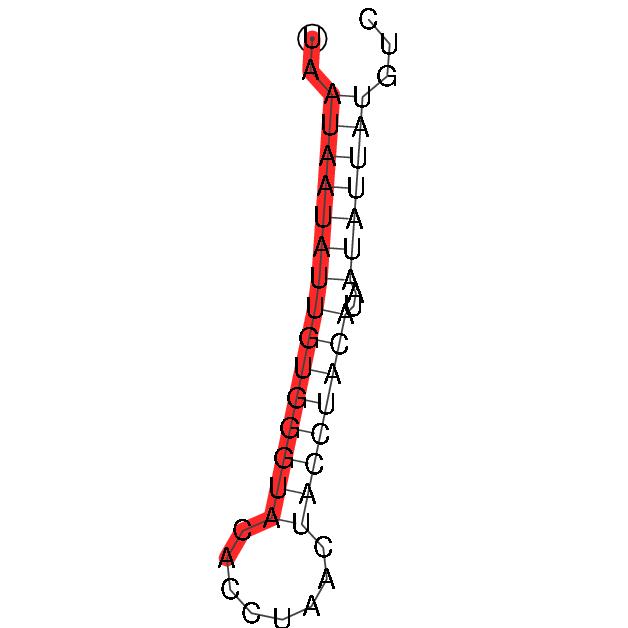

Supplement: Supplementary file 1 [file biology-13-00941-s001.zip › Data S2. Structures of novel miRNAs under treatment 1/novel_190_novel_190.jpg]

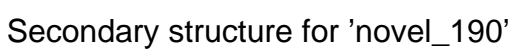

Supplement: Supplementary file 1 [file biology-13-00941-s001.zip › Data S2. Structures of novel miRNAs under treatment 1/novel_190_novel_190.pdf]

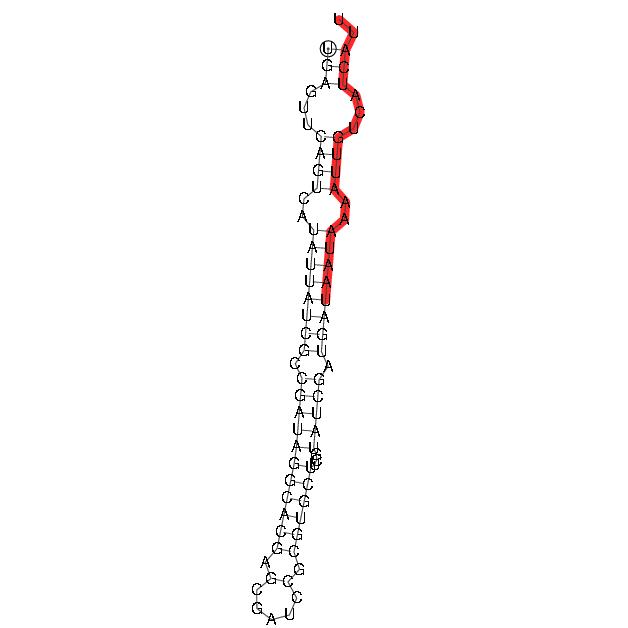

Supplement: Supplementary file 1 [file biology-13-00941-s001.zip › Data S2. Structures of novel miRNAs under treatment 1/novel_191_novel_191.jpg]

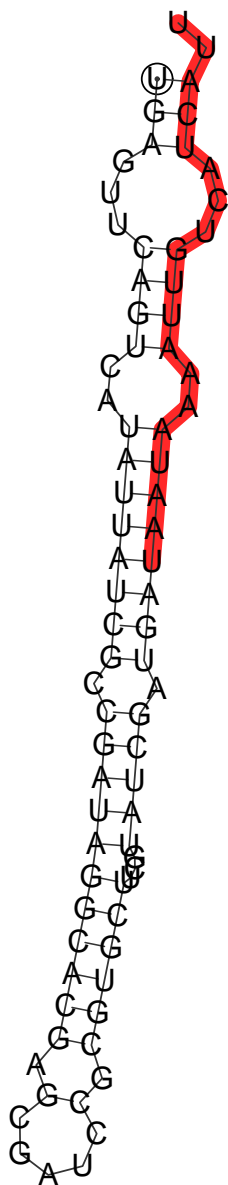

Secondary structure for 'novel\_191'

Supplement: Supplementary file 1 [file biology-13-00941-s001.zip › Data S2. Structures of novel miRNAs under treatment 1/novel_191_novel_191.pdf]

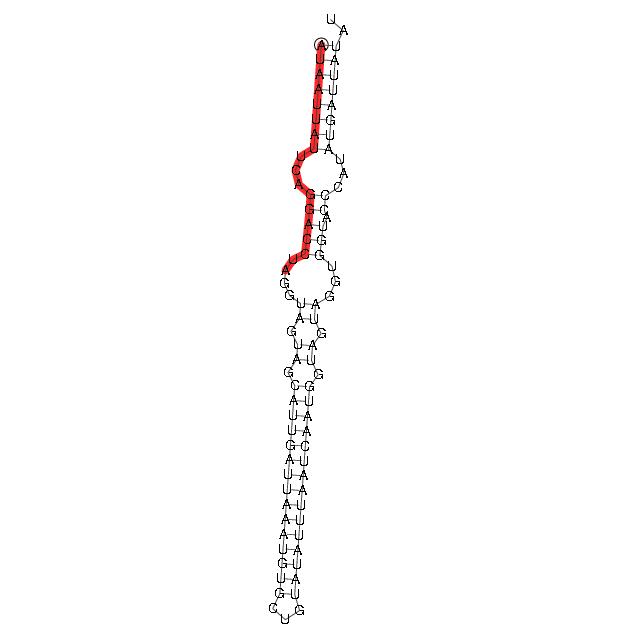

Supplement: Supplementary file 1 [file biology-13-00941-s001.zip › Data S2. Structures of novel miRNAs under treatment 1/novel_193_novel_193.jpg]

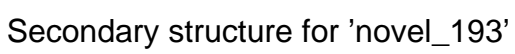

Supplement: Supplementary file 1 [file biology-13-00941-s001.zip › Data S2. Structures of novel miRNAs under treatment 1/novel_193_novel_193.pdf]

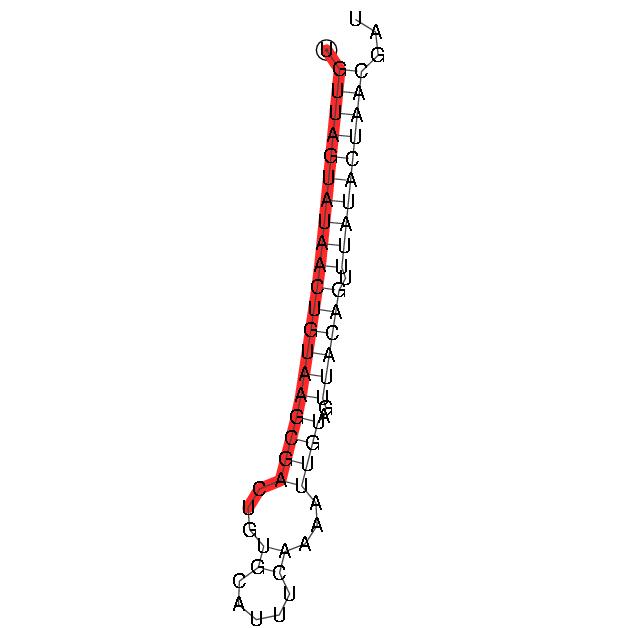

Supplement: Supplementary file 1 [file biology-13-00941-s001.zip › Data S2. Structures of novel miRNAs under treatment 1/novel_194_novel_194.jpg]

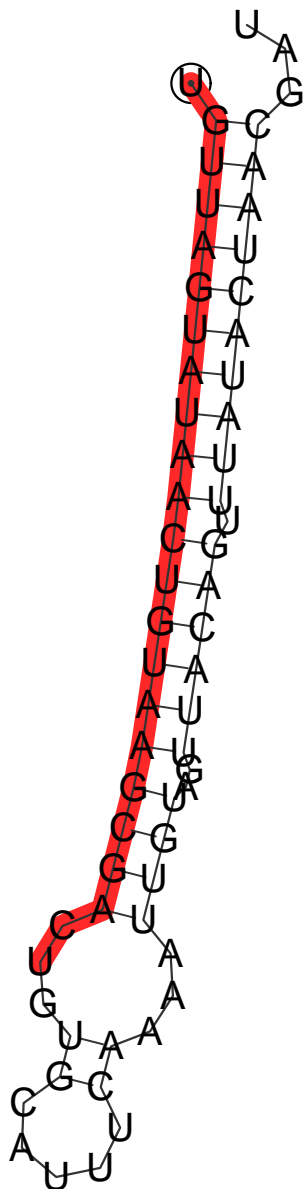

Secondary structure for 'novel\_194'

Supplement: Supplementary file 1 [file biology-13-00941-s001.zip › Data S2. Structures of novel miRNAs under treatment 1/novel_194_novel_194.pdf]

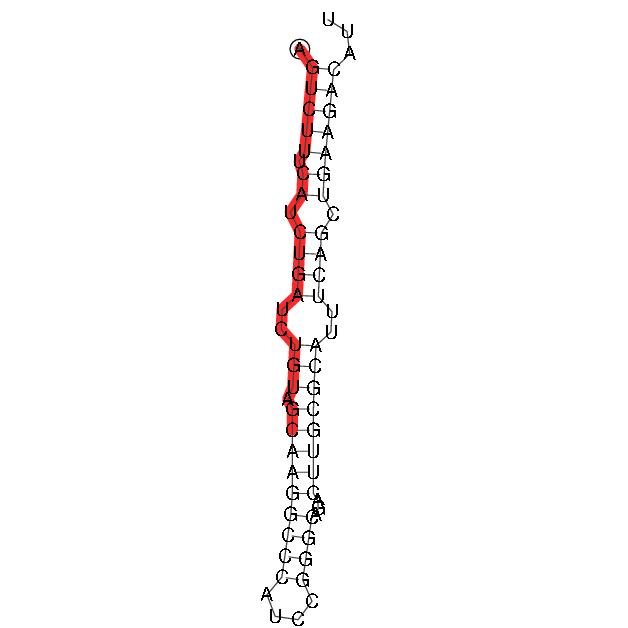

Supplement: Supplementary file 1 [file biology-13-00941-s001.zip › Data S2. Structures of novel miRNAs under treatment 1/novel_195_novel_195.jpg]

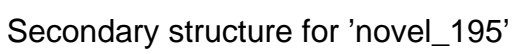

Supplement: Supplementary file 1 [file biology-13-00941-s001.zip › Data S2. Structures of novel miRNAs under treatment 1/novel_195_novel_195.pdf]

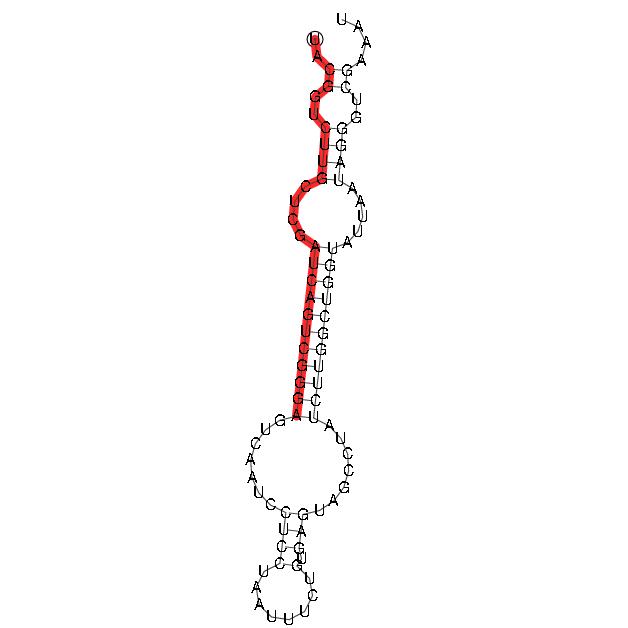

Supplement: Supplementary file 1 [file biology-13-00941-s001.zip › Data S2. Structures of novel miRNAs under treatment 1/novel_196_novel_196.jpg]

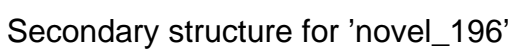

Supplement: Supplementary file 1 [file biology-13-00941-s001.zip › Data S2. Structures of novel miRNAs under treatment 1/novel_196_novel_196.pdf]

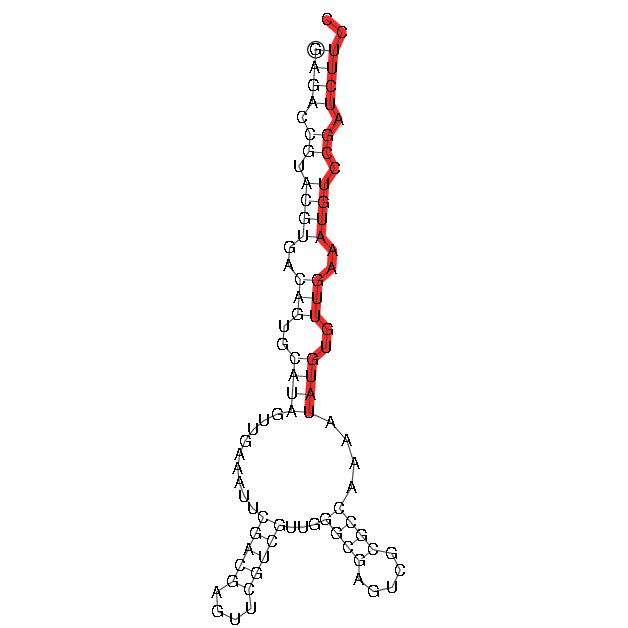

Supplement: Supplementary file 1 [file biology-13-00941-s001.zip › Data S2. Structures of novel miRNAs under treatment 1/novel_197_novel_197.jpg]

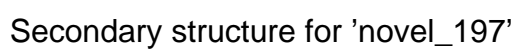

Supplement: Supplementary file 1 [file biology-13-00941-s001.zip › Data S2. Structures of novel miRNAs under treatment 1/novel_197_novel_197.pdf]

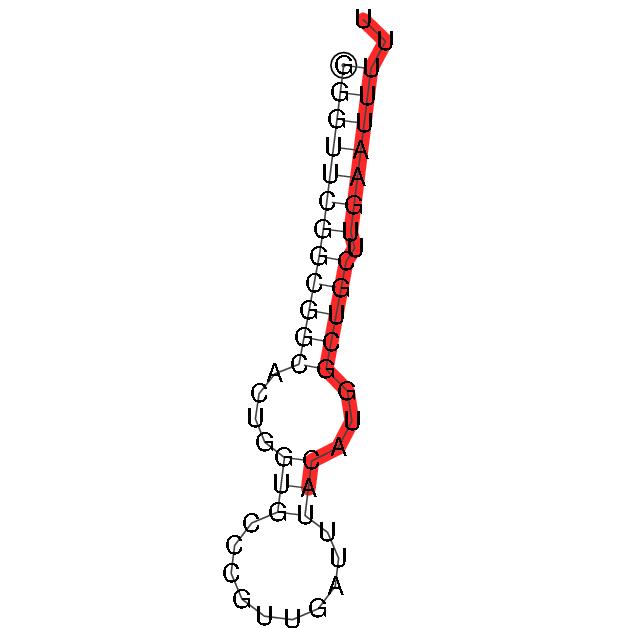

Supplement: Supplementary file 1 [file biology-13-00941-s001.zip › Data S2. Structures of novel miRNAs under treatment 1/novel_198_novel_198.jpg]

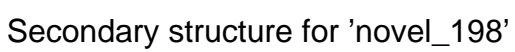

Supplement: Supplementary file 1 [file biology-13-00941-s001.zip › Data S2. Structures of novel miRNAs under treatment 1/novel_198_novel_198.pdf]

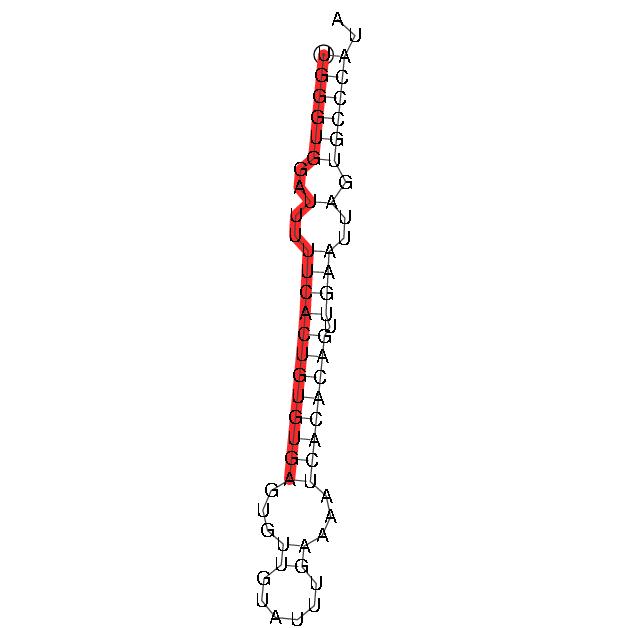

Supplement: Supplementary file 1 [file biology-13-00941-s001.zip › Data S2. Structures of novel miRNAs under treatment 1/novel_201_novel_201.jpg]

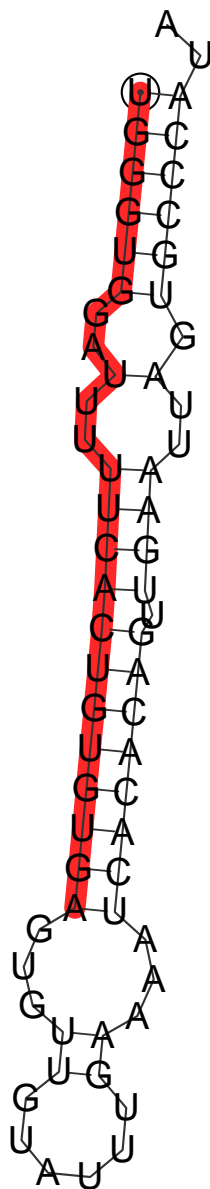

Secondary structure for 'novel\_201'

Supplement: Supplementary file 1 [file biology-13-00941-s001.zip › Data S2. Structures of novel miRNAs under treatment 1/novel_201_novel_201.pdf]

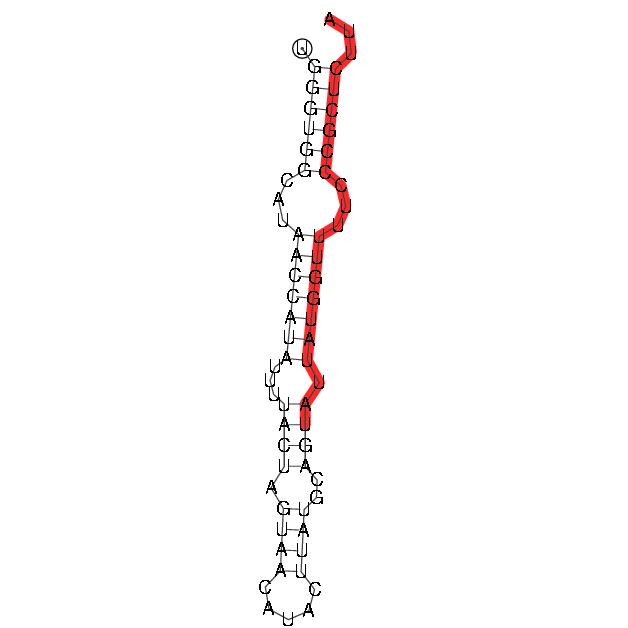

Supplement: Supplementary file 1 [file biology-13-00941-s001.zip › Data S2. Structures of novel miRNAs under treatment 1/novel_202_novel_202.jpg]

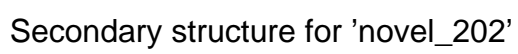

Supplement: Supplementary file 1 [file biology-13-00941-s001.zip › Data S2. Structures of novel miRNAs under treatment 1/novel_202_novel_202.pdf]

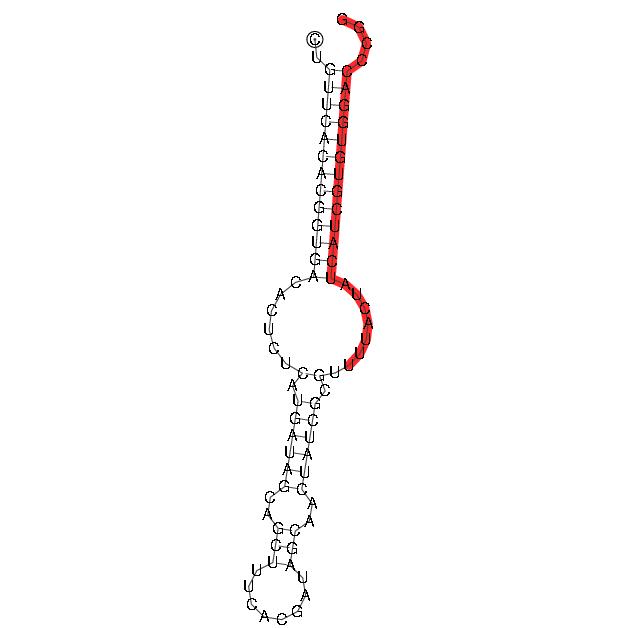

Supplement: Supplementary file 1 [file biology-13-00941-s001.zip › Data S2. Structures of novel miRNAs under treatment 1/novel_206_novel_206.jpg]

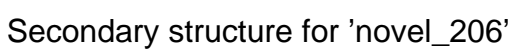

Supplement: Supplementary file 1 [file biology-13-00941-s001.zip › Data S2. Structures of novel miRNAs under treatment 1/novel_206_novel_206.pdf]

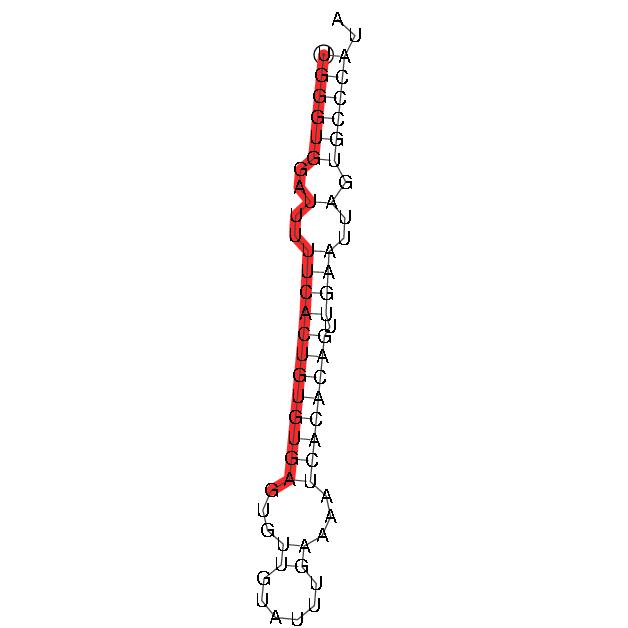

Supplement: Supplementary file 1 [file biology-13-00941-s001.zip › Data S2. Structures of novel miRNAs under treatment 1/novel_209_novel_209.jpg]

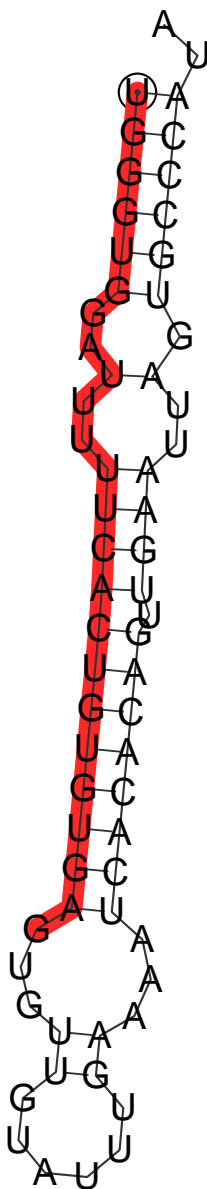

Secondary structure for 'novel\_209'

Supplement: Supplementary file 1 [file biology-13-00941-s001.zip › Data S2. Structures of novel miRNAs under treatment 1/novel_209_novel_209.pdf]

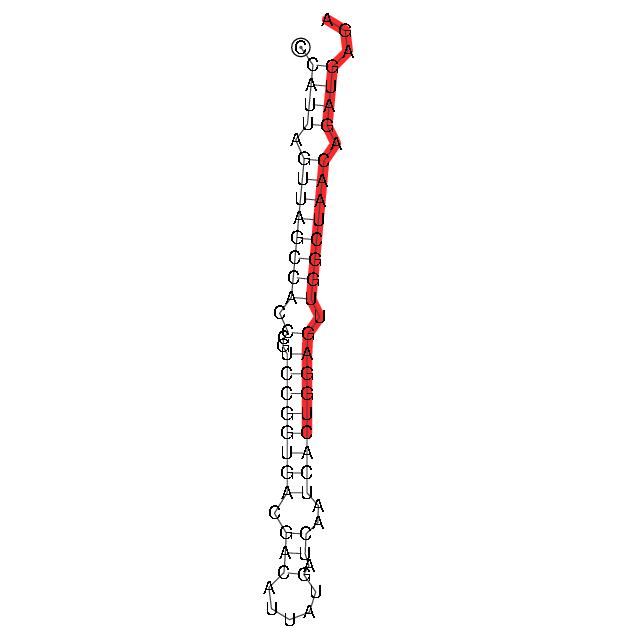

Supplement: Supplementary file 1 [file biology-13-00941-s001.zip › Data S2. Structures of novel miRNAs under treatment 1/novel_210_novel_210.jpg]

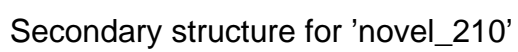

Supplement: Supplementary file 1 [file biology-13-00941-s001.zip › Data S2. Structures of novel miRNAs under treatment 1/novel_210_novel_210.pdf]

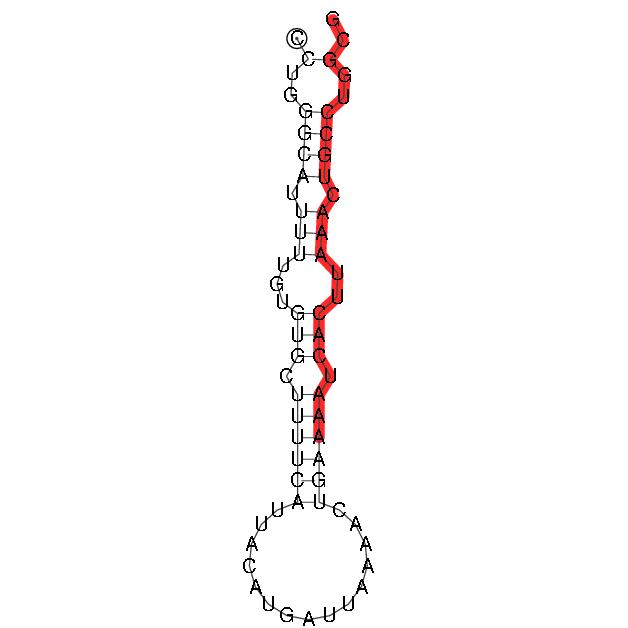

Supplement: Supplementary file 1 [file biology-13-00941-s001.zip › Data S2. Structures of novel miRNAs under treatment 1/novel_211_novel_211.jpg]

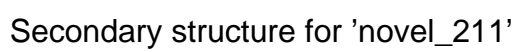

Supplement: Supplementary file 1 [file biology-13-00941-s001.zip › Data S2. Structures of novel miRNAs under treatment 1/novel_211_novel_211.pdf]

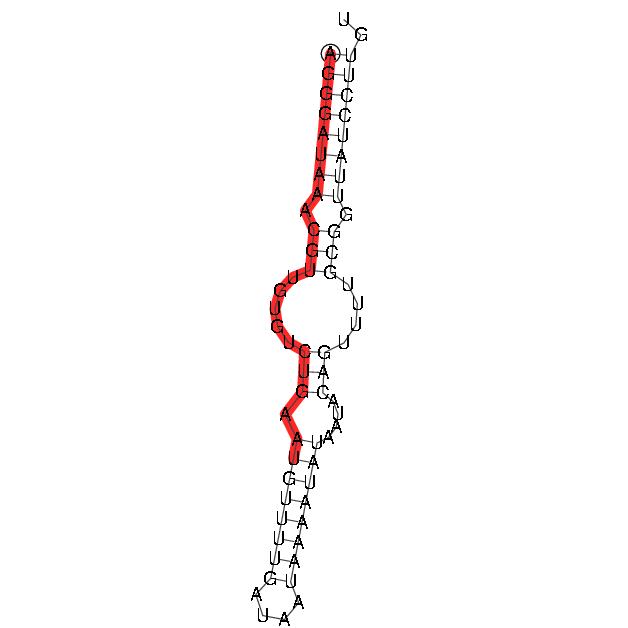

Supplement: Supplementary file 1 [file biology-13-00941-s001.zip › Data S2. Structures of novel miRNAs under treatment 1/novel_213_novel_213.jpg]

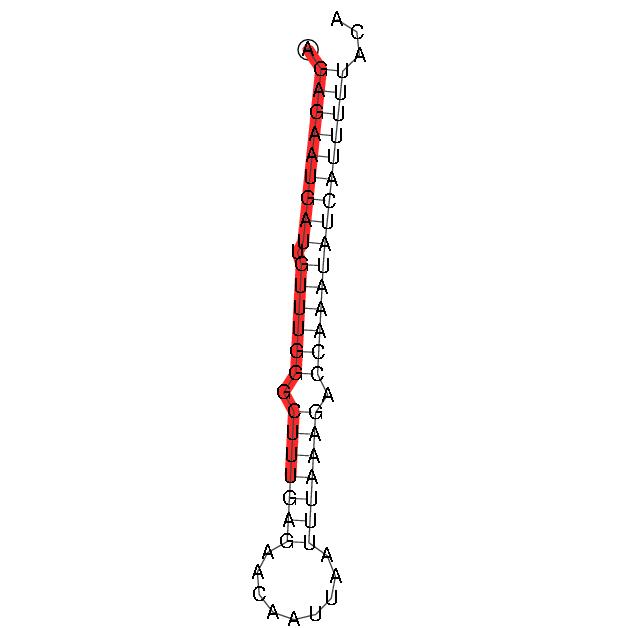

Supplement: Supplementary file 1 [file biology-13-00941-s001.zip › Data S2. Structures of novel miRNAs under treatment 1/novel_214_novel_214.jpg]

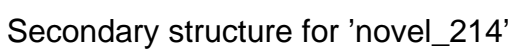

Supplement: Supplementary file 1 [file biology-13-00941-s001.zip › Data S2. Structures of novel miRNAs under treatment 1/novel_214_novel_214.pdf]

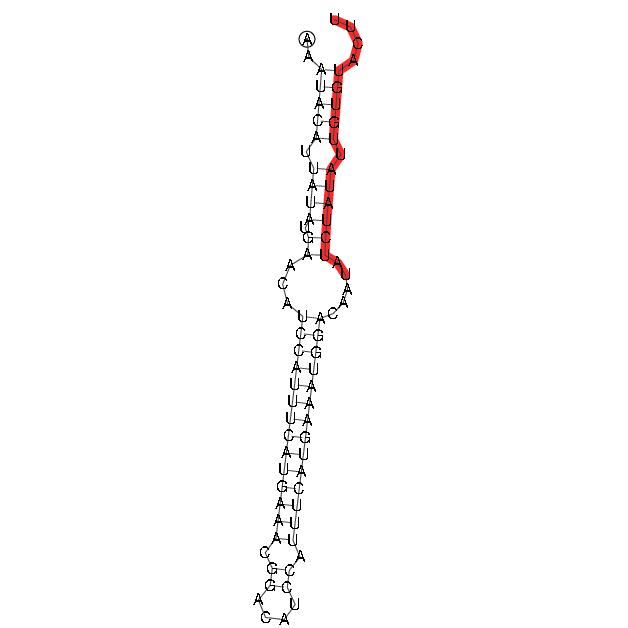

Supplement: Supplementary file 1 [file biology-13-00941-s001.zip › Data S2. Structures of novel miRNAs under treatment 1/novel_216_novel_216.jpg]

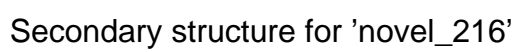

Supplement: Supplementary file 1 [file biology-13-00941-s001.zip › Data S2. Structures of novel miRNAs under treatment 1/novel_216_novel_216.pdf]

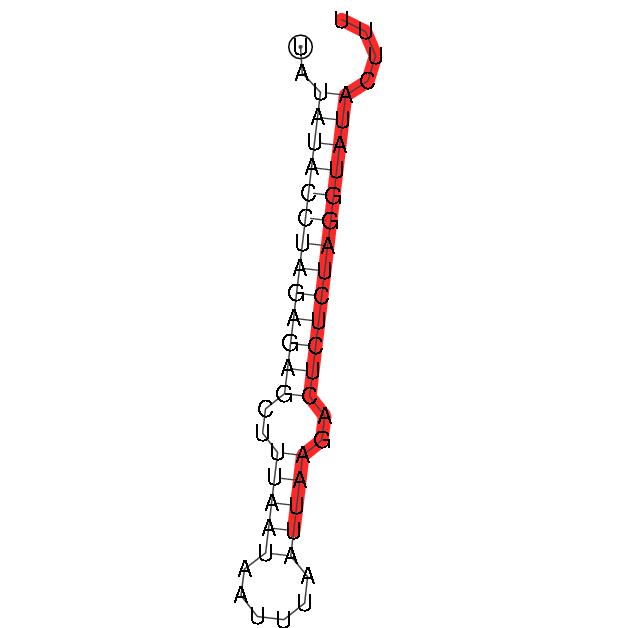

Supplement: Supplementary file 1 [file biology-13-00941-s001.zip › Data S2. Structures of novel miRNAs under treatment 1/novel_217_novel_217.jpg]

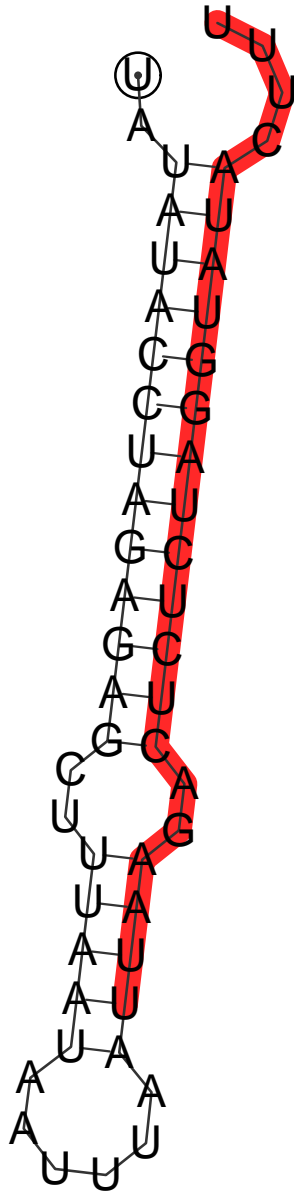

Secondary structure for 'novel\_217'

Supplement: Supplementary file 1 [file biology-13-00941-s001.zip › Data S2. Structures of novel miRNAs under treatment 1/novel_217_novel_217.pdf]

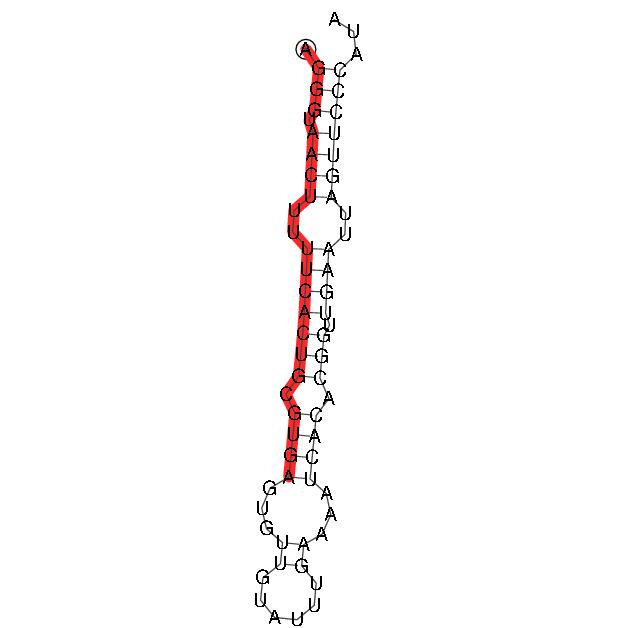

Supplement: Supplementary file 1 [file biology-13-00941-s001.zip › Data S2. Structures of novel miRNAs under treatment 1/novel_218_novel_218.jpg]

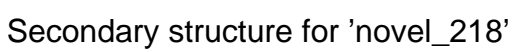

Supplement: Supplementary file 1 [file biology-13-00941-s001.zip › Data S2. Structures of novel miRNAs under treatment 1/novel_218_novel_218.pdf]

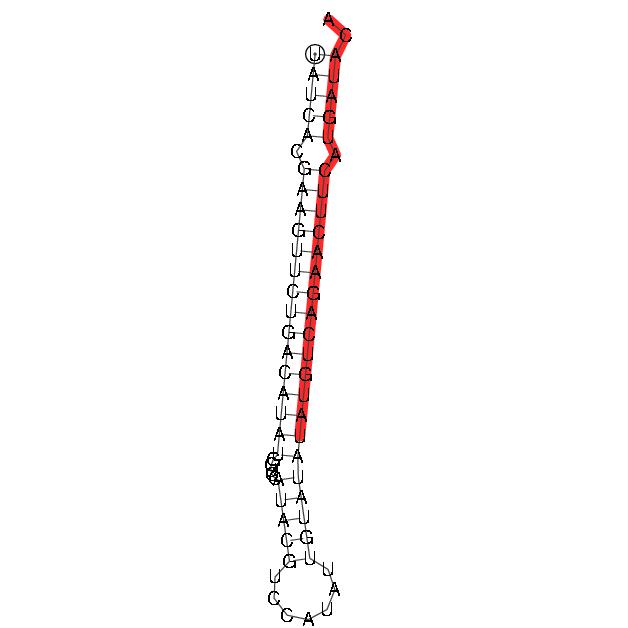

Supplement: Supplementary file 1 [file biology-13-00941-s001.zip › Data S2. Structures of novel miRNAs under treatment 1/novel_219_novel_219.jpg]

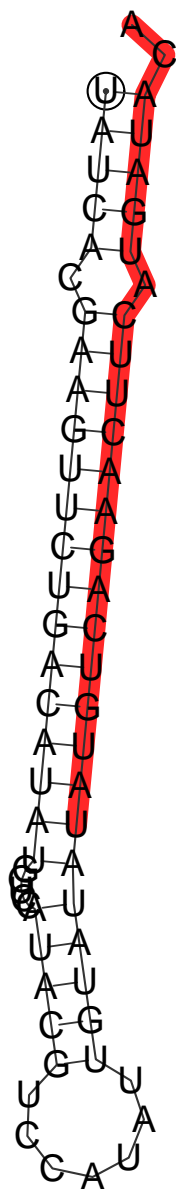

Secondary structure for 'novel\_219'

Supplement: Supplementary file 1 [file biology-13-00941-s001.zip › Data S2. Structures of novel miRNAs under treatment 1/novel_219_novel_219.pdf]

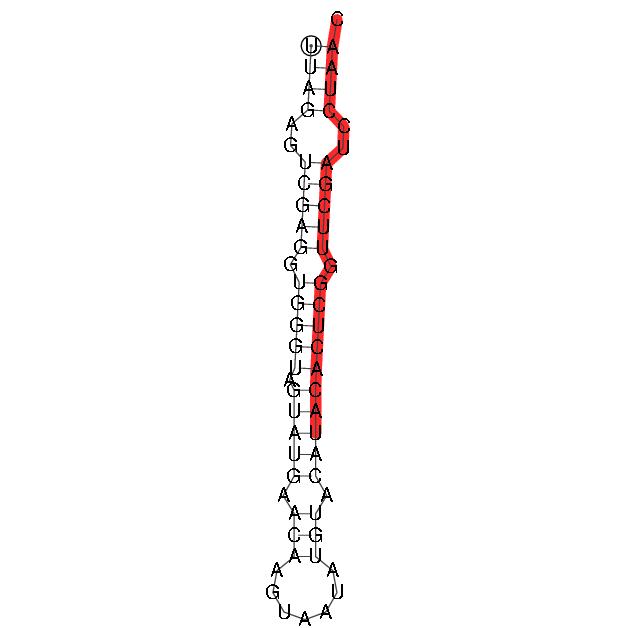

Supplement: Supplementary file 1 [file biology-13-00941-s001.zip › Data S2. Structures of novel miRNAs under treatment 1/novel_21_novel_21.jpg]

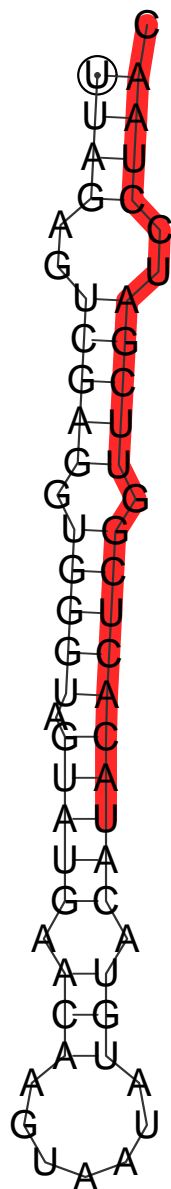

Secondary structure for 'novel\_21'

Supplement: Supplementary file 1 [file biology-13-00941-s001.zip › Data S2. Structures of novel miRNAs under treatment 1/novel_21_novel_21.pdf]

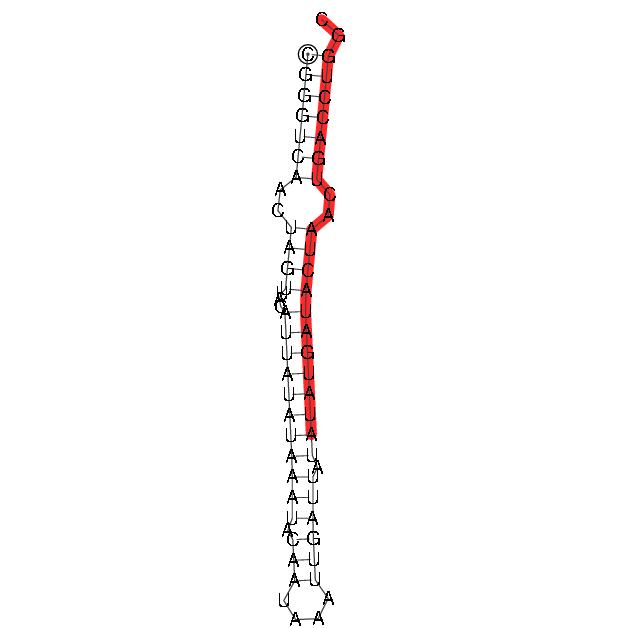

Supplement: Supplementary file 1 [file biology-13-00941-s001.zip › Data S2. Structures of novel miRNAs under treatment 1/novel_220_novel_220.jpg]

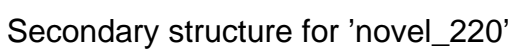

Supplement: Supplementary file 1 [file biology-13-00941-s001.zip › Data S2. Structures of novel miRNAs under treatment 1/novel_220_novel_220.pdf]

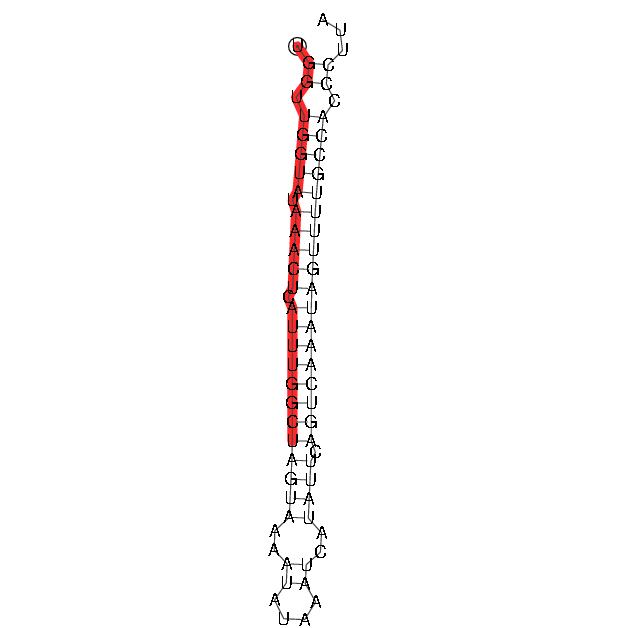

Supplement: Supplementary file 1 [file biology-13-00941-s001.zip › Data S2. Structures of novel miRNAs under treatment 1/novel_224_novel_224.jpg]

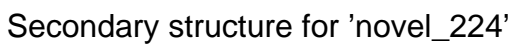

Supplement: Supplementary file 1 [file biology-13-00941-s001.zip › Data S2. Structures of novel miRNAs under treatment 1/novel_224_novel_224.pdf]

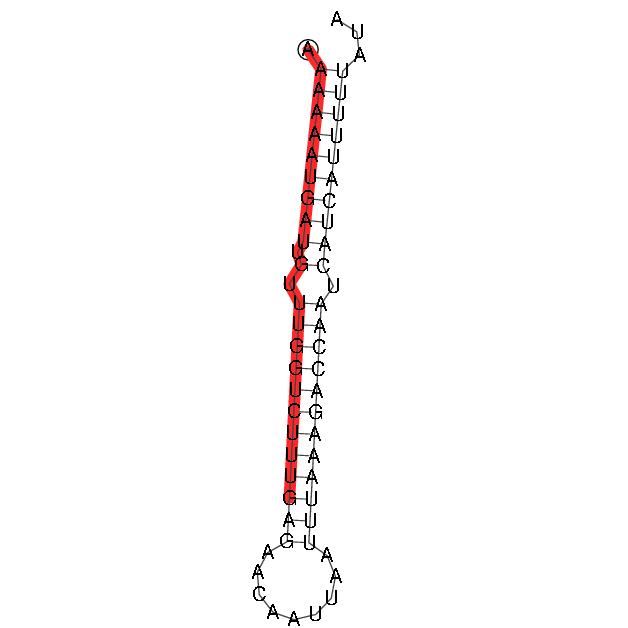

Supplement: Supplementary file 1 [file biology-13-00941-s001.zip › Data S2. Structures of novel miRNAs under treatment 1/novel_226_novel_226.jpg]

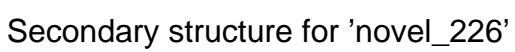

Supplement: Supplementary file 1 [file biology-13-00941-s001.zip › Data S2. Structures of novel miRNAs under treatment 1/novel_226_novel_226.pdf]

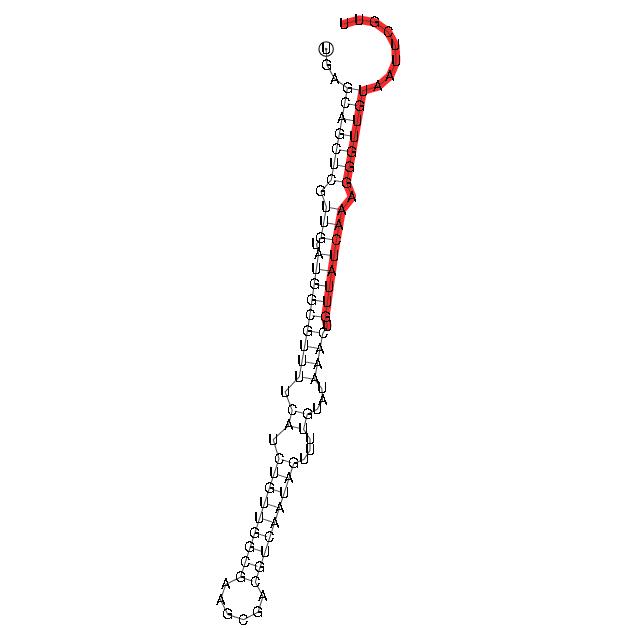

Supplement: Supplementary file 1 [file biology-13-00941-s001.zip › Data S2. Structures of novel miRNAs under treatment 1/novel_227_novel_227.jpg]

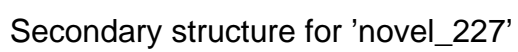

Supplement: Supplementary file 1 [file biology-13-00941-s001.zip › Data S2. Structures of novel miRNAs under treatment 1/novel_227_novel_227.pdf]

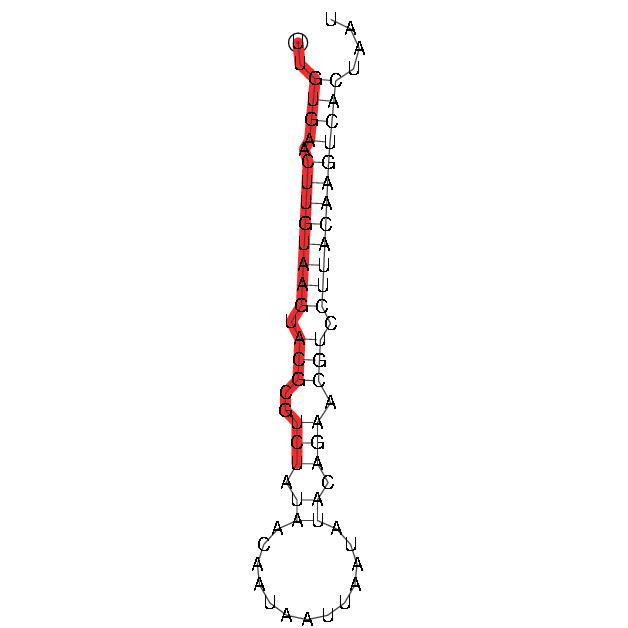

Supplement: Supplementary file 1 [file biology-13-00941-s001.zip › Data S2. Structures of novel miRNAs under treatment 1/novel_228_novel_228.jpg]

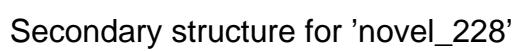

Supplement: Supplementary file 1 [file biology-13-00941-s001.zip › Data S2. Structures of novel miRNAs under treatment 1/novel_228_novel_228.pdf]

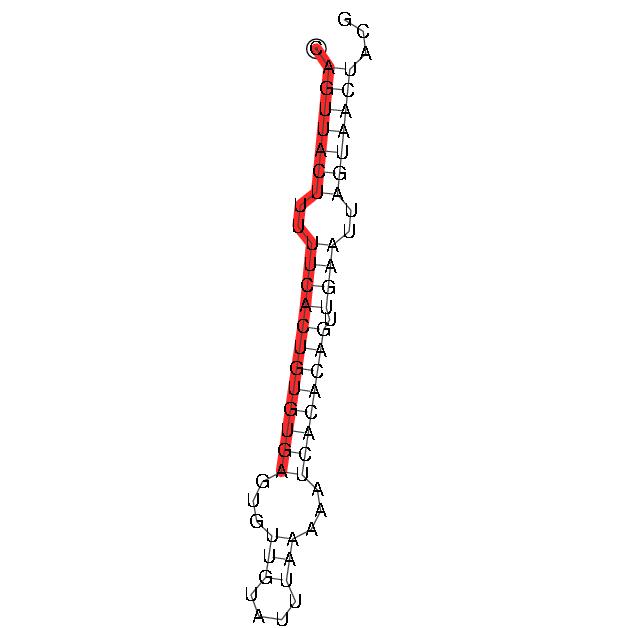

Supplement: Supplementary file 1 [file biology-13-00941-s001.zip › Data S2. Structures of novel miRNAs under treatment 1/novel_229_novel_229.jpg]

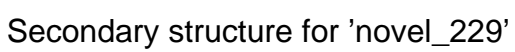

Supplement: Supplementary file 1 [file biology-13-00941-s001.zip › Data S2. Structures of novel miRNAs under treatment 1/novel_229_novel_229.pdf]

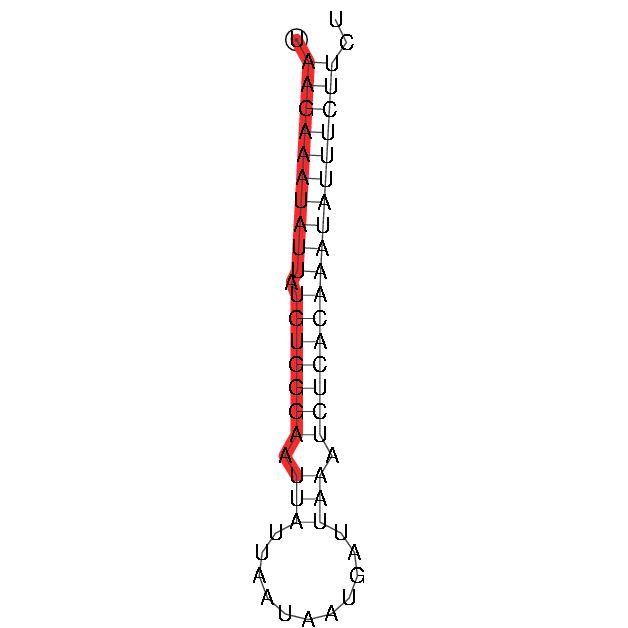

Supplement: Supplementary file 1 [file biology-13-00941-s001.zip › Data S2. Structures of novel miRNAs under treatment 1/novel_231_novel_231.jpg]

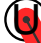

Secondary structure for 'novel\_231'

Supplement: Supplementary file 1 [file biology-13-00941-s001.zip › Data S2. Structures of novel miRNAs under treatment 1/novel_231_novel_231.pdf]

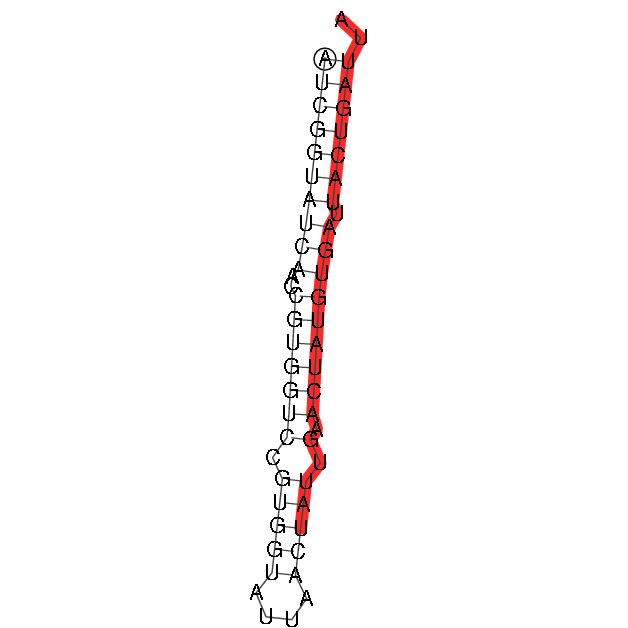

Supplement: Supplementary file 1 [file biology-13-00941-s001.zip › Data S2. Structures of novel miRNAs under treatment 1/novel_232_novel_232.jpg]

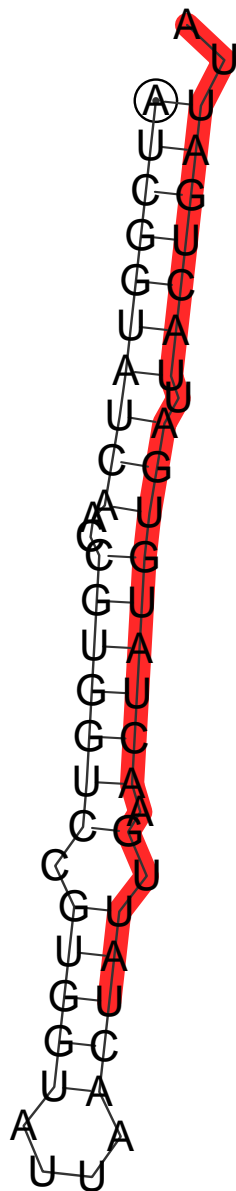

Secondary structure for 'novel\_232'

Supplement: Supplementary file 1 [file biology-13-00941-s001.zip › Data S2. Structures of novel miRNAs under treatment 1/novel_232_novel_232.pdf]

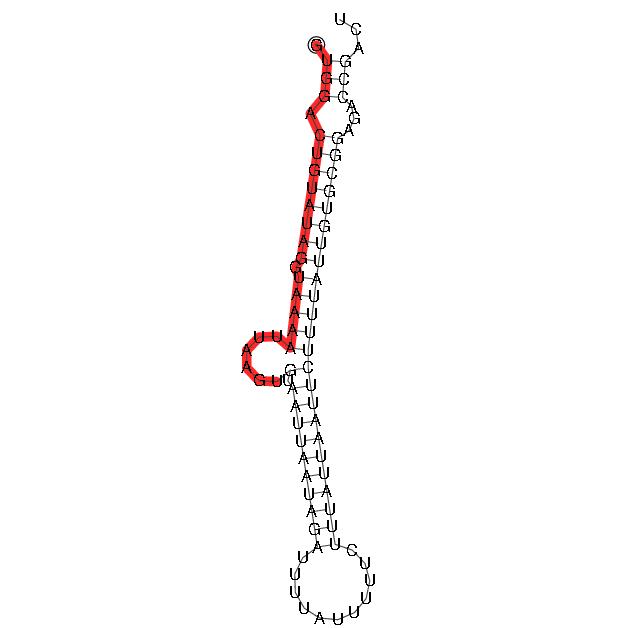

Supplement: Supplementary file 1 [file biology-13-00941-s001.zip › Data S2. Structures of novel miRNAs under treatment 1/novel_235_novel_235.jpg]

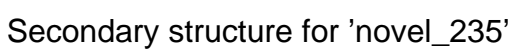

Supplement: Supplementary file 1 [file biology-13-00941-s001.zip › Data S2. Structures of novel miRNAs under treatment 1/novel_235_novel_235.pdf]

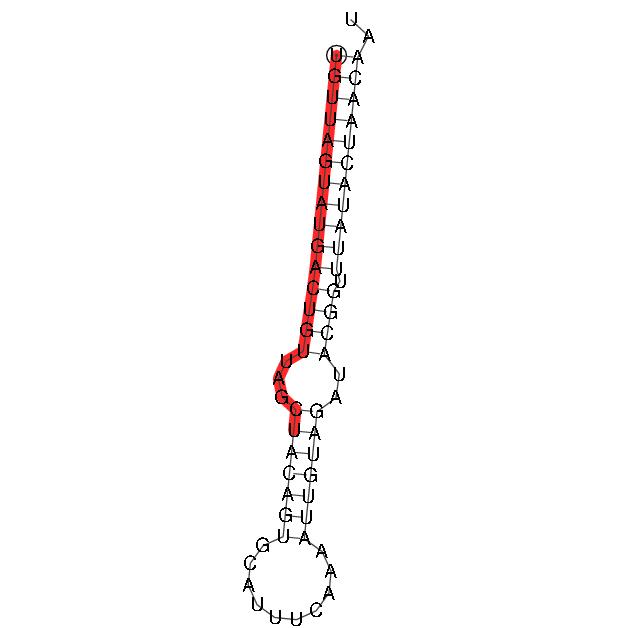

Supplement: Supplementary file 1 [file biology-13-00941-s001.zip › Data S2. Structures of novel miRNAs under treatment 1/novel_237_novel_237.jpg]

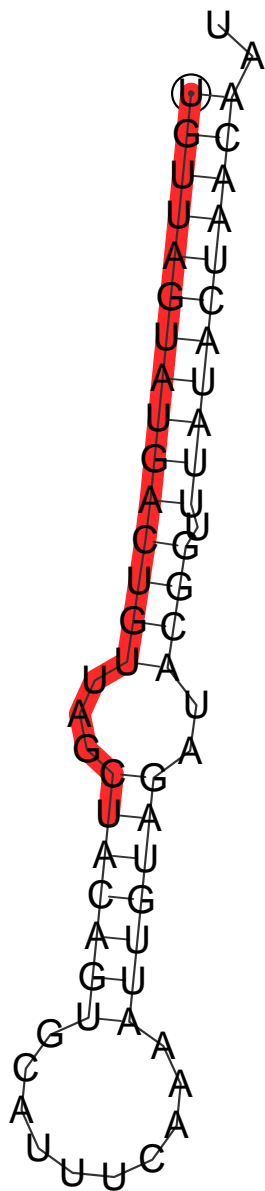

Secondary structure for 'novel\_237'

Supplement: Supplementary file 1 [file biology-13-00941-s001.zip › Data S2. Structures of novel miRNAs under treatment 1/novel_237_novel_237.pdf]

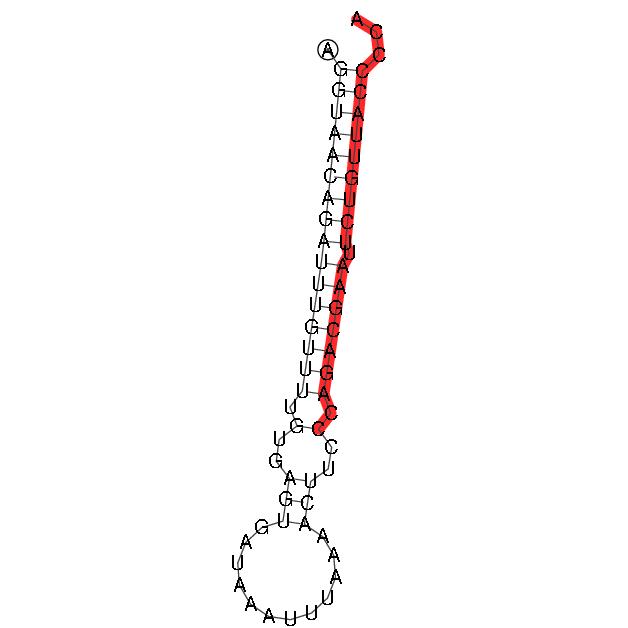

Supplement: Supplementary file 1 [file biology-13-00941-s001.zip › Data S2. Structures of novel miRNAs under treatment 1/novel_25_novel_25.jpg]

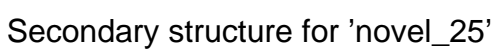

Supplement: Supplementary file 1 [file biology-13-00941-s001.zip › Data S2. Structures of novel miRNAs under treatment 1/novel_25_novel_25.pdf]

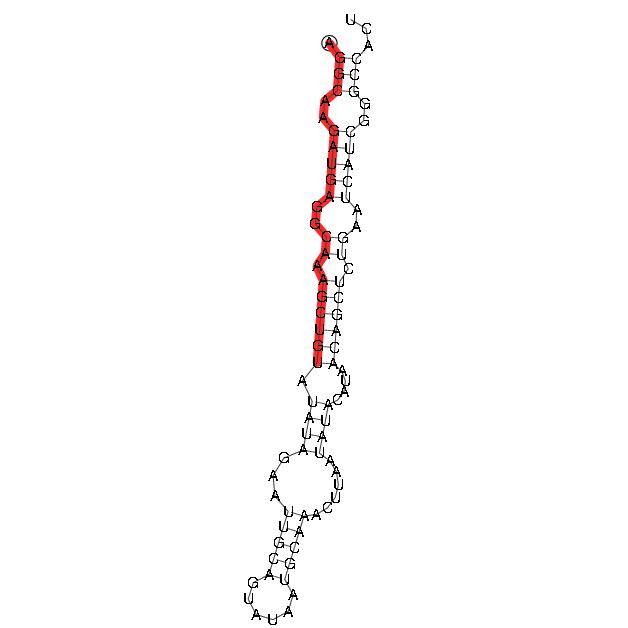

Supplement: Supplementary file 1 [file biology-13-00941-s001.zip › Data S2. Structures of novel miRNAs under treatment 1/novel_28_novel_28.jpg]

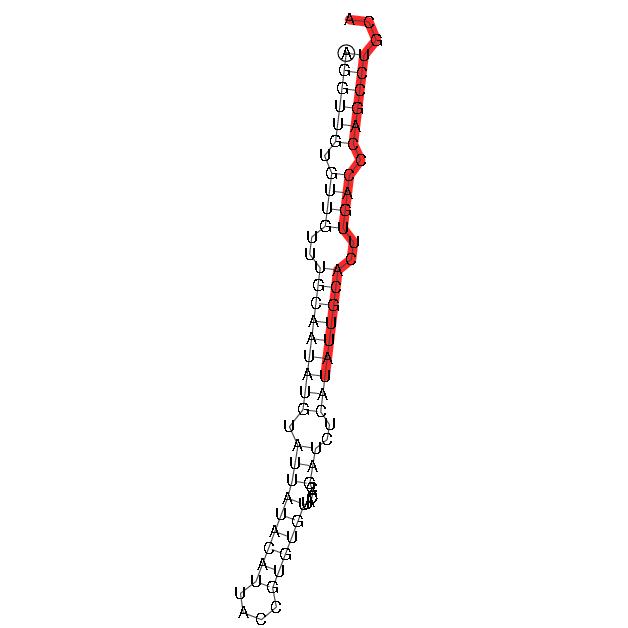

Supplement: Supplementary file 1 [file biology-13-00941-s001.zip › Data S2. Structures of novel miRNAs under treatment 1/novel_30_novel_30.jpg]

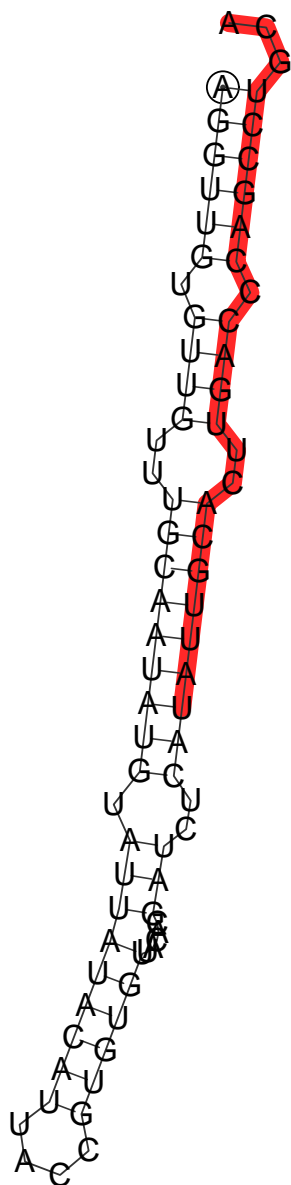

Secondary structure for 'novel\_30'

Supplement: Supplementary file 1 [file biology-13-00941-s001.zip › Data S2. Structures of novel miRNAs under treatment 1/novel_30_novel_30.pdf]

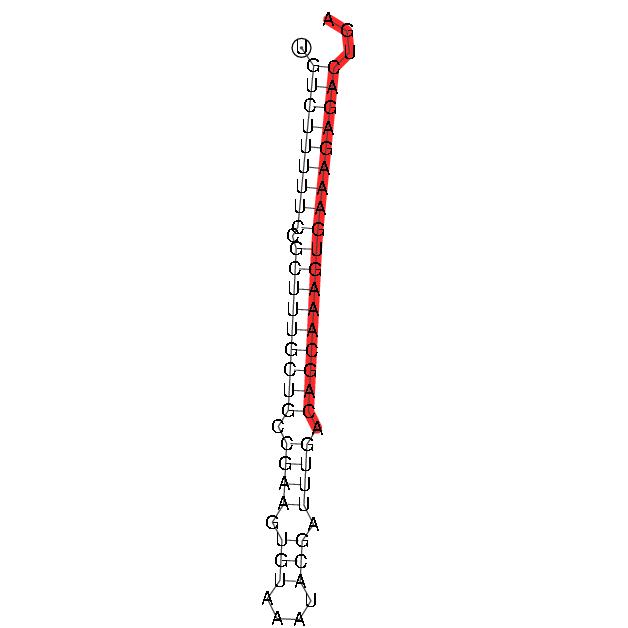

Supplement: Supplementary file 1 [file biology-13-00941-s001.zip › Data S2. Structures of novel miRNAs under treatment 1/novel_36_novel_36.jpg]

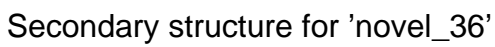

Supplement: Supplementary file 1 [file biology-13-00941-s001.zip › Data S2. Structures of novel miRNAs under treatment 1/novel_36_novel_36.pdf]

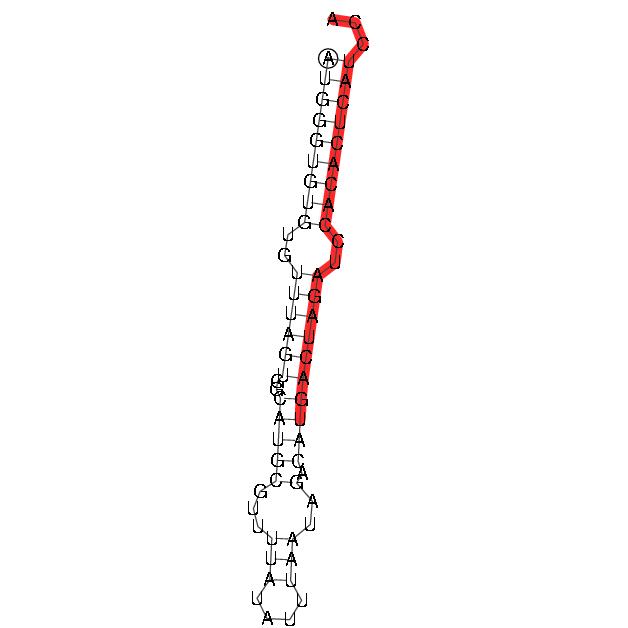

Supplement: Supplementary file 1 [file biology-13-00941-s001.zip › Data S2. Structures of novel miRNAs under treatment 1/novel_3_novel_3.jpg]

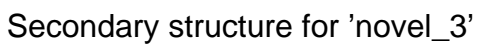

Supplement: Supplementary file 1 [file biology-13-00941-s001.zip › Data S2. Structures of novel miRNAs under treatment 1/novel_3_novel_3.pdf]

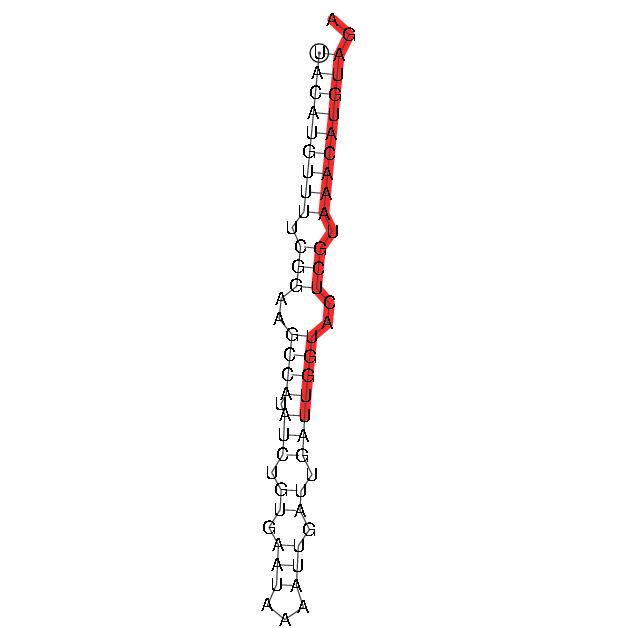

Supplement: Supplementary file 1 [file biology-13-00941-s001.zip › Data S2. Structures of novel miRNAs under treatment 1/novel_54_novel_54.jpg]

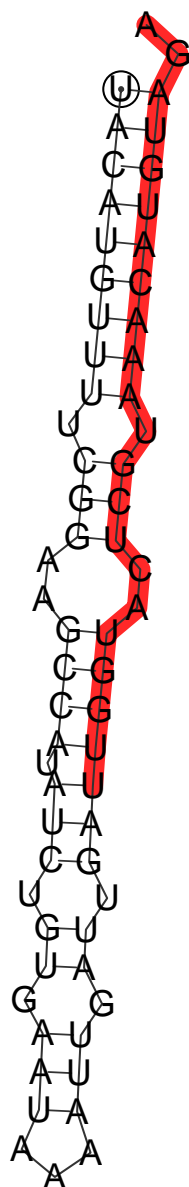

Supplement: Supplementary file 1 [file biology-13-00941-s001.zip › Data S2. Structures of novel miRNAs under treatment 1/novel_54_novel_54.pdf]

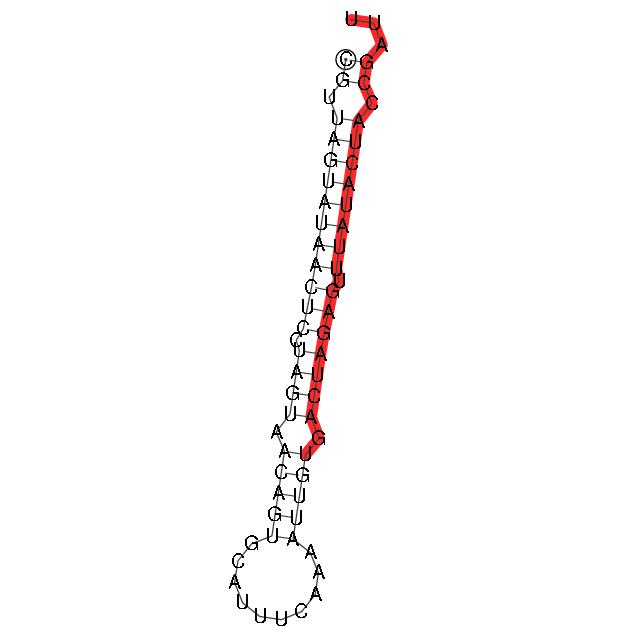

Supplement: Supplementary file 1 [file biology-13-00941-s001.zip › Data S2. Structures of novel miRNAs under treatment 1/novel_56_novel_56.jpg]

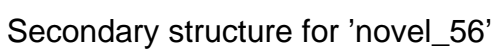

Supplement: Supplementary file 1 [file biology-13-00941-s001.zip › Data S2. Structures of novel miRNAs under treatment 1/novel_56_novel_56.pdf]

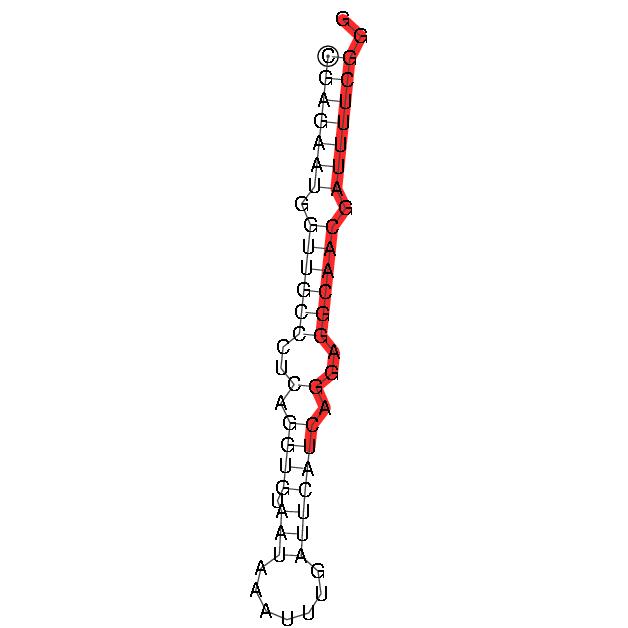

Supplement: Supplementary file 1 [file biology-13-00941-s001.zip › Data S2. Structures of novel miRNAs under treatment 1/novel_60_novel_60.jpg]

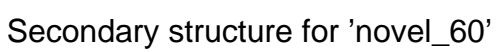

Supplement: Supplementary file 1 [file biology-13-00941-s001.zip › Data S2. Structures of novel miRNAs under treatment 1/novel_60_novel_60.pdf]

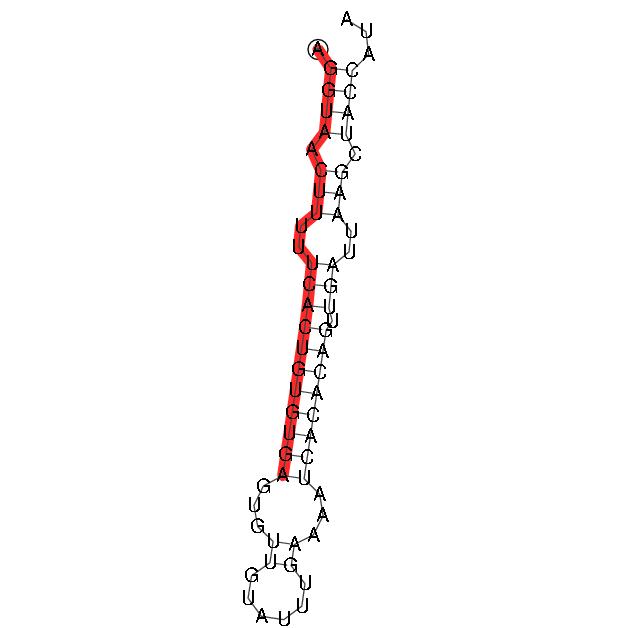

Supplement: Supplementary file 1 [file biology-13-00941-s001.zip › Data S2. Structures of novel miRNAs under treatment 1/novel_61_novel_61.jpg]

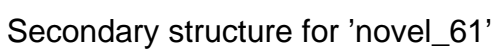

Supplement: Supplementary file 1 [file biology-13-00941-s001.zip › Data S2. Structures of novel miRNAs under treatment 1/novel_61_novel_61.pdf]

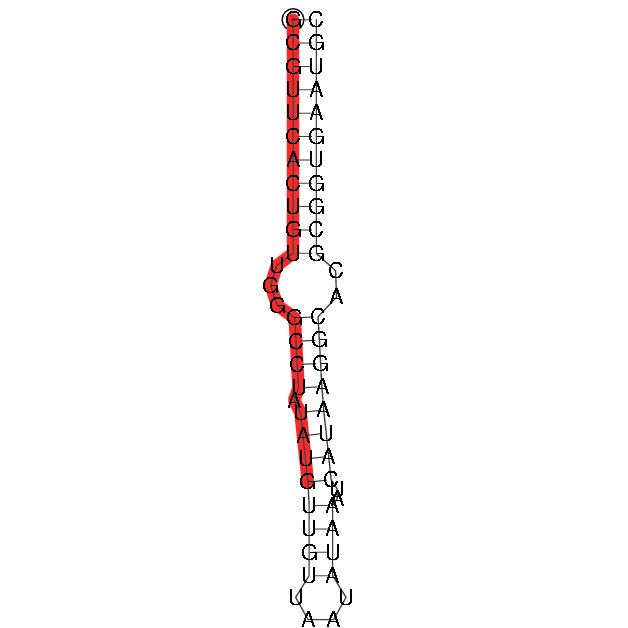

Supplement: Supplementary file 1 [file biology-13-00941-s001.zip › Data S2. Structures of novel miRNAs under treatment 1/novel_65_novel_65.jpg]

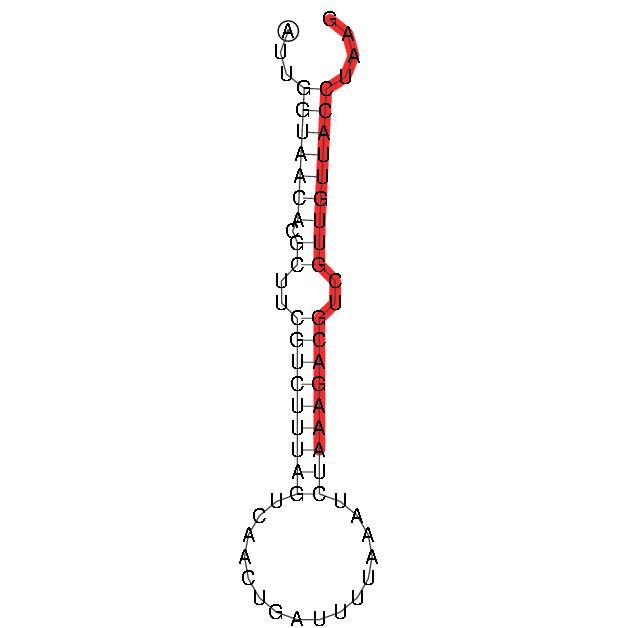

Supplement: Supplementary file 1 [file biology-13-00941-s001.zip › Data S2. Structures of novel miRNAs under treatment 1/novel_69_novel_69.jpg]

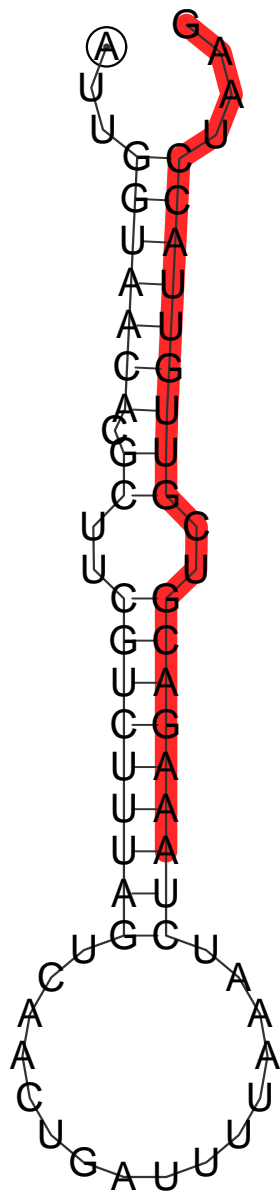

Secondary structure for 'novel\_69'

Supplement: Supplementary file 1 [file biology-13-00941-s001.zip › Data S2. Structures of novel miRNAs under treatment 1/novel_69_novel_69.pdf]

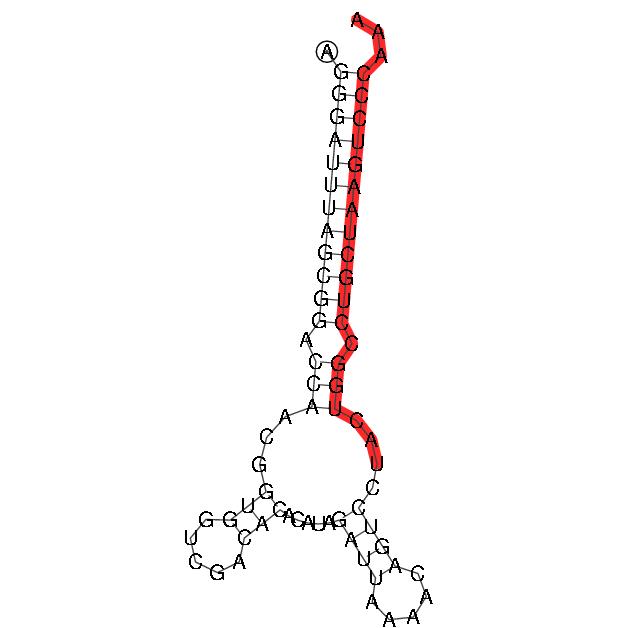

Supplement: Supplementary file 1 [file biology-13-00941-s001.zip › Data S2. Structures of novel miRNAs under treatment 1/novel_71_novel_71.jpg]

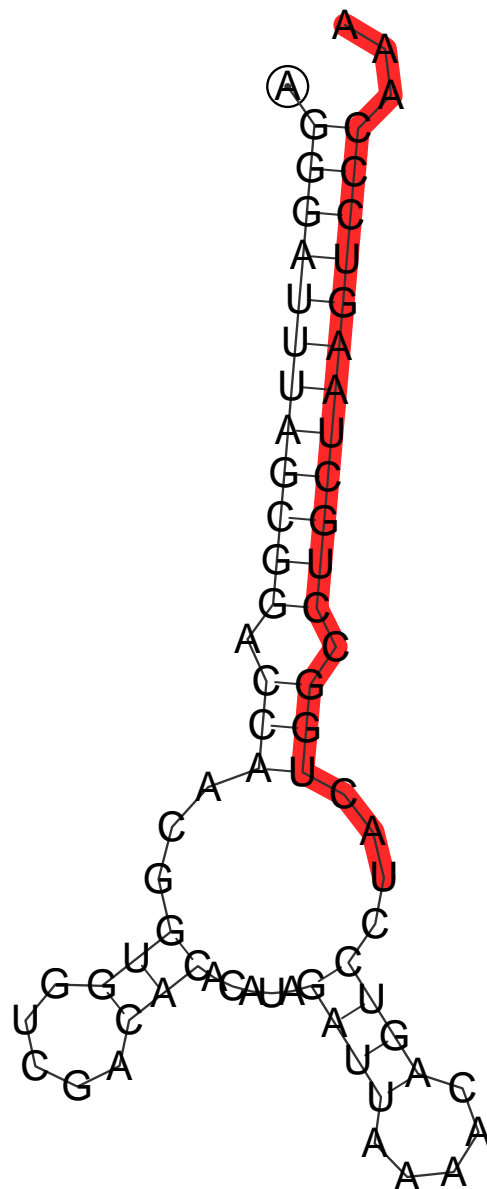

Supplement: Supplementary file 1 [file biology-13-00941-s001.zip › Data S2. Structures of novel miRNAs under treatment 1/novel_71_novel_71.pdf]

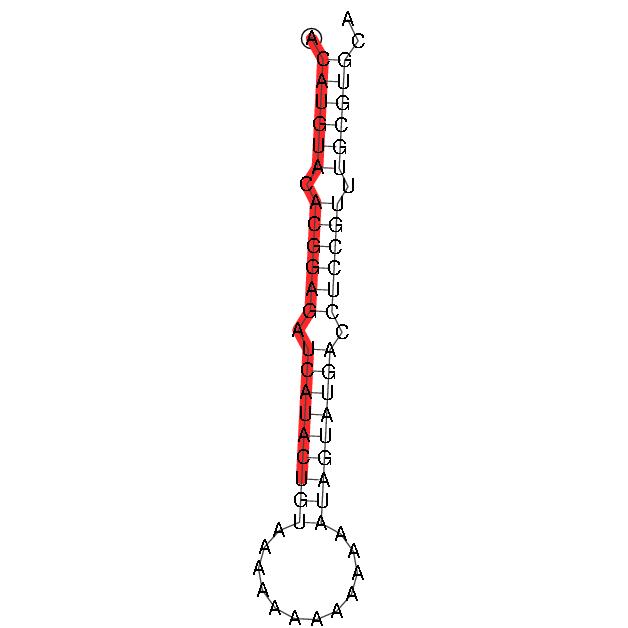

Supplement: Supplementary file 1 [file biology-13-00941-s001.zip › Data S2. Structures of novel miRNAs under treatment 1/novel_72_novel_72.jpg]

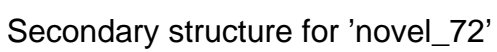

Supplement: Supplementary file 1 [file biology-13-00941-s001.zip › Data S2. Structures of novel miRNAs under treatment 1/novel_72_novel_72.pdf]

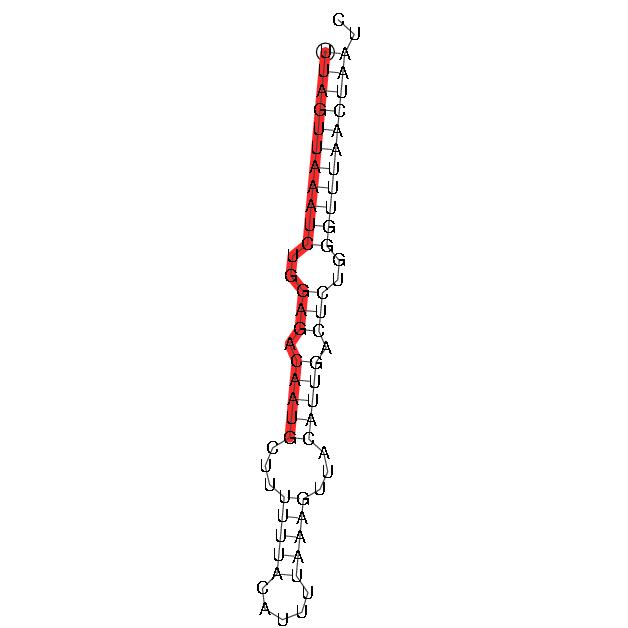

Supplement: Supplementary file 1 [file biology-13-00941-s001.zip › Data S2. Structures of novel miRNAs under treatment 1/novel_75_novel_75.jpg]

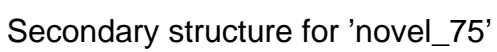

Supplement: Supplementary file 1 [file biology-13-00941-s001.zip › Data S2. Structures of novel miRNAs under treatment 1/novel_75_novel_75.pdf]

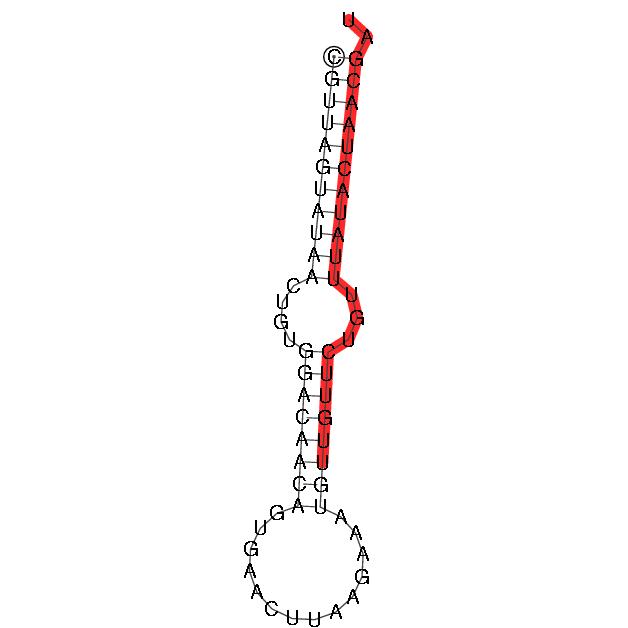

Supplement: Supplementary file 1 [file biology-13-00941-s001.zip › Data S2. Structures of novel miRNAs under treatment 1/novel_76_novel_76.jpg]
