# Supplementary figures and images for: Molecular Characterization of miRNAs in Myzus persicae Carrying Brassica Yellows Virus (part 1 of 2)
Source: Biology (Basel). 2024 Nov 18;13(11):941. doi: 10.3390/biology13110941 (PMC11591976; doi:10.3390/biology13110941)

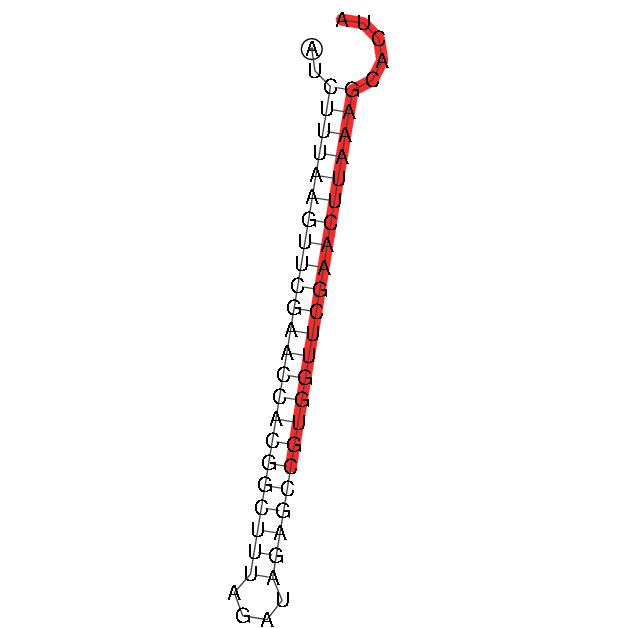

Supplement: Supplementary file 1 [file biology-13-00941-s001.zip › Data S2. Structures of novel miRNAs under treatment 1/novel_102_novel_102.jpg]

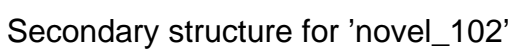

Supplement: Supplementary file 1 [file biology-13-00941-s001.zip › Data S2. Structures of novel miRNAs under treatment 1/novel_102_novel_102.pdf]

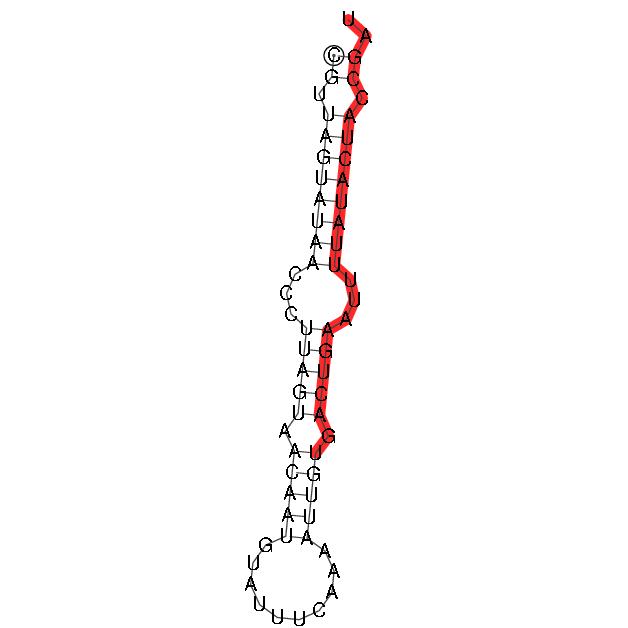

Supplement: Supplementary file 1 [file biology-13-00941-s001.zip › Data S2. Structures of novel miRNAs under treatment 1/novel_104_novel_104.jpg]

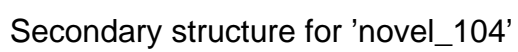

Supplement: Supplementary file 1 [file biology-13-00941-s001.zip › Data S2. Structures of novel miRNAs under treatment 1/novel_104_novel_104.pdf]

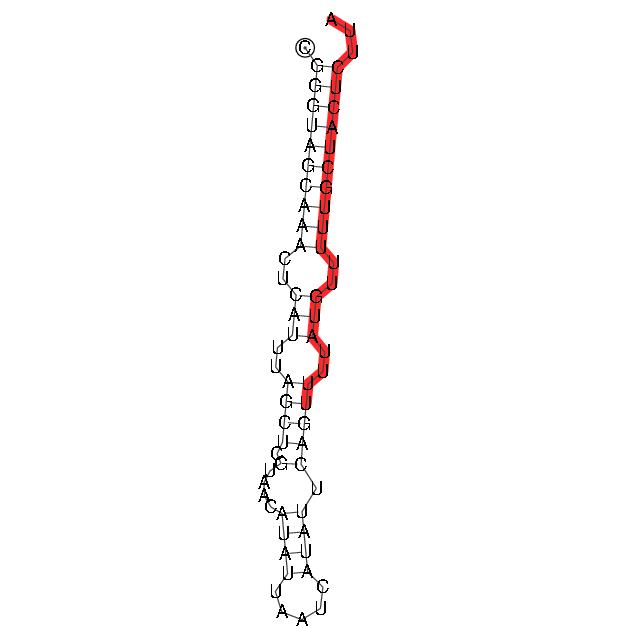

Supplement: Supplementary file 1 [file biology-13-00941-s001.zip › Data S2. Structures of novel miRNAs under treatment 1/novel_105_novel_105.jpg]

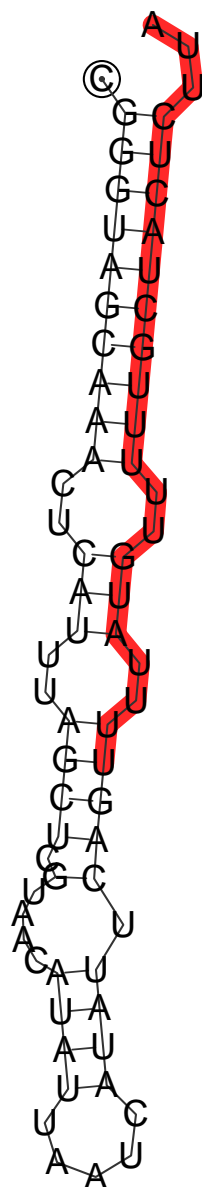

Secondary structure for 'novel\_105'

Supplement: Supplementary file 1 [file biology-13-00941-s001.zip › Data S2. Structures of novel miRNAs under treatment 1/novel_105_novel_105.pdf]

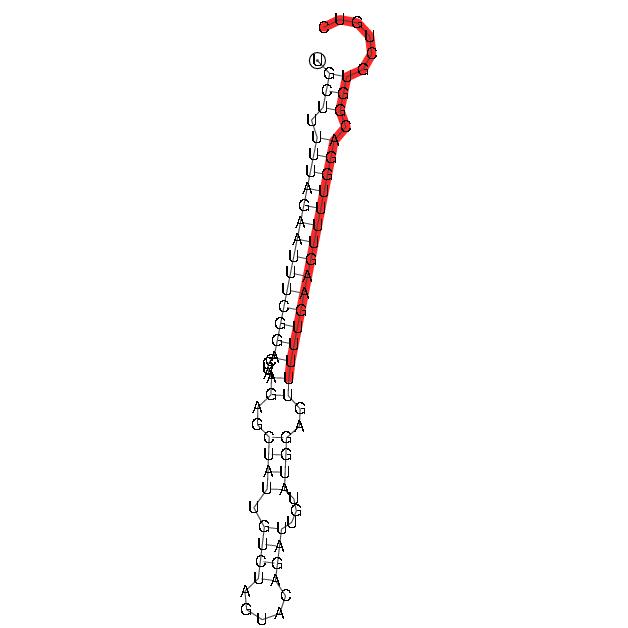

Supplement: Supplementary file 1 [file biology-13-00941-s001.zip › Data S2. Structures of novel miRNAs under treatment 1/novel_106_novel_106.jpg]

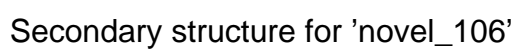

Supplement: Supplementary file 1 [file biology-13-00941-s001.zip › Data S2. Structures of novel miRNAs under treatment 1/novel_106_novel_106.pdf]

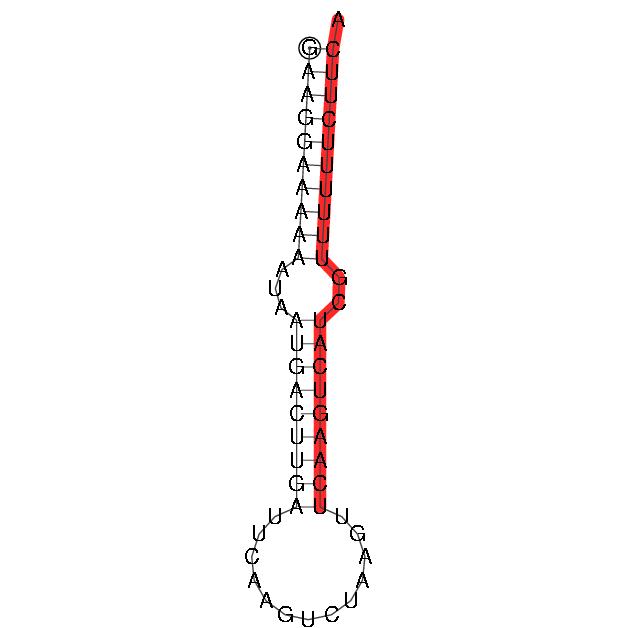

Supplement: Supplementary file 1 [file biology-13-00941-s001.zip › Data S2. Structures of novel miRNAs under treatment 1/novel_107_novel_107.jpg]

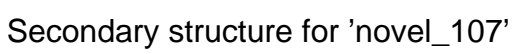

Supplement: Supplementary file 1 [file biology-13-00941-s001.zip › Data S2. Structures of novel miRNAs under treatment 1/novel_107_novel_107.pdf]

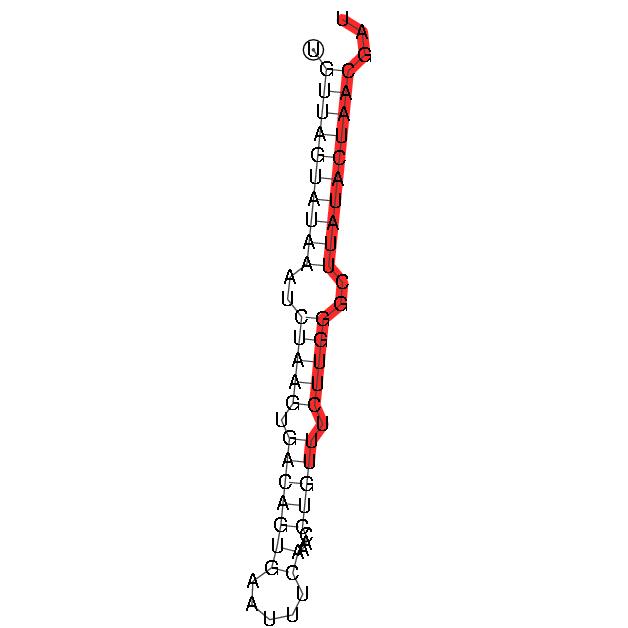

Supplement: Supplementary file 1 [file biology-13-00941-s001.zip › Data S2. Structures of novel miRNAs under treatment 1/novel_108_novel_108.jpg]

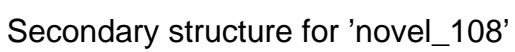

Supplement: Supplementary file 1 [file biology-13-00941-s001.zip › Data S2. Structures of novel miRNAs under treatment 1/novel_108_novel_108.pdf]

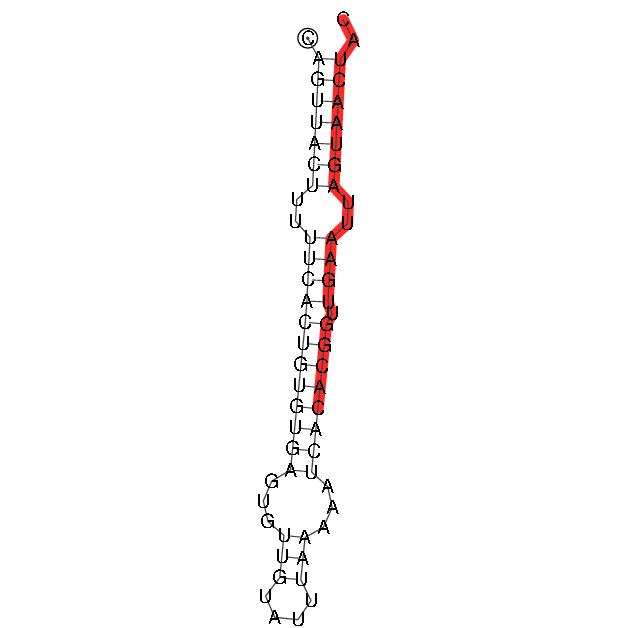

Supplement: Supplementary file 1 [file biology-13-00941-s001.zip › Data S2. Structures of novel miRNAs under treatment 1/novel_109_novel_109.jpg]

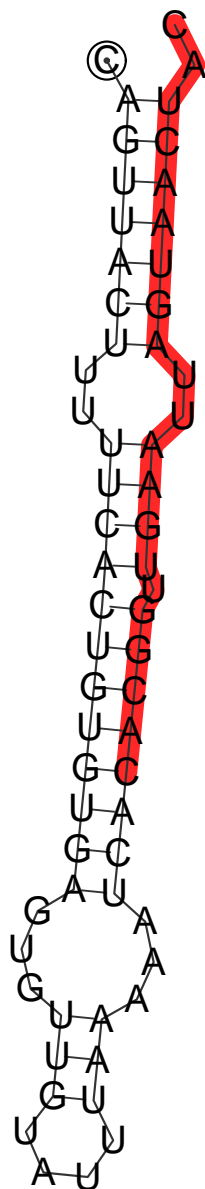

Supplement: Supplementary file 1 [file biology-13-00941-s001.zip › Data S2. Structures of novel miRNAs under treatment 1/novel_109_novel_109.pdf]

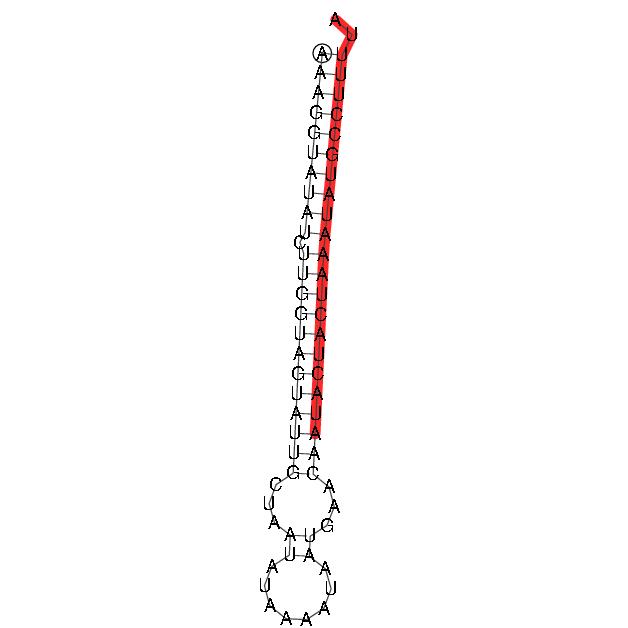

Supplement: Supplementary file 1 [file biology-13-00941-s001.zip › Data S2. Structures of novel miRNAs under treatment 1/novel_110_novel_110.jpg]

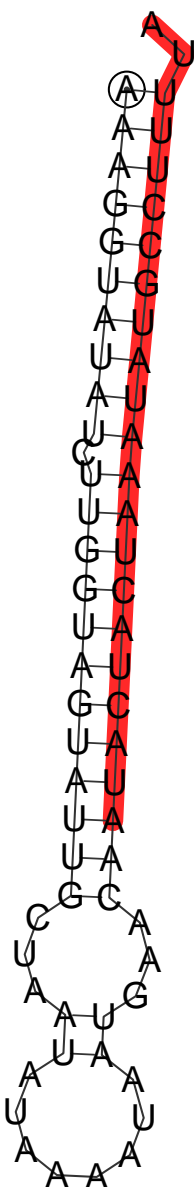

Secondary structure for 'novel\_110'

Supplement: Supplementary file 1 [file biology-13-00941-s001.zip › Data S2. Structures of novel miRNAs under treatment 1/novel_110_novel_110.pdf]

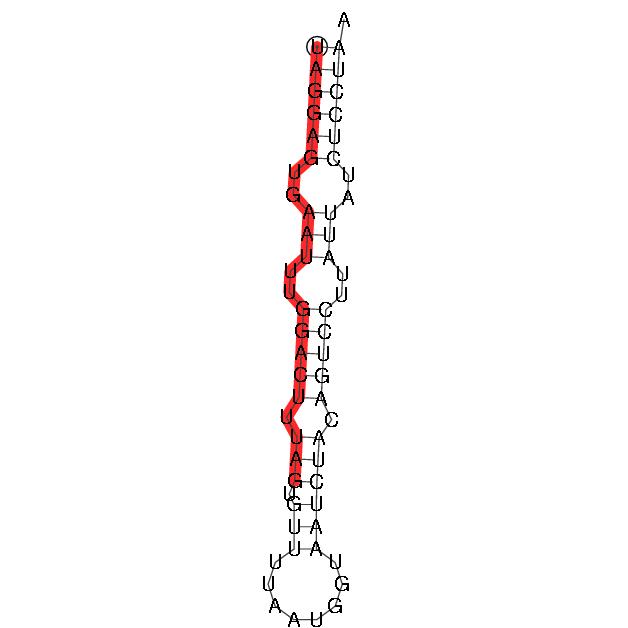

Supplement: Supplementary file 1 [file biology-13-00941-s001.zip › Data S2. Structures of novel miRNAs under treatment 1/novel_112_novel_112.jpg]

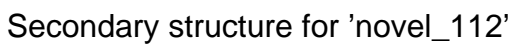

Supplement: Supplementary file 1 [file biology-13-00941-s001.zip › Data S2. Structures of novel miRNAs under treatment 1/novel_112_novel_112.pdf]

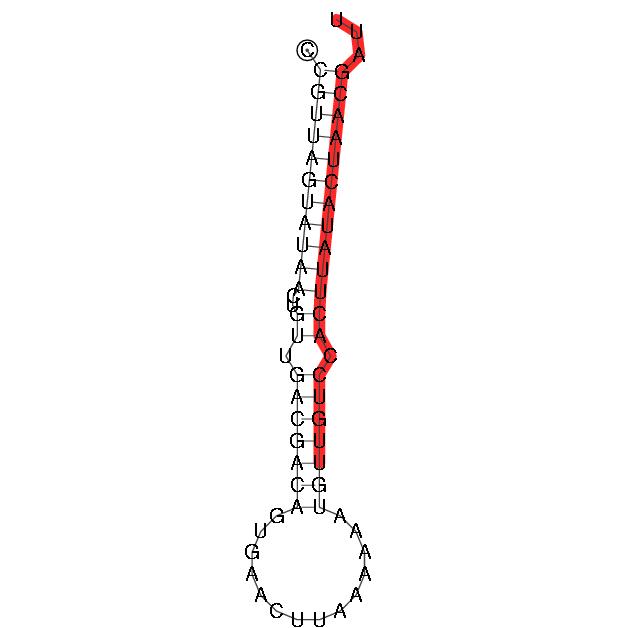

Supplement: Supplementary file 1 [file biology-13-00941-s001.zip › Data S2. Structures of novel miRNAs under treatment 1/novel_113_novel_113.jpg]

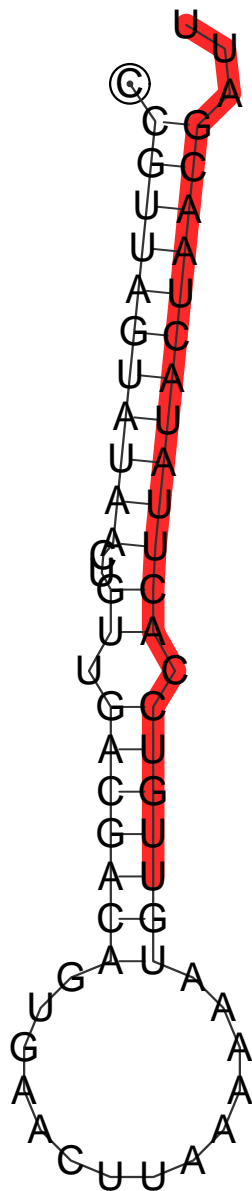

Secondary structure for 'novel\_113'

Supplement: Supplementary file 1 [file biology-13-00941-s001.zip › Data S2. Structures of novel miRNAs under treatment 1/novel_113_novel_113.pdf]

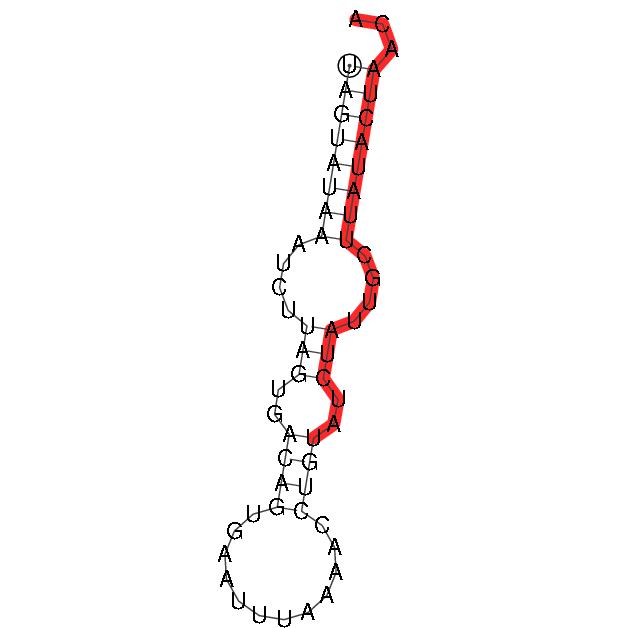

Supplement: Supplementary file 1 [file biology-13-00941-s001.zip › Data S2. Structures of novel miRNAs under treatment 1/novel_115_novel_115.jpg]

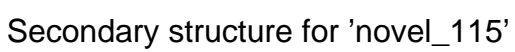

Supplement: Supplementary file 1 [file biology-13-00941-s001.zip › Data S2. Structures of novel miRNAs under treatment 1/novel_115_novel_115.pdf]

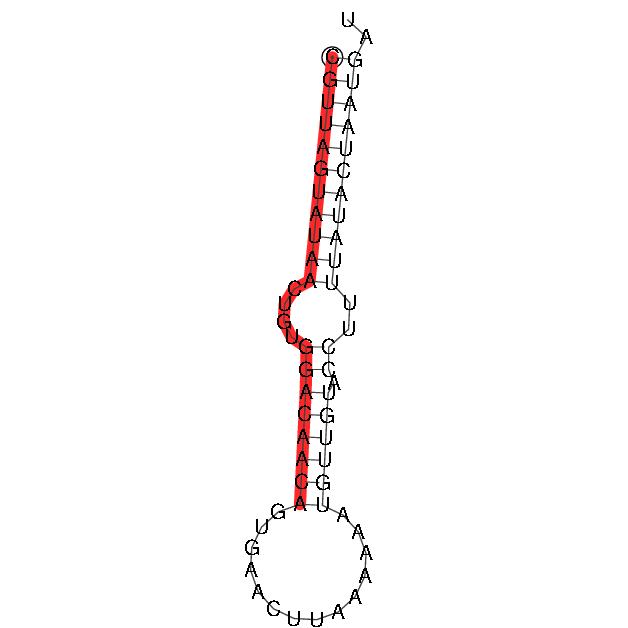

Supplement: Supplementary file 1 [file biology-13-00941-s001.zip › Data S2. Structures of novel miRNAs under treatment 1/novel_117_novel_117.jpg]

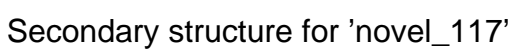

Supplement: Supplementary file 1 [file biology-13-00941-s001.zip › Data S2. Structures of novel miRNAs under treatment 1/novel_117_novel_117.pdf]

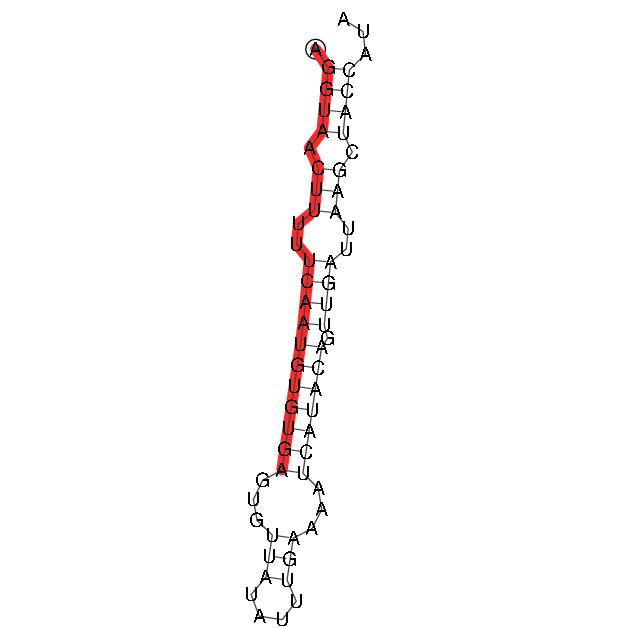

Supplement: Supplementary file 1 [file biology-13-00941-s001.zip › Data S2. Structures of novel miRNAs under treatment 1/novel_118_novel_118.jpg]

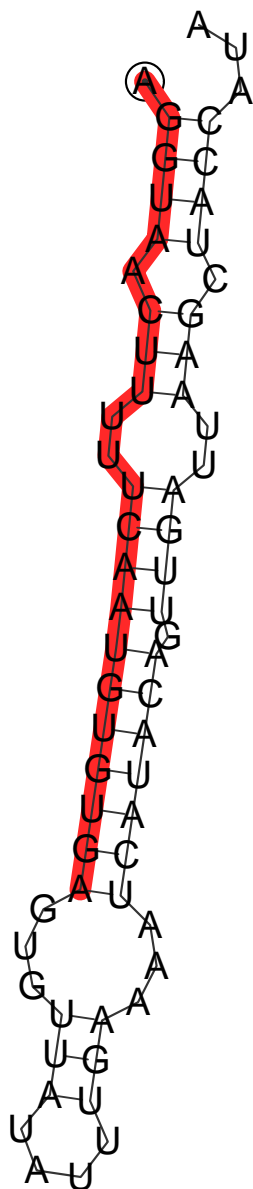

Supplement: Supplementary file 1 [file biology-13-00941-s001.zip › Data S2. Structures of novel miRNAs under treatment 1/novel_118_novel_118.pdf]

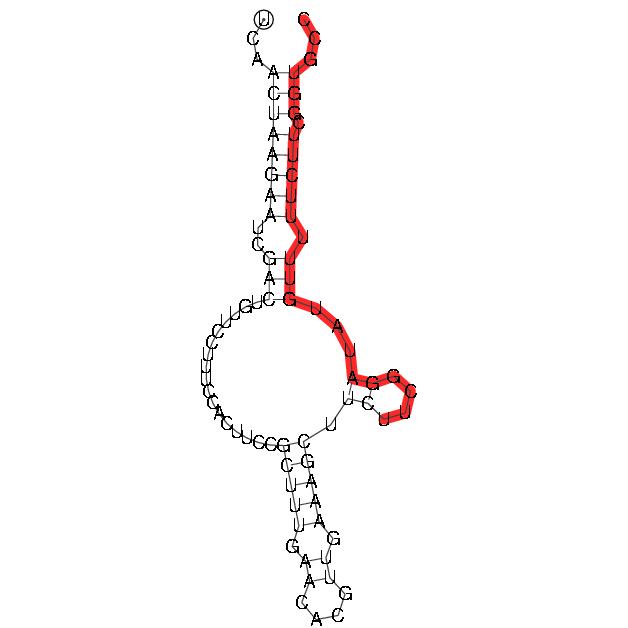

Supplement: Supplementary file 1 [file biology-13-00941-s001.zip › Data S2. Structures of novel miRNAs under treatment 1/novel_119_novel_119.jpg]

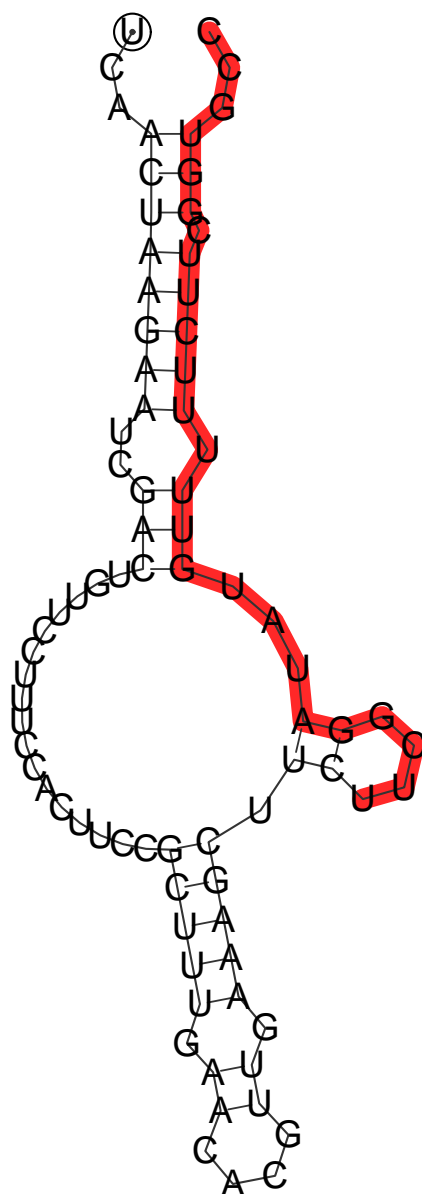

Supplement: Supplementary file 1 [file biology-13-00941-s001.zip › Data S2. Structures of novel miRNAs under treatment 1/novel_119_novel_119.pdf]

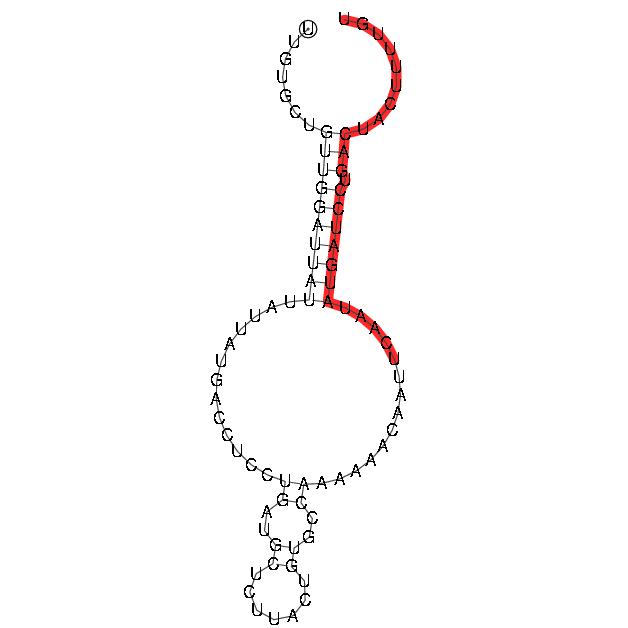

Supplement: Supplementary file 1 [file biology-13-00941-s001.zip › Data S2. Structures of novel miRNAs under treatment 1/novel_120_novel_120.jpg]

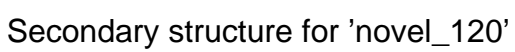

Supplement: Supplementary file 1 [file biology-13-00941-s001.zip › Data S2. Structures of novel miRNAs under treatment 1/novel_120_novel_120.pdf]

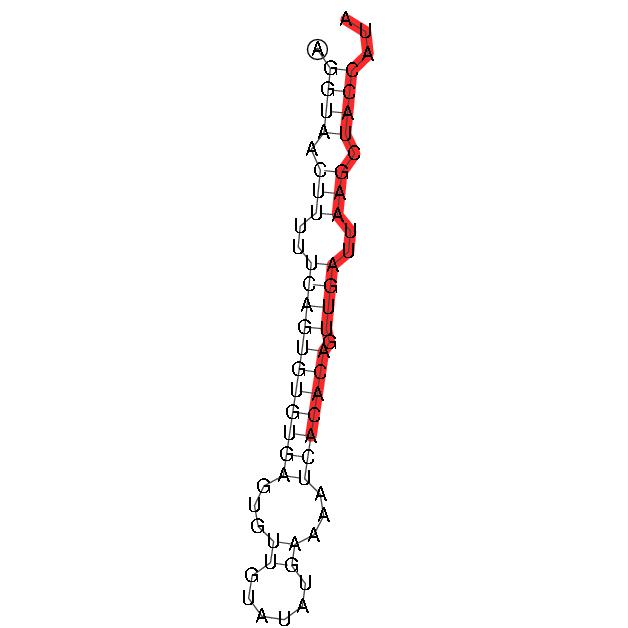

Supplement: Supplementary file 1 [file biology-13-00941-s001.zip › Data S2. Structures of novel miRNAs under treatment 1/novel_121_novel_121.jpg]

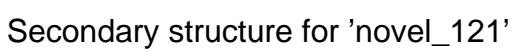

Supplement: Supplementary file 1 [file biology-13-00941-s001.zip › Data S2. Structures of novel miRNAs under treatment 1/novel_121_novel_121.pdf]

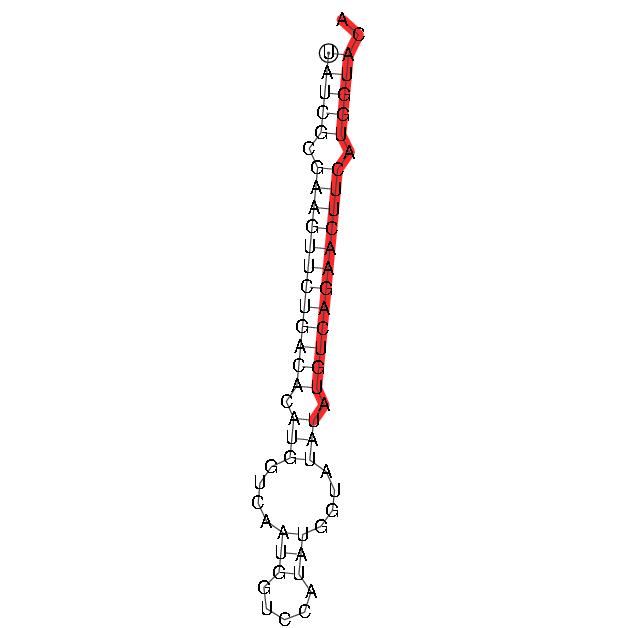

Supplement: Supplementary file 1 [file biology-13-00941-s001.zip › Data S2. Structures of novel miRNAs under treatment 1/novel_122_novel_122.jpg]

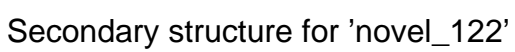

Supplement: Supplementary file 1 [file biology-13-00941-s001.zip › Data S2. Structures of novel miRNAs under treatment 1/novel_122_novel_122.pdf]

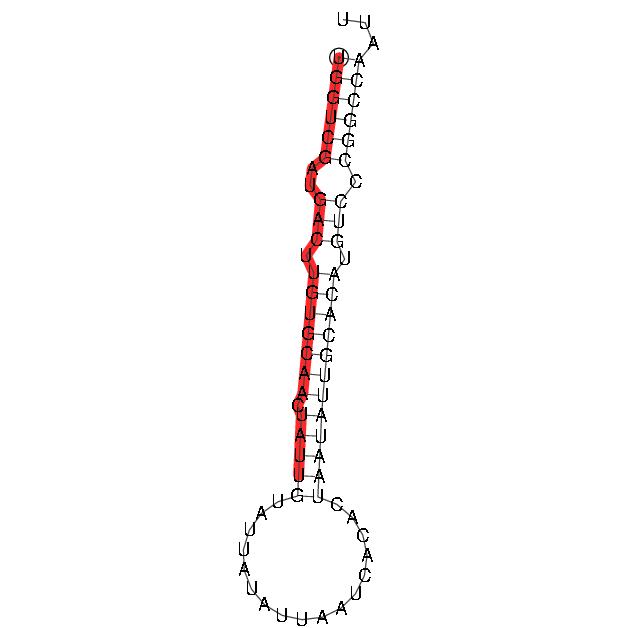

Supplement: Supplementary file 1 [file biology-13-00941-s001.zip › Data S2. Structures of novel miRNAs under treatment 1/novel_128_novel_128.jpg]

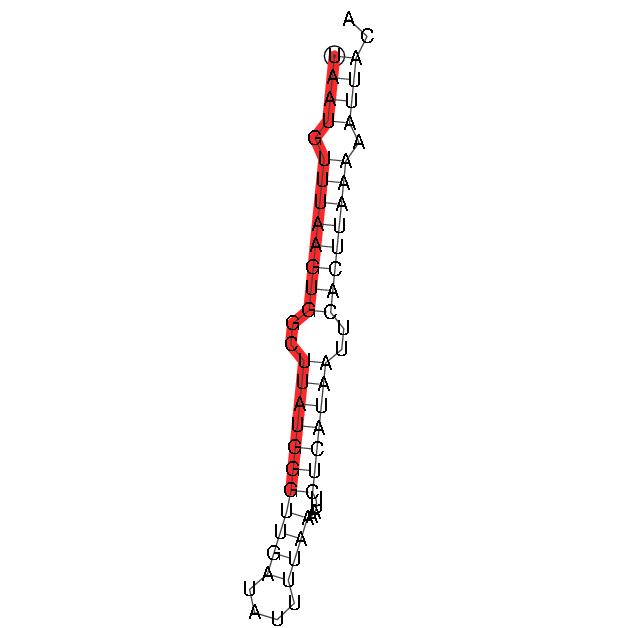

Supplement: Supplementary file 1 [file biology-13-00941-s001.zip › Data S2. Structures of novel miRNAs under treatment 1/novel_12_novel_12.jpg]

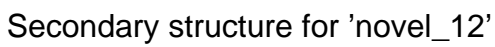

Supplement: Supplementary file 1 [file biology-13-00941-s001.zip › Data S2. Structures of novel miRNAs under treatment 1/novel_12_novel_12.pdf]

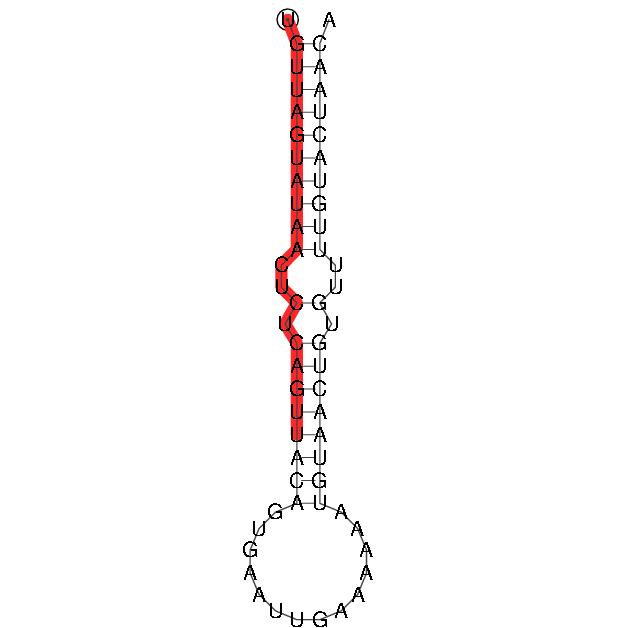

Supplement: Supplementary file 1 [file biology-13-00941-s001.zip › Data S2. Structures of novel miRNAs under treatment 1/novel_131_novel_131.jpg]

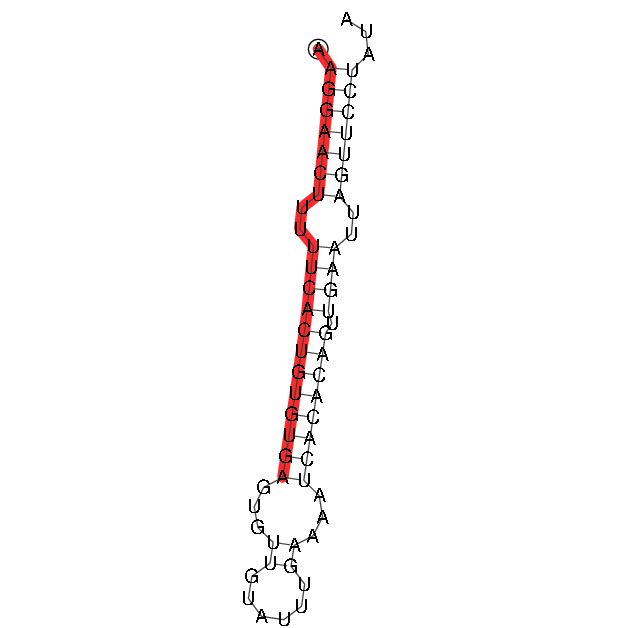

Supplement: Supplementary file 1 [file biology-13-00941-s001.zip › Data S2. Structures of novel miRNAs under treatment 1/novel_132_novel_132.jpg]

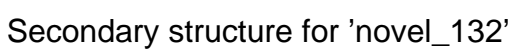

Supplement: Supplementary file 1 [file biology-13-00941-s001.zip › Data S2. Structures of novel miRNAs under treatment 1/novel_132_novel_132.pdf]

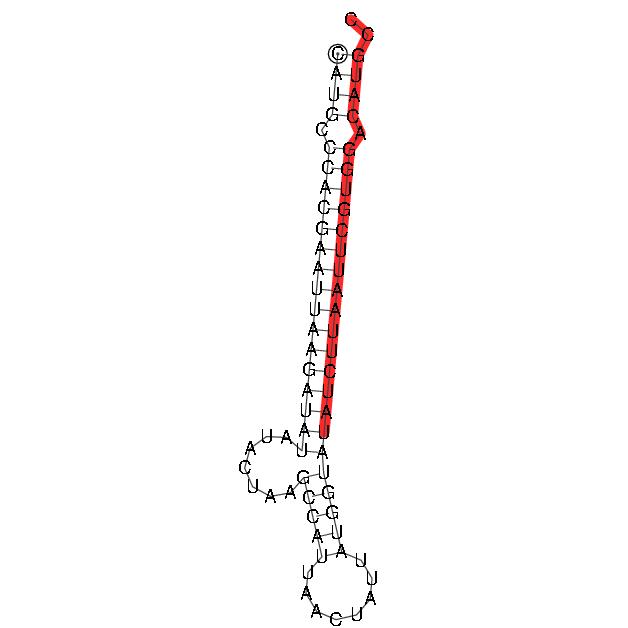

Supplement: Supplementary file 1 [file biology-13-00941-s001.zip › Data S2. Structures of novel miRNAs under treatment 1/novel_133_novel_133.jpg]

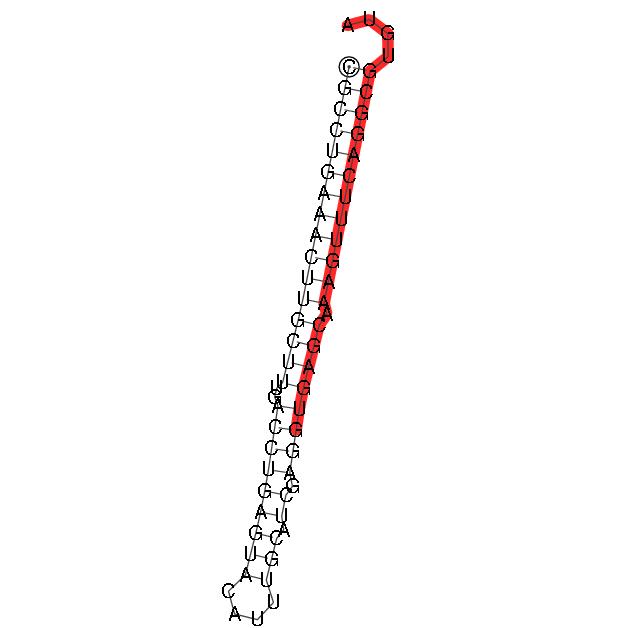

Supplement: Supplementary file 1 [file biology-13-00941-s001.zip › Data S2. Structures of novel miRNAs under treatment 1/novel_137_novel_137.jpg]

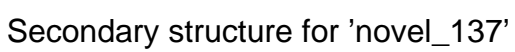

Supplement: Supplementary file 1 [file biology-13-00941-s001.zip › Data S2. Structures of novel miRNAs under treatment 1/novel_137_novel_137.pdf]

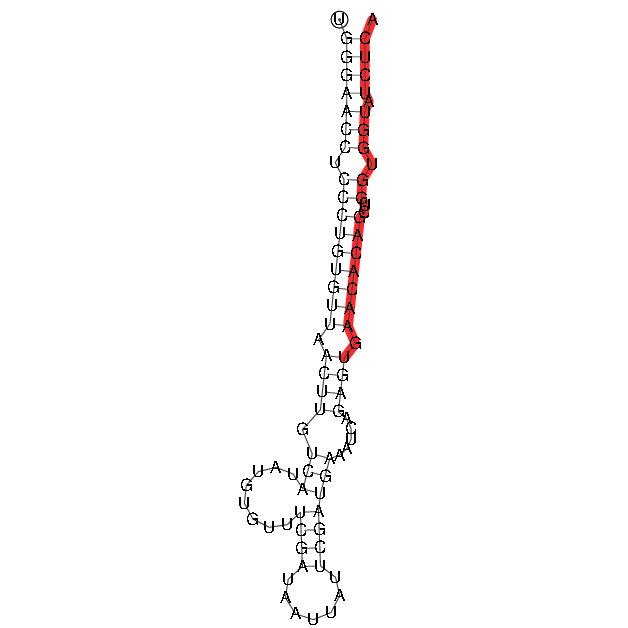

Supplement: Supplementary file 1 [file biology-13-00941-s001.zip › Data S2. Structures of novel miRNAs under treatment 1/novel_138_novel_138.jpg]

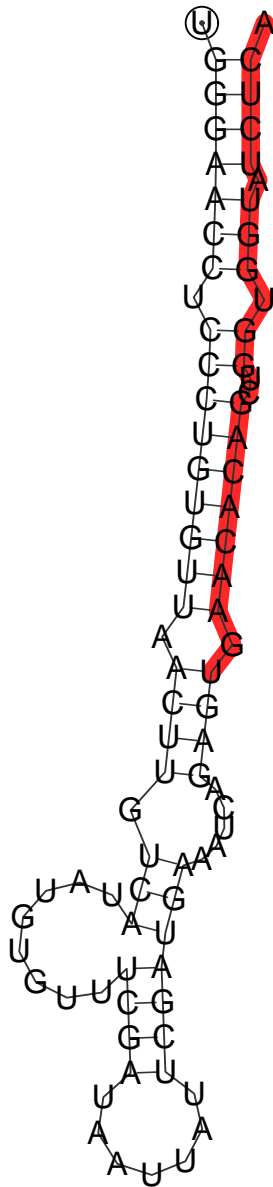

Secondary structure for 'novel\_138'

Supplement: Supplementary file 1 [file biology-13-00941-s001.zip › Data S2. Structures of novel miRNAs under treatment 1/novel_138_novel_138.pdf]

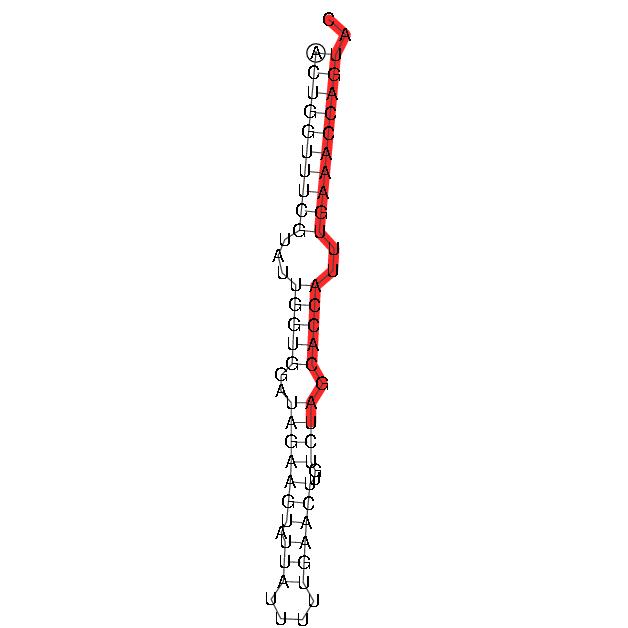

Supplement: Supplementary file 1 [file biology-13-00941-s001.zip › Data S2. Structures of novel miRNAs under treatment 1/novel_13_novel_13.jpg]

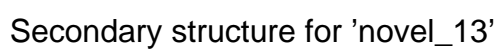

Supplement: Supplementary file 1 [file biology-13-00941-s001.zip › Data S2. Structures of novel miRNAs under treatment 1/novel_13_novel_13.pdf]

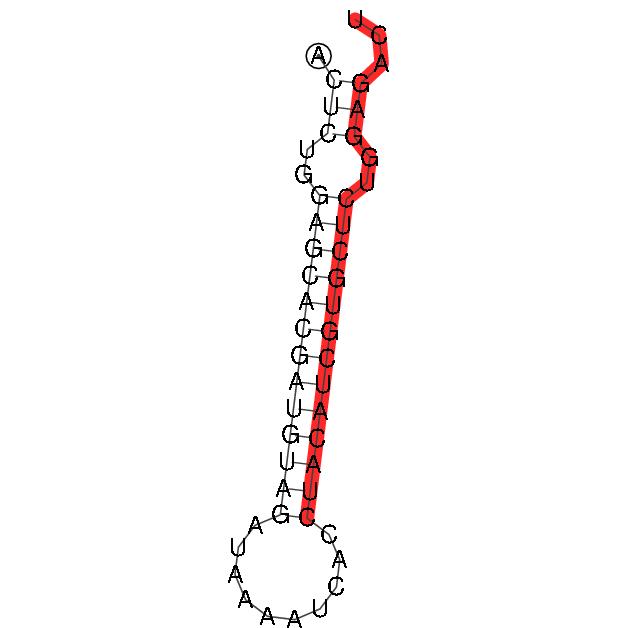

Supplement: Supplementary file 1 [file biology-13-00941-s001.zip › Data S2. Structures of novel miRNAs under treatment 1/novel_142_novel_142.jpg]

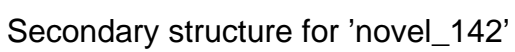

Supplement: Supplementary file 1 [file biology-13-00941-s001.zip › Data S2. Structures of novel miRNAs under treatment 1/novel_142_novel_142.pdf]

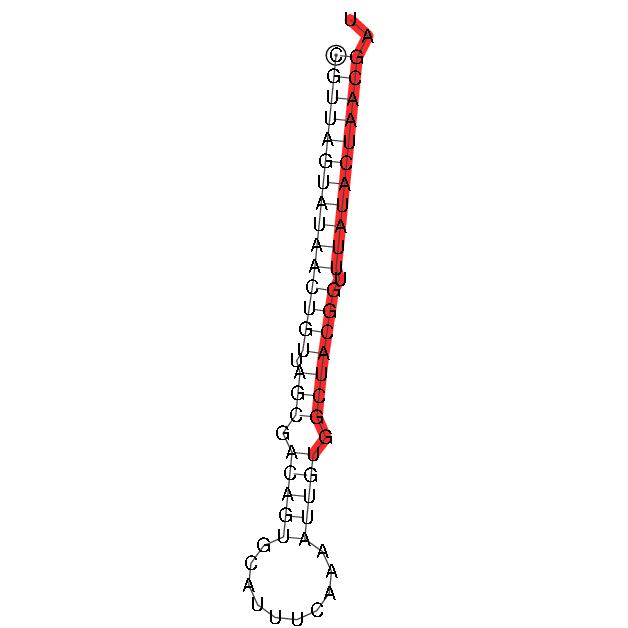

Supplement: Supplementary file 1 [file biology-13-00941-s001.zip › Data S2. Structures of novel miRNAs under treatment 1/novel_143_novel_143.jpg]

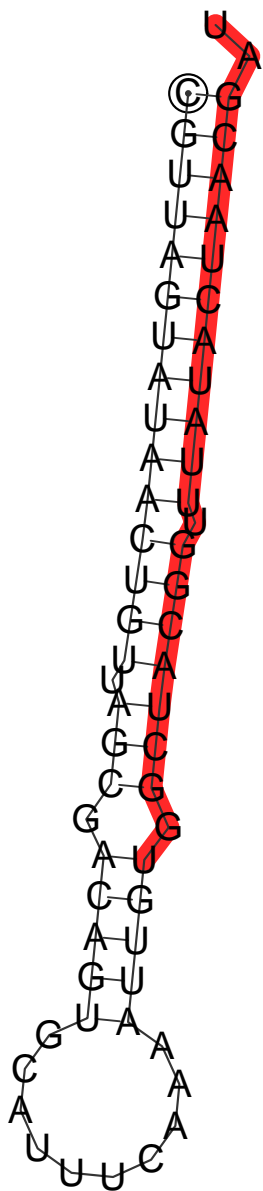

Secondary structure for 'novel\_143'

Supplement: Supplementary file 1 [file biology-13-00941-s001.zip › Data S2. Structures of novel miRNAs under treatment 1/novel_143_novel_143.pdf]

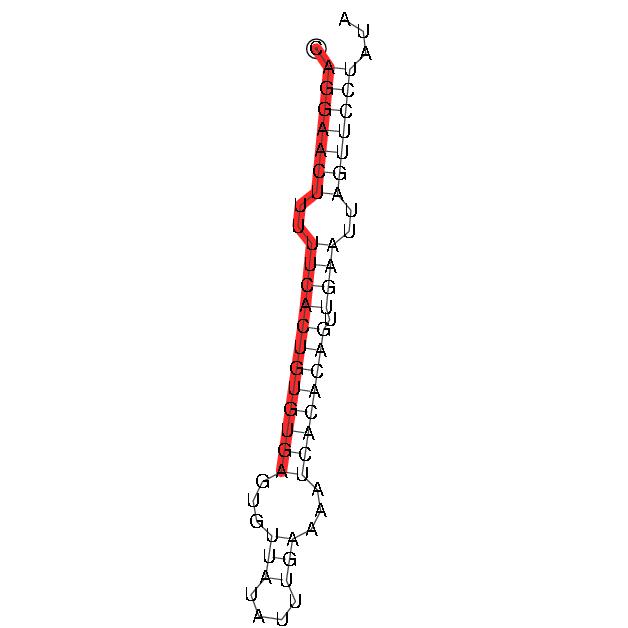

Supplement: Supplementary file 1 [file biology-13-00941-s001.zip › Data S2. Structures of novel miRNAs under treatment 1/novel_145_novel_145.jpg]

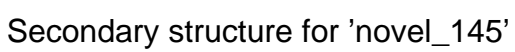

Supplement: Supplementary file 1 [file biology-13-00941-s001.zip › Data S2. Structures of novel miRNAs under treatment 1/novel_145_novel_145.pdf]

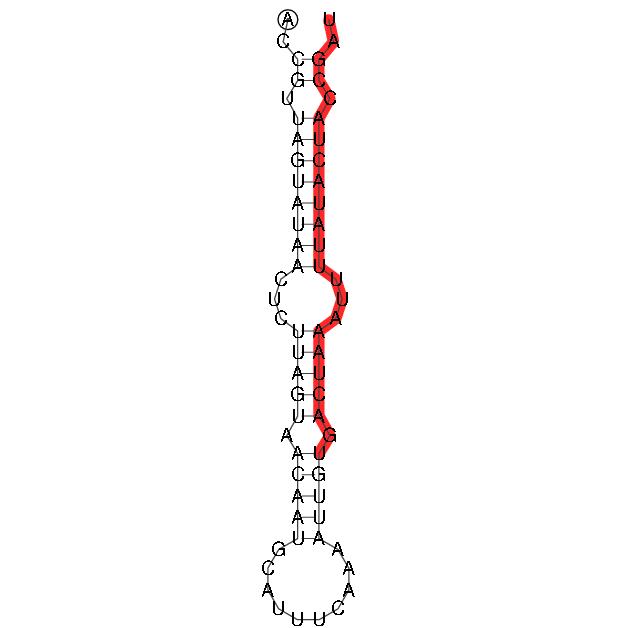

Supplement: Supplementary file 1 [file biology-13-00941-s001.zip › Data S2. Structures of novel miRNAs under treatment 1/novel_146_novel_146.jpg]

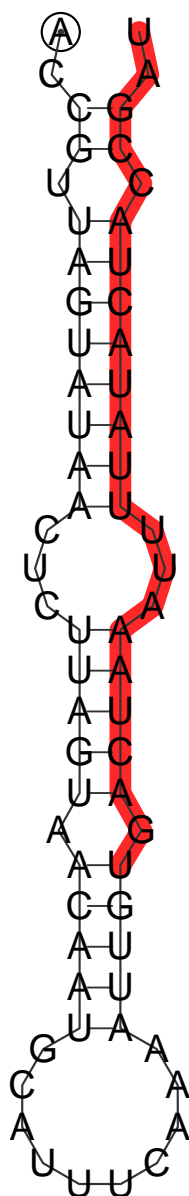

Secondary structure for 'novel\_146'

Supplement: Supplementary file 1 [file biology-13-00941-s001.zip › Data S2. Structures of novel miRNAs under treatment 1/novel_146_novel_146.pdf]

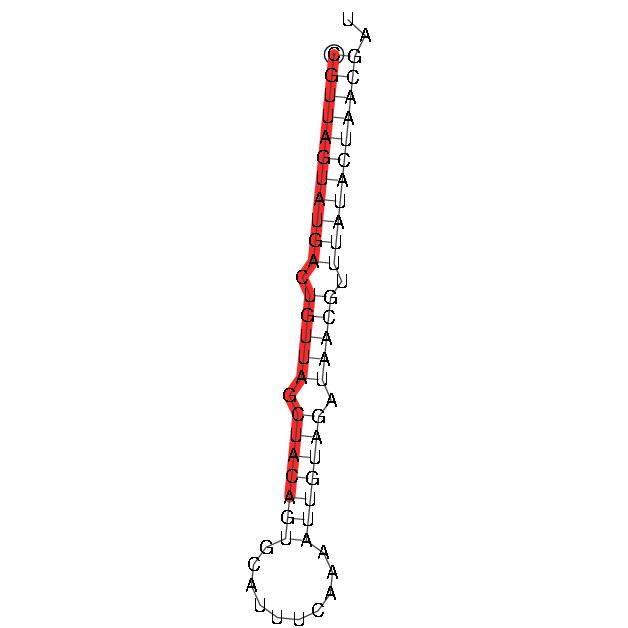

Supplement: Supplementary file 1 [file biology-13-00941-s001.zip › Data S2. Structures of novel miRNAs under treatment 1/novel_148_novel_148.jpg]

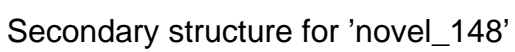

Supplement: Supplementary file 1 [file biology-13-00941-s001.zip › Data S2. Structures of novel miRNAs under treatment 1/novel_148_novel_148.pdf]

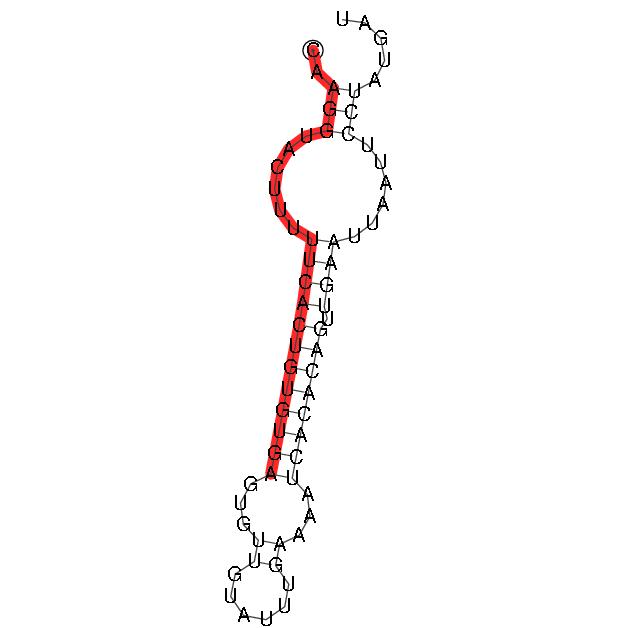

Supplement: Supplementary file 1 [file biology-13-00941-s001.zip › Data S2. Structures of novel miRNAs under treatment 1/novel_149_novel_149.jpg]

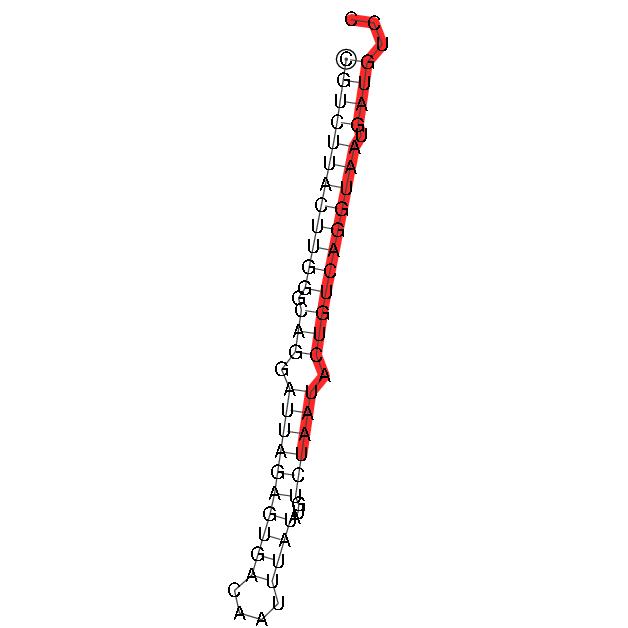

Supplement: Supplementary file 1 [file biology-13-00941-s001.zip › Data S2. Structures of novel miRNAs under treatment 1/novel_14_novel_14.jpg]

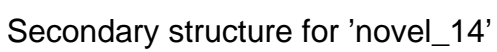

Supplement: Supplementary file 1 [file biology-13-00941-s001.zip › Data S2. Structures of novel miRNAs under treatment 1/novel_14_novel_14.pdf]

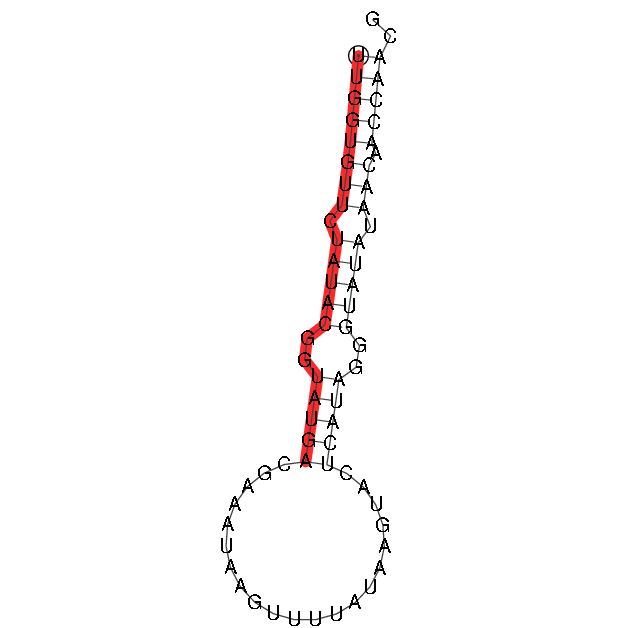

Supplement: Supplementary file 1 [file biology-13-00941-s001.zip › Data S2. Structures of novel miRNAs under treatment 1/novel_150_novel_150.jpg]

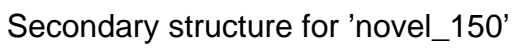

Supplement: Supplementary file 1 [file biology-13-00941-s001.zip › Data S2. Structures of novel miRNAs under treatment 1/novel_150_novel_150.pdf]

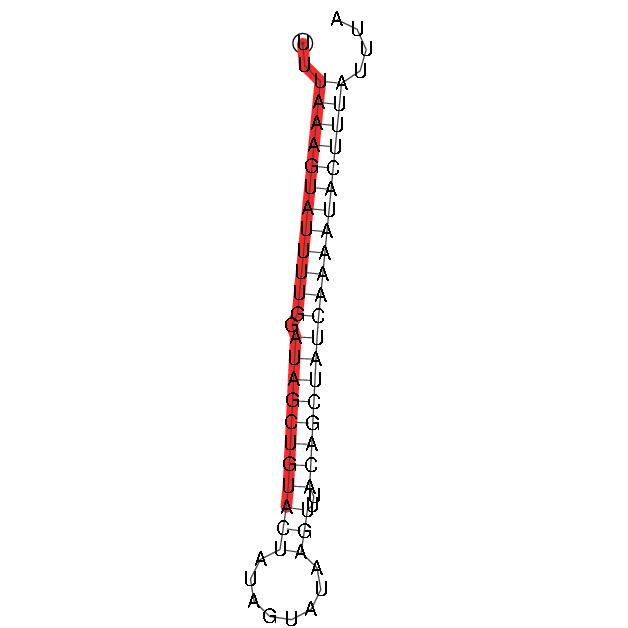

Supplement: Supplementary file 1 [file biology-13-00941-s001.zip › Data S2. Structures of novel miRNAs under treatment 1/novel_153_novel_153.jpg]

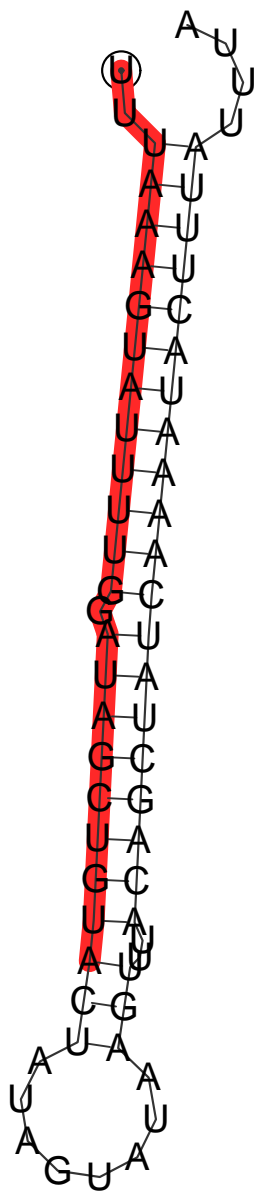

Secondary structure for 'novel\_153'

Supplement: Supplementary file 1 [file biology-13-00941-s001.zip › Data S2. Structures of novel miRNAs under treatment 1/novel_153_novel_153.pdf]

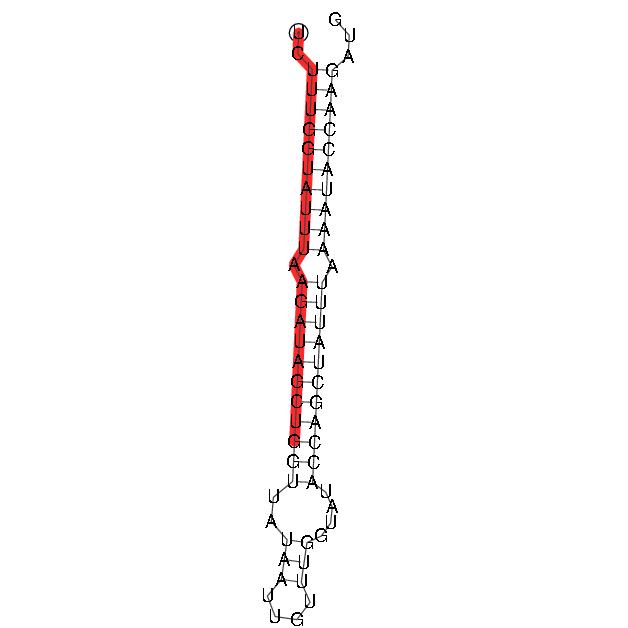

Supplement: Supplementary file 1 [file biology-13-00941-s001.zip › Data S2. Structures of novel miRNAs under treatment 1/novel_154_novel_154.jpg]

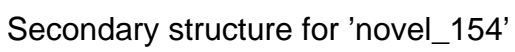

Supplement: Supplementary file 1 [file biology-13-00941-s001.zip › Data S2. Structures of novel miRNAs under treatment 1/novel_154_novel_154.pdf]

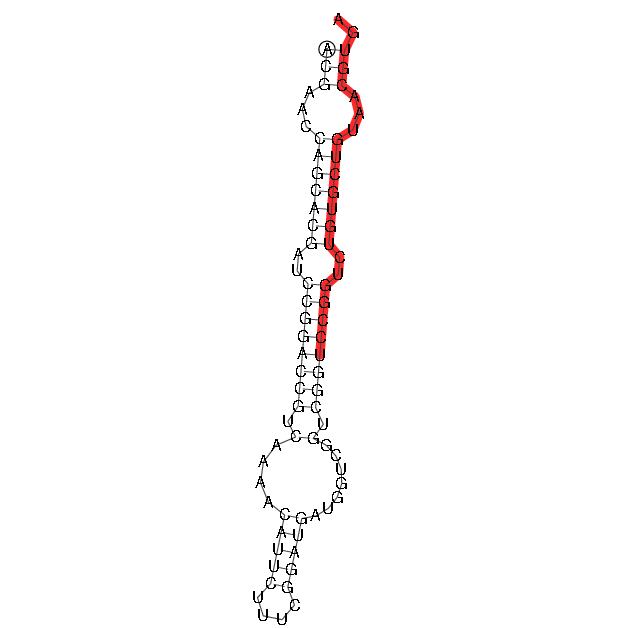

Supplement: Supplementary file 1 [file biology-13-00941-s001.zip › Data S2. Structures of novel miRNAs under treatment 1/novel_158_novel_158.jpg]

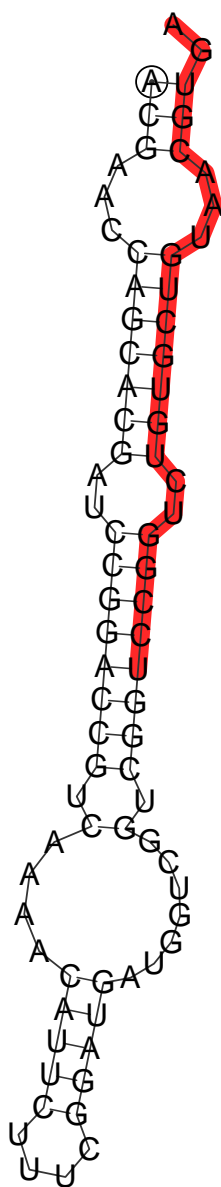

Secondary structure for 'novel\_158'

Supplement: Supplementary file 1 [file biology-13-00941-s001.zip › Data S2. Structures of novel miRNAs under treatment 1/novel_158_novel_158.pdf]

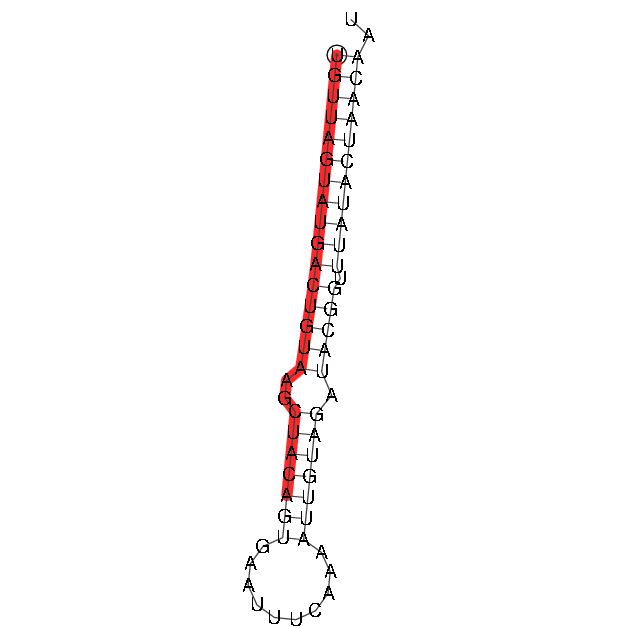

Supplement: Supplementary file 1 [file biology-13-00941-s001.zip › Data S2. Structures of novel miRNAs under treatment 1/novel_159_novel_159.jpg]

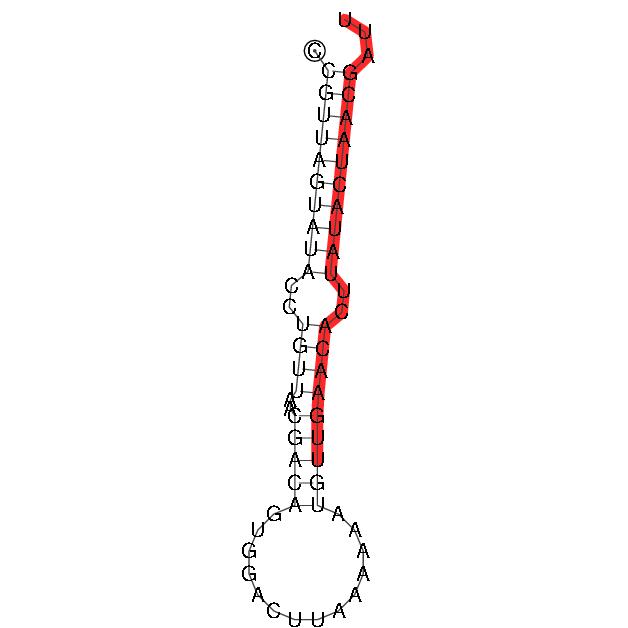

Supplement: Supplementary file 1 [file biology-13-00941-s001.zip › Data S2. Structures of novel miRNAs under treatment 1/novel_161_novel_161.jpg]

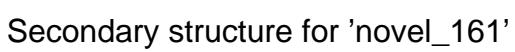

Supplement: Supplementary file 1 [file biology-13-00941-s001.zip › Data S2. Structures of novel miRNAs under treatment 1/novel_161_novel_161.pdf]

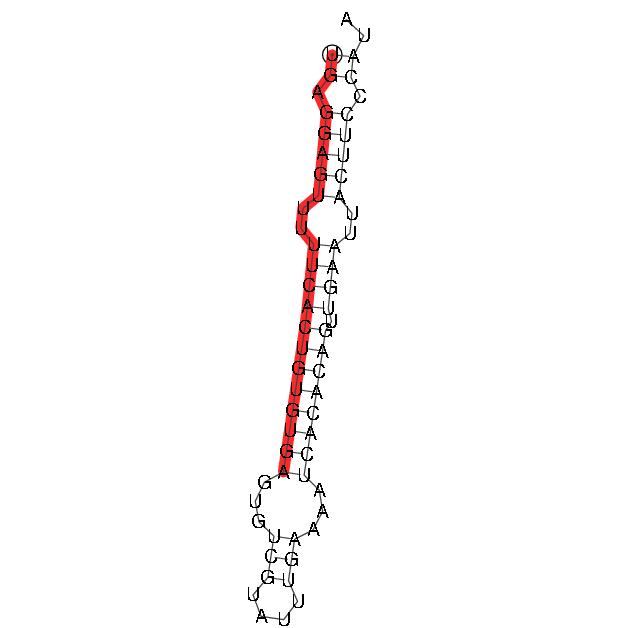

Supplement: Supplementary file 1 [file biology-13-00941-s001.zip › Data S2. Structures of novel miRNAs under treatment 1/novel_163_novel_163.jpg]

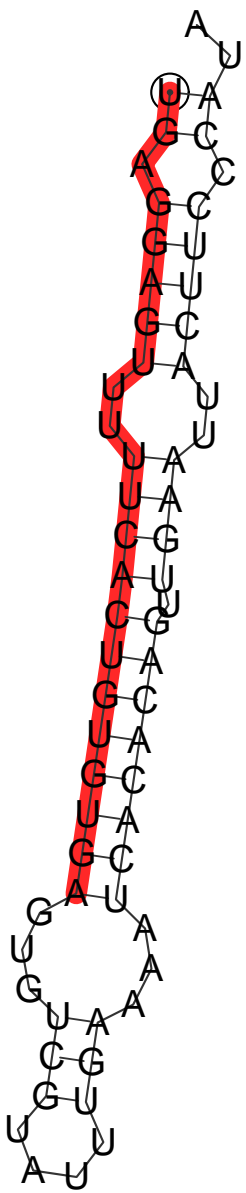

Secondary structure for 'novel\_163'

Supplement: Supplementary file 1 [file biology-13-00941-s001.zip › Data S2. Structures of novel miRNAs under treatment 1/novel_163_novel_163.pdf]

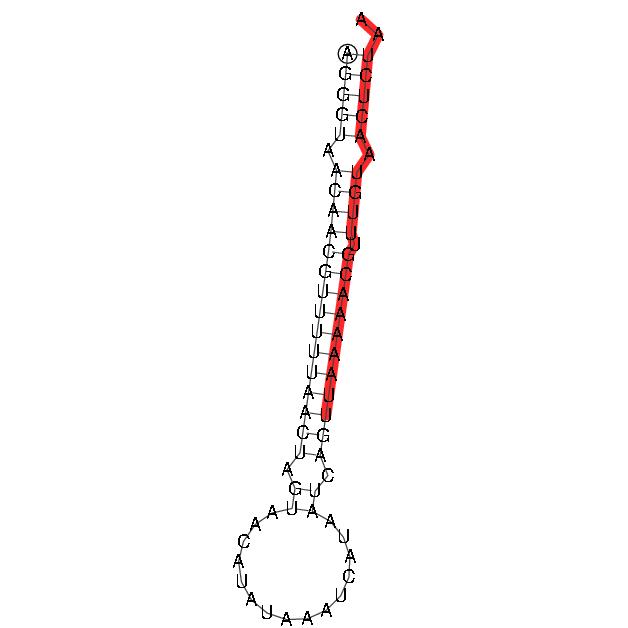

Supplement: Supplementary file 1 [file biology-13-00941-s001.zip › Data S2. Structures of novel miRNAs under treatment 1/novel_164_novel_164.jpg]

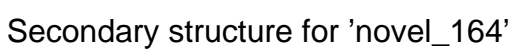

Supplement: Supplementary file 1 [file biology-13-00941-s001.zip › Data S2. Structures of novel miRNAs under treatment 1/novel_164_novel_164.pdf]

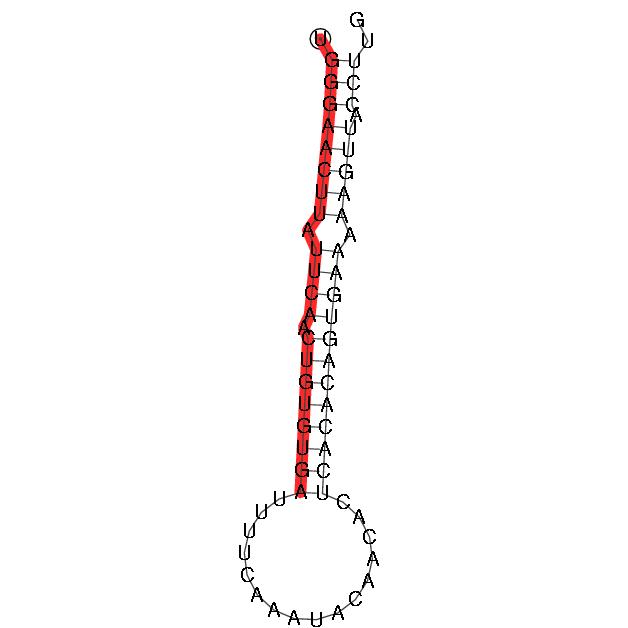

Supplement: Supplementary file 1 [file biology-13-00941-s001.zip › Data S2. Structures of novel miRNAs under treatment 1/novel_166_novel_166.jpg]

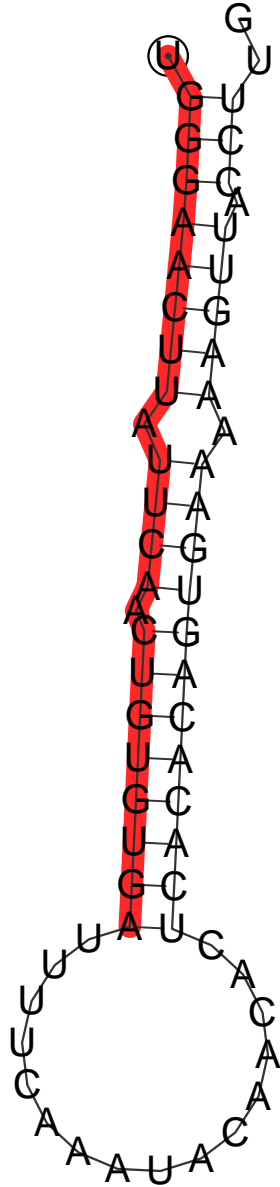

Supplement: Supplementary file 1 [file biology-13-00941-s001.zip › Data S2. Structures of novel miRNAs under treatment 1/novel_166_novel_166.pdf]

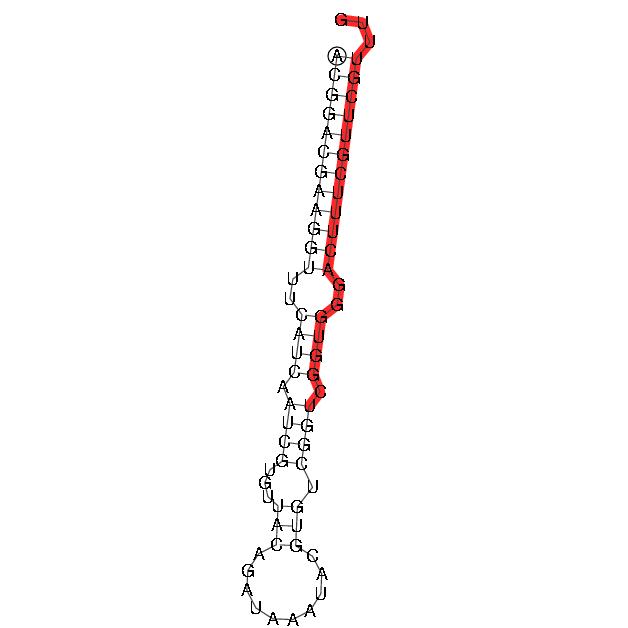

Supplement: Supplementary file 1 [file biology-13-00941-s001.zip › Data S2. Structures of novel miRNAs under treatment 1/novel_16_novel_16.jpg]

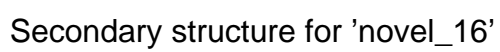

Supplement: Supplementary file 1 [file biology-13-00941-s001.zip › Data S2. Structures of novel miRNAs under treatment 1/novel_16_novel_16.pdf]

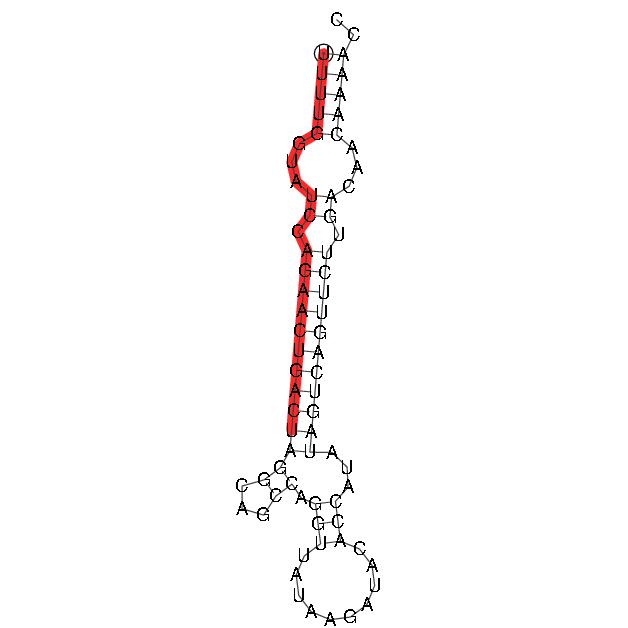

Supplement: Supplementary file 1 [file biology-13-00941-s001.zip › Data S2. Structures of novel miRNAs under treatment 1/novel_170_novel_170.jpg]

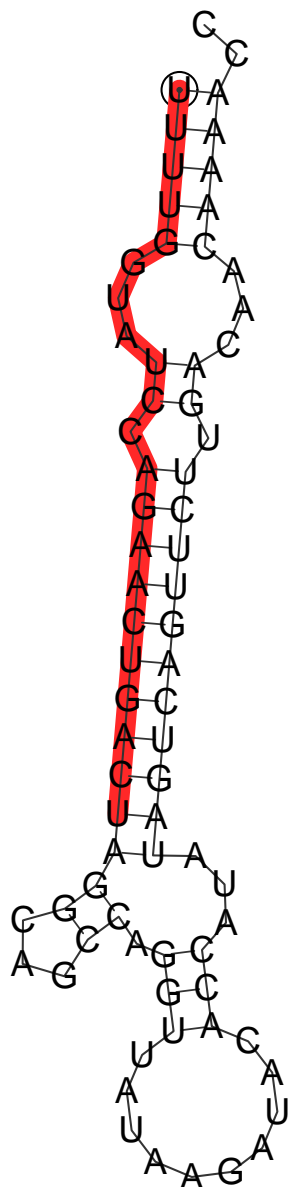

Secondary structure for 'novel\_170'

Supplement: Supplementary file 1 [file biology-13-00941-s001.zip › Data S2. Structures of novel miRNAs under treatment 1/novel_170_novel_170.pdf]

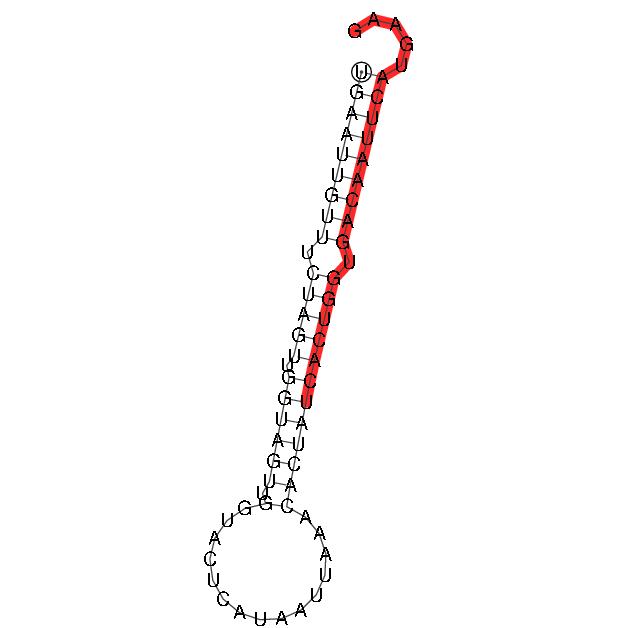

Supplement: Supplementary file 1 [file biology-13-00941-s001.zip › Data S2. Structures of novel miRNAs under treatment 1/novel_171_novel_171.jpg]

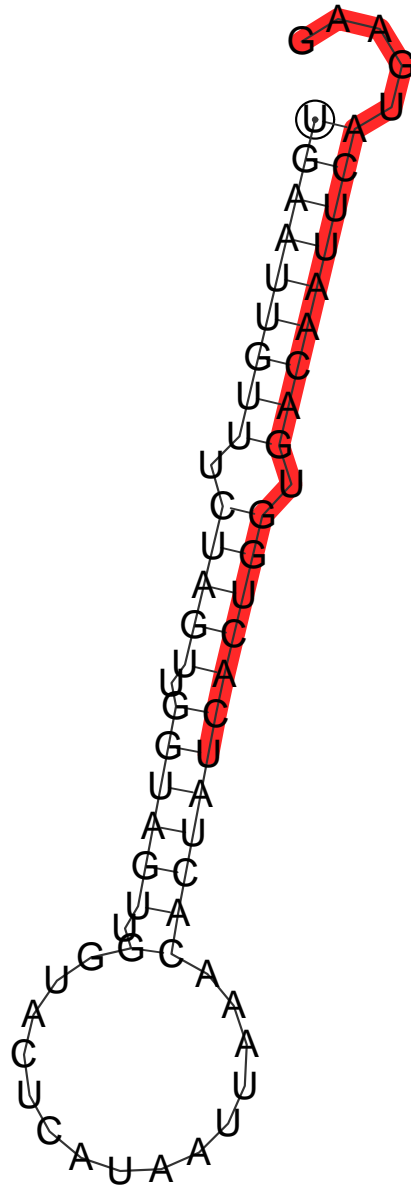

Secondary structure for 'novel\_171'

Supplement: Supplementary file 1 [file biology-13-00941-s001.zip › Data S2. Structures of novel miRNAs under treatment 1/novel_171_novel_171.pdf]

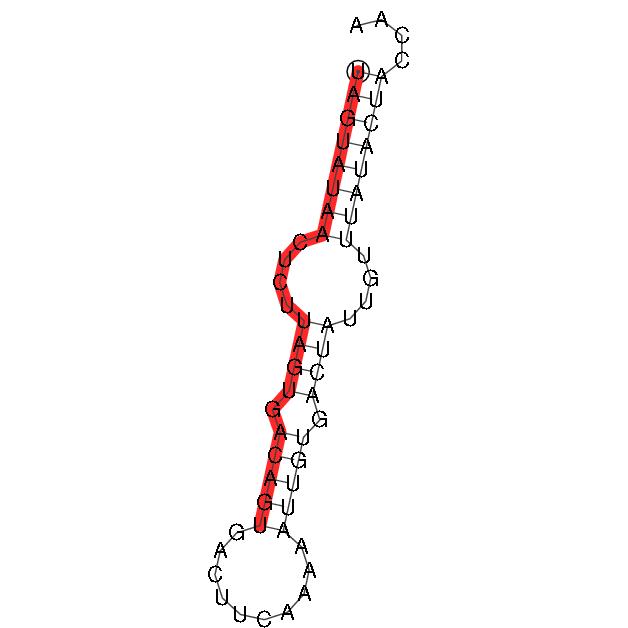

Supplement: Supplementary file 1 [file biology-13-00941-s001.zip › Data S2. Structures of novel miRNAs under treatment 1/novel_172_novel_172.jpg]

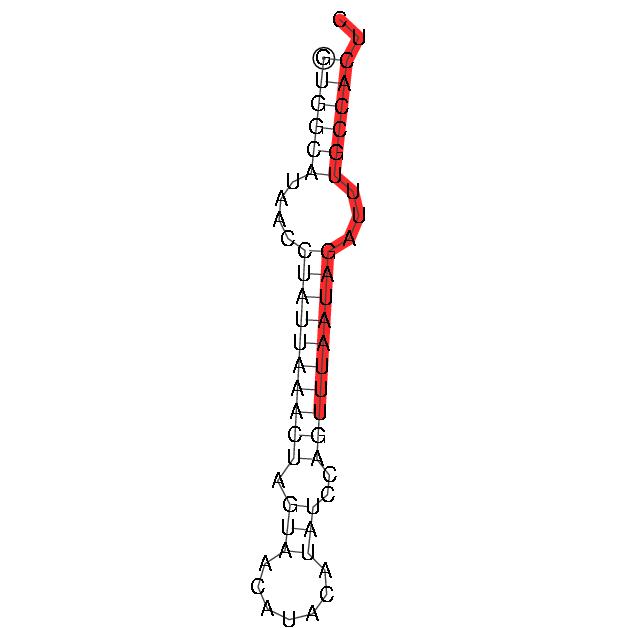

Supplement: Supplementary file 1 [file biology-13-00941-s001.zip › Data S2. Structures of novel miRNAs under treatment 1/novel_173_novel_173.jpg]

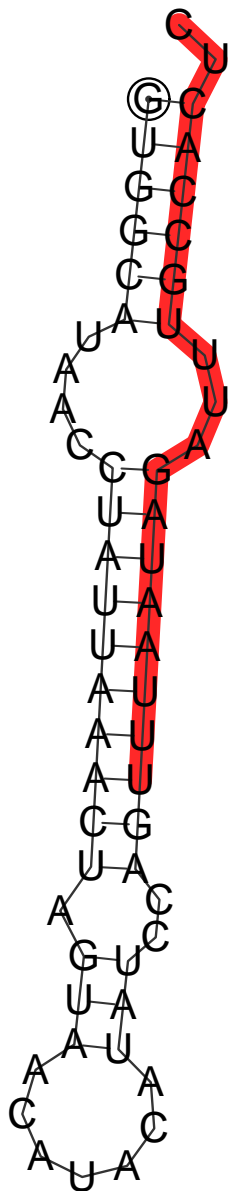

Supplement: Supplementary file 1 [file biology-13-00941-s001.zip › Data S2. Structures of novel miRNAs under treatment 1/novel_173_novel_173.pdf]

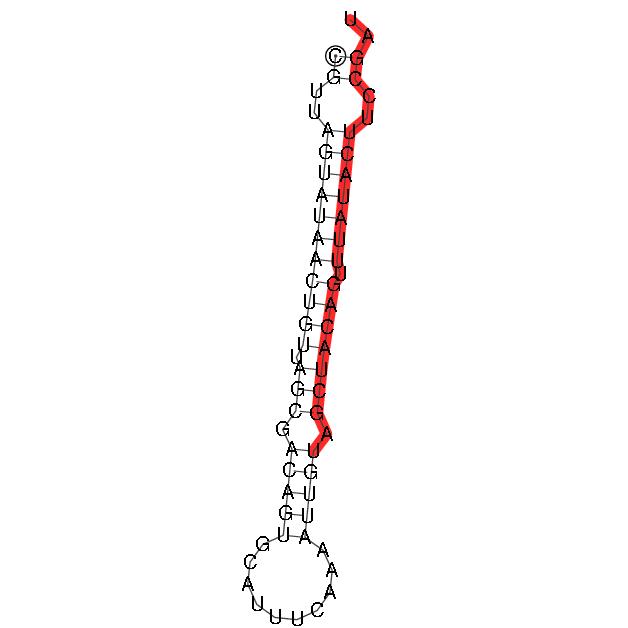

Supplement: Supplementary file 1 [file biology-13-00941-s001.zip › Data S2. Structures of novel miRNAs under treatment 1/novel_177_novel_177.jpg]

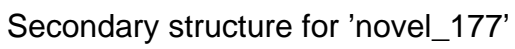

Supplement: Supplementary file 1 [file biology-13-00941-s001.zip › Data S2. Structures of novel miRNAs under treatment 1/novel_177_novel_177.pdf]

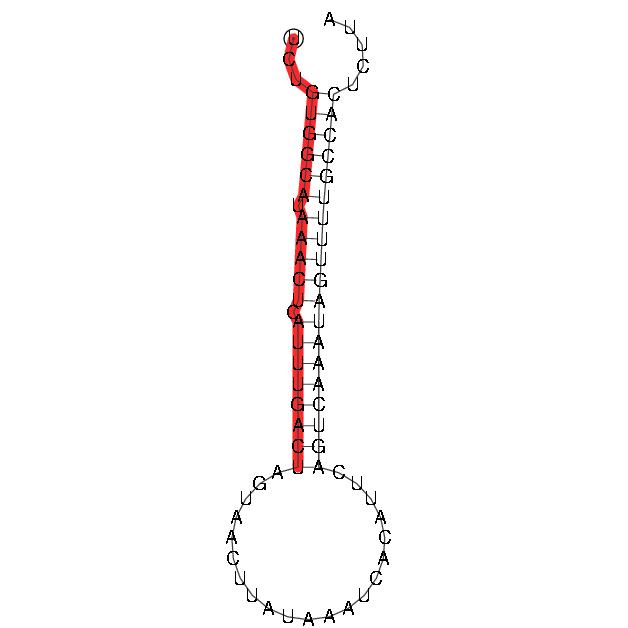

Supplement: Supplementary file 1 [file biology-13-00941-s001.zip › Data S2. Structures of novel miRNAs under treatment 1/novel_179_novel_179.jpg]

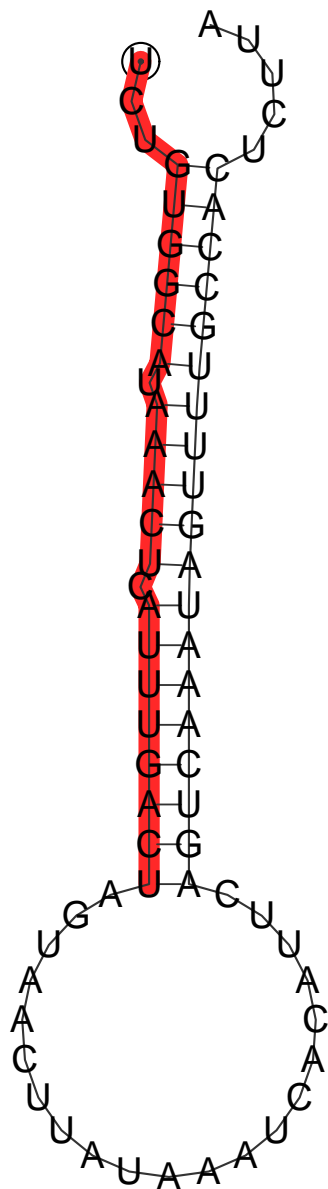

Secondary structure for 'novel\_179'

Supplement: Supplementary file 1 [file biology-13-00941-s001.zip › Data S2. Structures of novel miRNAs under treatment 1/novel_179_novel_179.pdf]

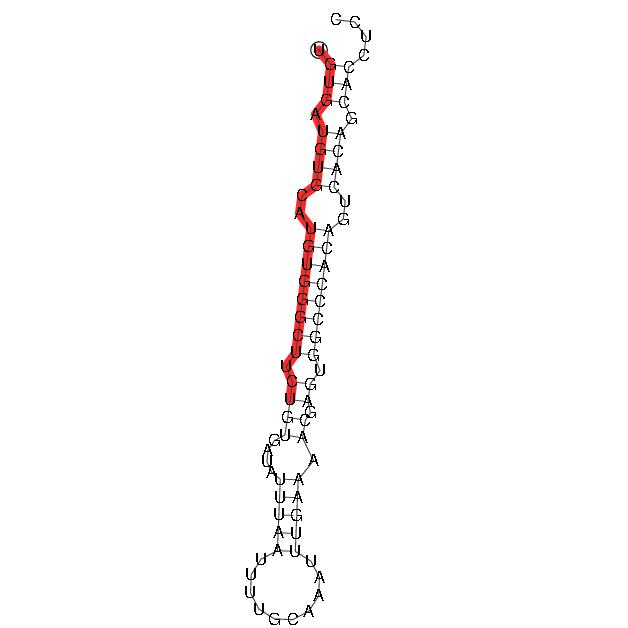

Supplement: Supplementary file 1 [file biology-13-00941-s001.zip › Data S2. Structures of novel miRNAs under treatment 1/novel_17_novel_17.jpg]

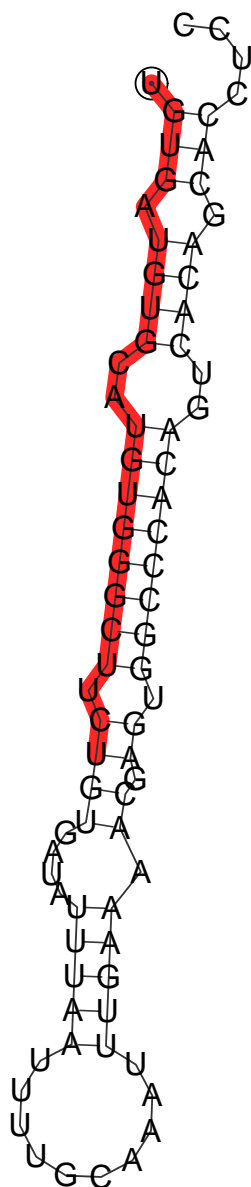

Secondary structure for 'novel\_17'

Supplement: Supplementary file 1 [file biology-13-00941-s001.zip › Data S2. Structures of novel miRNAs under treatment 1/novel_17_novel_17.pdf]

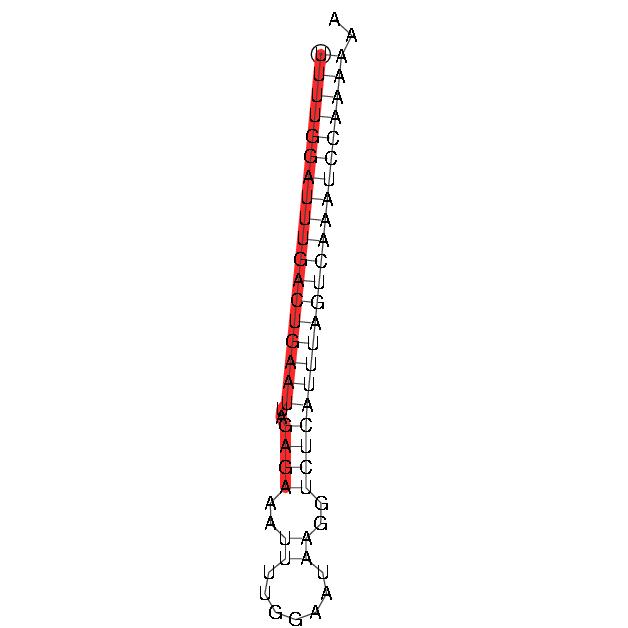

Supplement: Supplementary file 1 [file biology-13-00941-s001.zip › Data S2. Structures of novel miRNAs under treatment 1/novel_180_novel_180.jpg]

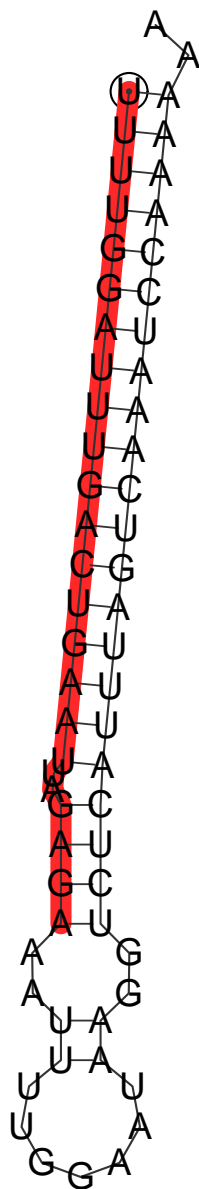

Secondary structure for 'novel\_180'

Supplement: Supplementary file 1 [file biology-13-00941-s001.zip › Data S2. Structures of novel miRNAs under treatment 1/novel_180_novel_180.pdf]

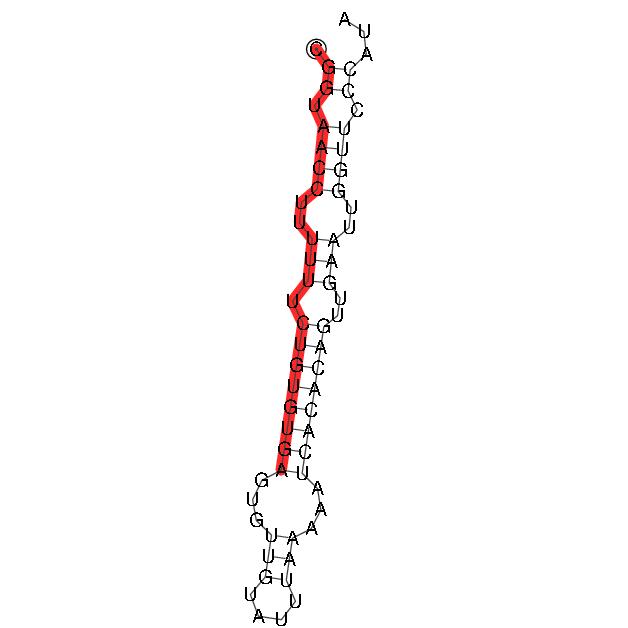

Supplement: Supplementary file 1 [file biology-13-00941-s001.zip › Data S2. Structures of novel miRNAs under treatment 1/novel_182_novel_182.jpg]

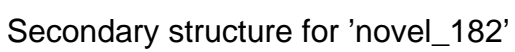

Supplement: Supplementary file 1 [file biology-13-00941-s001.zip › Data S2. Structures of novel miRNAs under treatment 1/novel_182_novel_182.pdf]

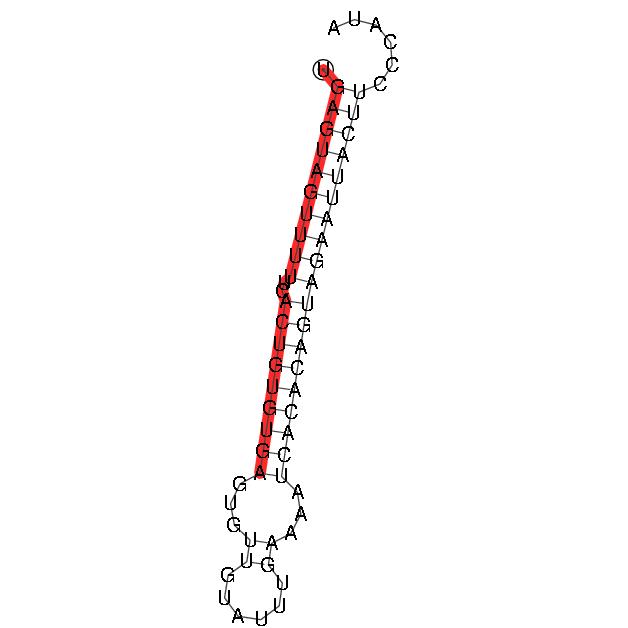

Supplement: Supplementary file 1 [file biology-13-00941-s001.zip › Data S2. Structures of novel miRNAs under treatment 1/novel_183_novel_183.jpg]

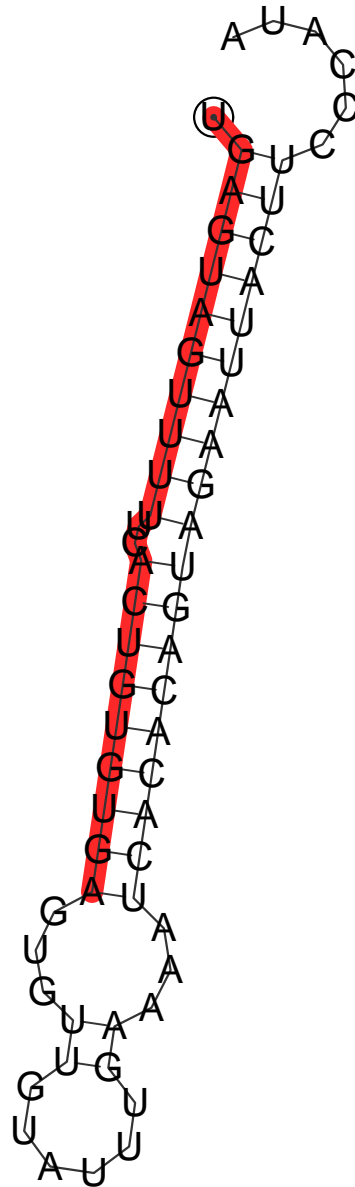

Secondary structure for 'novel\_183'

Supplement: Supplementary file 1 [file biology-13-00941-s001.zip › Data S2. Structures of novel miRNAs under treatment 1/novel_183_novel_183.pdf]

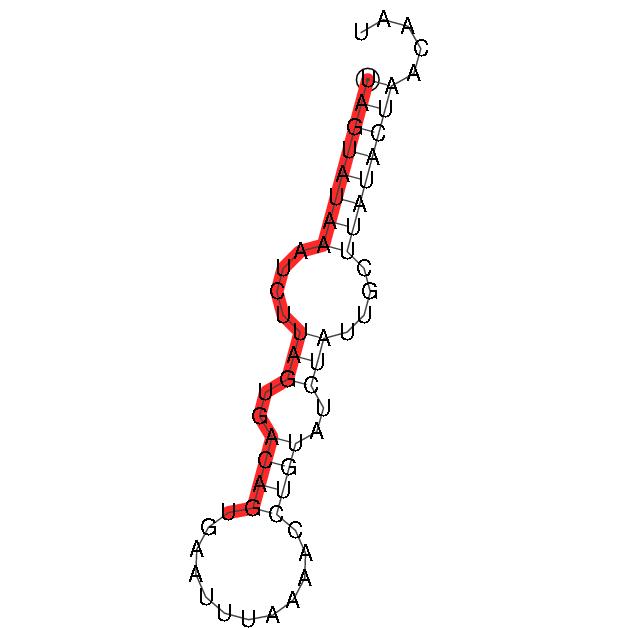

Supplement: Supplementary file 1 [file biology-13-00941-s001.zip › Data S2. Structures of novel miRNAs under treatment 1/novel_186_novel_186.jpg]

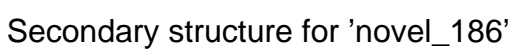

Supplement: Supplementary file 1 [file biology-13-00941-s001.zip › Data S2. Structures of novel miRNAs under treatment 1/novel_186_novel_186.pdf]
