# Supplementary material for: Molecular Characterization of miRNAs in Myzus persicae Carrying Brassica Yellows Virus
Source: Biology (Basel). 2024 Nov 18;13(11):941. doi: 10.3390/biology13110941 (PMC11591976; doi:10.3390/biology13110941)
Supplement: Supplementary file 1 [file novel_131_novel_131.pdf › Data S2. Structures of novel miRNAs under treatment 1/novel_131_novel_131.pdf]

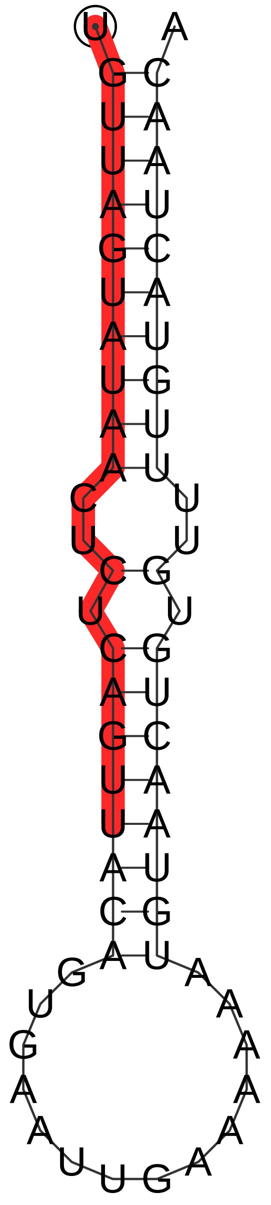

Secondary structure for ’novel_131’
